# Interaction of family SES with children’s genetic propensity for cognitive and noncognitive skills: No evidence of the Scarr-Rowe hypothesis for educational outcomes^☆^

**DOI:** 10.1016/j.rssm.2024.100960

**Published:** 2024-08

**Authors:** Gaia Ghirardi, Carlos J. Gil-Hernández, Fabrizio Bernardi, Elsje van Bergen, Perline Demange

**Affiliations:** a Department of Political and Social Sciences, European University Institute (EUI), Florence, Italy; b Department of Statistical Sciences, University of Bologna, Bologna, Italy; c European Commission, Centre for Advanced Studies, Joint Research Centre, Sevilla, Spain; d Department of Statistics, Computer Science, Applications, University of Florence, Florence, Italy; e Department of Sociology II, Universidad Nacional de Educación a Distancia (UNED), Madrid, Spain; f Department of Biological Psychology, Vrije Universiteit (VU), Amsterdam, the Netherlands; g Department of Psychology, University of Oslo, Oslo, Norway

**Keywords:** Gene-environment (G × E) interaction, Educational inequality, Scarr-Rowe hypothesis, Compensatory advantage hypothesis, Between-family analysis, Within-family analysis, Trio analysis, Netherlands Twin Register

## Abstract

This study examines the role of genes and environments in predicting educational outcomes. We test the Scarr-Rowe hypothesis, suggesting that enriched environments enable genetic potential to unfold, and the compensatory advantage hypothesis, proposing that low genetic endowments have less impact on education for children from high socioeconomic status (SES) families. We use a pre-registered design with *Netherlands Twin Register* data (426 ≤ *N*_individuals_ ≤ 3875). We build polygenic indexes (PGIs) for cognitive and noncognitive skills to predict seven educational outcomes from childhood to adulthood across three designs (between-family, within-family, and trio) accounting for different confounding sources, totalling 42 analyses. Cognitive PGIs, noncognitive PGIs, and parental education positively predict educational outcomes. Providing partial support for the compensatory hypothesis, 39/42 PGI × SES interactions are negative, with 7 reaching statistical significance under Romano-Wolf and 3 under the more conservative Bonferroni multiple testing corrections (p-value < 0.007). In contrast, the Scarr-Rowe hypothesis lacks empirical support, with just 2 non-significant and 1 significant (not surviving Romano-Wolf) positive interactions. Overall, we emphasise the need for future replication studies in larger samples. Our findings demonstrate the value of merging social-stratification and behavioural-genetic theories to better understand the intricate interplay between genetic factors and social contexts.

## Highlights

• Genes and family socio-economic status (SES) interact in predicting educational outcomes.
• The Scarr-Rowe hypothesis states that high-SES families enable children to fully express their genetic potential.
• The compensatory advantage hypothesis claims that low genetic endowments have less impact on education for high-SES children.
• We build polygenic indexes for cognitive and noncognitive skills to predict seven outcomes across three research designs.
• The Scarr-Rowe hypothesis lacks empirical support, while the compensatory advantage hypothesis is partially validated.

## 1 Introduction

Educational attainment influences future individuals’ socioeconomic status (SES) and health outcomes. Therefore, extensive research has investigated the intergenerational determinants of educational success. In social stratification research, the importance of family SES^2^ in reproducing educational opportunities over generations is a stylised fact (Blossfeld et al., 2016, Breen and Jonsson, 2005). Environmental inequalities in cultural and economic resources and investments would largely explain why children from high-SES families are more likely to succeed in schools than their low-SES peers (Jackson, 2013). At the same time, from behavioural genetics scholarship, it is well established that individual differences in nearly every phenotype of interest to social scientists are heritable to some degree (Polderman et al., 2015, Turkheimer, 2000, Van Hootegem et al., 2024). Classical twin studies (Branigan et al., 2013, Silventoinen et al., 2020) and novel molecular studies directly measuring the genome (Okbay et al., 2022, Lee et al., 2018) show that educational attainment is no exception, with genetics explaining between 40 % and 16 % of the variance, respectively.

While the fact that both genes and environments influence educational attainment within generations is clear (Nielsen, 2016, Conley et al., 2015, Diewald et al., 2015), an interdisciplinary research area on gene-environment interactions (G × E) further scrutinises whether the effect of genetic variants on a phenotype depends on the environment where individuals are raised or schooled—and vice versa (Conley et al., 2015, Shanahan and Hofer, 2005). Nevertheless, whether genetic propensity for education matters more among children from high or low-SES families is still an open question from a theoretical and empirical viewpoint (Domingue et al., 2020).

Theoretically, two main competing hypotheses from behavioural genetics and social stratification research streams predict G × E interactions on educational attainment, outlining different patterns and mechanisms (Ruks, 2022). The Scarr-Rowe hypothesis (Scarr-Salapatek, 1971) from behavioural genetics posits that enriched social environments allow individuals to fully express their genetic potential so that genes are more predictive of cognitive performance among socioeconomically advantaged families (Rowe et al., 1999). Alternatively, the compensatory advantage hypothesis (Bernardi, 2014) from social stratification predicts that negative traits or events for status attainment—e.g., a low genetic propensity for education—are less detrimental to high-SES children’s educational attainment due to well-off parents’ aversion to downward mobility and compensatory strategies (Breen & Goldthorpe, 1997; Holm et al., 2019).

Empirically, a growing interdisciplinary literature spanning sociology, psychology and economics is accumulating evidence around G × E interactions. Previous research from classical twin models (Turkheimer and Horn, 2014, Turkheimer et al., 2003) and new advancements in molecular genetics scholarship (Conley et al., 2015) reach mixed conclusions on whether advantaged families fully express or compensate their children’s genetic endowments for education. In line with the Scarr-Rowe hypothesis, several twin (Baier and Lang, 2019, Harden et al., 2007) and molecular genetics studies (Ronda et al., 2022, Uchikoshi and Conley, 2021) show that genes are more predictive of intelligence or educational attainment among socioeconomically advantaged individuals. A meta-analysis by Tucker-Drob and Bates (2016) supports the Scarr-Rowe hypothesis for intelligence and test scores in the US, but it was not replicated in Western Europe or Australia. Conversely, in line with the compensatory hypothesis, some studies find a steeper genetic association among low-SES individuals, neighbourhoods, or schooling environments (Arold et al., 2022, Baier et al., 2022, Cheesman et al., 2022, Cheesman et al., 2022, Harden et al., 2020, Knigge et al., 2022, Lin, 2020, Ruks, 2022, Stienstra and Karlson, 2023, Trejo et al., 2018). Other authors find null effects or no consistent G × E interactions (Breinholt et al., 2023, Malanchini et al., 2023, Baier et al., 2022, Stienstra et al., 2021, Isungset et al., 2021, de Zeeuw et al., 2019, Figlio et al., 2017, Conley et al., 2015).

In this article, we examine whether parental SES moderates the effect of children’s genetic propensity for education on different educational outcomes over the life course. Precisely, we contribute to the literature by testing whether previously mixed findings on G × E interactions in educational attainment might be related to three main aspects: (1) the type of research design implemented, (2) the measures of genetic endowments studied, and (3) the type and timing of the educational outcomes analysed.

First, we implemented several research designs. One central challenge in detecting unbiased G × E interactions is to control for the endogeneity between social contexts and genetic endowments, known as gene-environment correlations (rGE). Each parent transmits, on average, 50 % of their genetic material to their offspring. Some of these genes also affect the environment where children are raised, for instance, family SES and parenting practices (Marks and O’Connell, 2023, Kong et al., 2018). That leads to a correlation between the family’s SES characteristics and the child’s genetics, named passive rGE (Hart et al., 2021, Wertz et al., 2019; Plomin et al., 1977). Estimating gene-environment interactions in the presence of rGE can lead to false positive results (Keller, 2014). However, most previous studies employ a *between-family analysis* (Papageorge & Thom, 2020), which does not deal with rGE or unmeasured environmental factors across families (Fletcher & Conley, 2013). To address rGE, controlling for parental genotypes is a possible solution (Isungset et al., 2021, Breinholt and Conley, 2023). In the case of the *within-family design,* when sibling and genetic data are available (Fletcher et al., 2023), family fixed-effects models can exploit random segregation of alleles between siblings while controlling for all usually unmeasured (genetic and environmental) family circumstances shared within the household (Cheesman et al., 2022, Cheesman et al., 2022, Domingue et al., 2015). Still, there is evidence that passive rGE biases PGI coefficients in the within-family design (Trejo & Domingue, 2018). A *trio design* can be implemented under the rare but increasing availability of parental genetic information (Breinholt & Conley, 2023) to directly control for mothers’ and fathers’ genotypes and exploit random variation in non-transmitted alleles. In doing so, one can get a more robust estimation of G × E interactions since controlling for parents’ PGIs makes it credible to assume that a child’s PGI is exogenous to family characteristics (Isungset et al., 2021). Thus, in this article, we triangulate findings from these between-, within-family and trio research designs to shed light on different sources of variation and confounding to identify more robust G × E interactions (Demange et al., 2022).

Second, we study cognitive and noncognitive skills PGIs. Previous G × E interaction studies on educational outcomes mainly focus on the genetic propensity for adult attainment, using the PGI for total years of education (Lee et al., 2018). However, educational attainment can be further distinguished into cognitive and noncognitive skills (Demange et al., 2021), which are among the most predictive and closest traits in the causal chain explaining educational performance (McGue et al., 2020, Borghans et al., 2016). While cognitive skills are measured with validated intelligence and cognitive performance tests (Nisbett et al., 2012), noncognitive skills are a less-well-defined concept (Demange et al., 2021), including a wide range of traits generally improving one’s educational performance (Jackson & Moullin, 2023), such as grit, conscientiousness, motivation, or social skills (Kevenaar et al., 2023, Smithers et al., 2018). As cognitive and noncognitive skills are two distinct latent constructs that can act as complements or substitutes in learning and educational performance (Light & Nencka, 2019), family SES might moderate (e.g., compensate or enhance) their (genetic) association with educational outcomes differently (Damian et al., 2015; Gil-Hernández, 2021; Holtmann et al., 2021). The first G × E study using a PGI for noncognitive skills found no interaction with parental SES in explaining academic achievement from ages 7 to 16 in the United Kingdom (Malanchini et al., 2023). Building on this study, we use PGIs for cognitive and noncognitive skills to untangle the genetic architecture of the main predictors of educational attainment and estimate G × E interactions over further educational outcomes, triangulating from different research designs.

Third, we investigate different educational outcomes. Previous studies independently covered several educational outcomes such as test scores in late primary and lower-secondary school (Cheesman et al., 2022, Cheesman et al., 2022, Isungset et al., 2021, Malanchini et al., 2023), high-school outcomes (e.g., persistence in mathematics; Harden et al., 2020), or college completion (Papageorge & Thom, 2020). Yet, adult educational attainment results from successive teacher assessments and transitions over the educational system with different selectivity and implications for social demotion (Blossfeld et al., 2016). For instance, Ghirardi and Bernardi (2023) hypothesised and evidenced that the interaction between PGIs and parental SES might depend on the selectivity of the educational outcomes investigated. Thus, we take a life-course approach (Blossfeld et al., 2019) investigating seven educational outcomes from childhood to adulthood: grades in mathematics and reading (age 7–10), high-stakes standardised test scores (CITO: age 12), school track in secondary school (age 12–18), and adult educational attainment (age ≥ 25). This life-course approach might shed further light on G × E interactions and mechanisms, while snapshots or single educational outcomes might give a fuzzy picture of potential G × E interactions.

In this study, we test two competing hypotheses on G × E interactions in educational outcomes, the compensatory and Scarr-Rowe hypotheses, asking the following research question: Does the effect of genetic propensity for cognitive and noncognitive skills on educational outcomes matter more for high- or low-SES children? We answer this question through a pre-registered research design and a genotyped panel of twins, siblings, and parents from the *Netherlands Twin Register* (NTR) (Ligthart et al., 2019). We use PGIs for cognitive and noncognitive skills to predict seven educational outcomes across three research designs, namely the between-family, within-family, and trio designs, conducting 42 distinguished analyses (i.e., 2 PGIs × 7 outcomes × 3 designs). While most previous research focused on comprehensive educational systems from the United States (US), UK, or Norway, we test the Scarr-Rowe and compensatory hypotheses in the context of the modern Dutch early tracked educational system, which is highly selective and horizontally stratified (Blossfeld et al., 2016).

The following sections outline our two hypotheses’ theoretical framework, explain the data, methods and variables, and discuss our findings’ implications for the interdisciplinary social stratification and sociogenomics literature.

## 2 Theoretical framework

The existing literature shows that genes and environments correlate and interact in a complex interplay to influence individuals’ educational attainment (Papageorge and Thom, 2020, Belsky et al., 2018, Schmitz and Conley, 2017). This section discusses the main theories from behavioural genetics and social stratification accounting for the interaction between a family SES and genetic propensity for education.

### 2.1 The Scarr–Rowe hypothesis

The *Scarr–Rowe hypothesis* claims that the relative importance of genetics for cognitive ability is higher in socioeconomically advantaged families than in disadvantaged families (Rowe et al., 1999; Scarr-Salapatek, 1971). The underlying assumption of this interaction effect, in which genetic variation is suppressed in low-SES families, is that those children reared in deprived environments, generally characterised by material scarcity, chronic stress and low levels of cognitive stimulation (Mcewen & Mcewen, 2017), cannot fully express their genetic potential (Uchikoshi and Conley, 2021, Baier and Van Winkle, 2021). Contrastingly, children from advantaged families experience an enriched rearing environment where their genetic potential is fully expressed.

The Scarr-Rowe hypothesis was initially developed in studies about intelligence, using classical twin models of variance decomposition and finding support in the specific context of the US, a country characterised by high child poverty, meagre social policies and high-income inequality (Turkheimer & Horn, 2014), while among Western European countries and Australia, this hypothesis was not replicated (Tucker-Drob & Bates, 2016). Some authors argue that the genetic heritability of intelligence might not follow a linear pattern, being only suppressed in highly deprived environments to plateau after reaching a minimum environmental quality threshold (Nielsen, 2016, Pennington et al., 2009).

This hypothesis was extended by looking at other educational outcomes beyond intelligence (Baier & Lang, 2019) and using molecular data—directly measuring the genome. Studies using molecular data have investigated this hypothesis across various educational outcomes such as school test scores (Isungset et al., 2021), school tracking (Uchikoshi and Conley, 2021, Harden et al., 2020), educational attainment (Lin, 2020), and years of education (Conley et al., 2015). Moreover, instead of examining the moderation of genetic expression by family SES, some studies further examine the moderating role of environments like schools (Trejo et al., 2018), teachers (Arold et al., 2022), or neighbourhoods (Cheesman et al., 2022, Cheesman et al., 2022). However, the evidence regarding the Scarr-Rowe hypothesis is highly inconclusive, as reviewed in Table A1 in the Appendix.

**Fig. 1:**
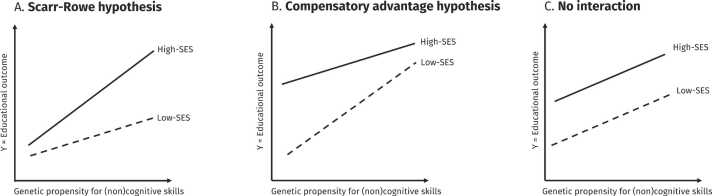
Hypotheses for the interaction between family SES and genetic propensity for cognitive and noncognitive skills on educational outcomes.

Fig. 1 Panel A illustrates an application of the Scarr-Rowe hypothesis for the relationship between genetic predisposition for cognitive and noncognitive skills and educational outcomes and its moderation by family SES. The line representing individuals with low SES is flatter, indicating that individuals with a high genetic propensity for cognitive and noncognitive skills do not realise their full genetic potential in disadvantaged socioeconomic environments. According to the Scarr-Rowe hypothesis, we expect to observe in our study that:

H1 *PGIs for cognitive and noncognitive skills are more predictive of educational outcomes for children with high-SES parents than low-SES parents.*

### 2.2 The compensatory advantage hypothesis

In social stratification literature, the *compensatory advantage hypothesis* posits heterogeneity by parental SES in the penalty of a negative trait or event for status attainment at $t_{0}$ on a subsequent outcome at $t_{+1}$. According to this hypothesis and related findings, the educational and labour market trajectories of individuals from privileged backgrounds are less influenced by previous adverse events or traits (Bernardi and Gil-Hernández, 2021, Bernardi and Grätz, 2015, Bernardi, 2014), such as a low genetic propensity for education. This hypothesis was initially developed to examine educational inequalities, proposing and showing that children from advantaged social backgrounds are more likely to overcome the negative consequences of prior poor academic performance (e.g., bad grades, grade repetition) by progressing towards higher education (Bernardi and Triventi, 2018, Bernardi, 2012).

Thanks to their cultural and economic resources, high-SES families might (un)consciously *nurture* their low-endowed children’s *nature* with compensatory investments^3^ (Breinholt and Conley, 2023, Houmark et al., 2020). Thus, high-SES families would compensate for their children’s unfavourable (genetic) traits to eventually develop higher—than usually predicted by low genetic potential—cognitive and noncognitive skills, academic performance (e.g., grades and test scores) or transition rates to academic tracks leading to college so to avoid downward educational mobility. In line with this hypothesis, some recent molecular genetics studies found a negative interaction between enriched environments and educational outcomes, showing the compensatory or substitutive role of high-SES families, neighbourhoods and schools, or high-quality teachers to lift students with low genetic endowments for education (Arold et al., 2022, Cheesman et al., 2022, Cheesman et al., 2022, Harden et al., 2020, Lin, 2020). However, the evidence for the compensatory hypothesis is as mixed as with the Scarr-Rowe hypothesis, as reviewed in the introduction and Table A1 in the Appendix.

Specifically, the penalty in later educational outcomes associated with an initial negative trait or event is expected to be smaller for high-SES individuals due to class-based differences in expectations and resources. The compensatory advantage hypothesis is based on the *Relative Risk Aversion* model formalising SES inequalities in educational transitions (Breen & Goldthorpe, 1997). This model draws from *prospect theory* to propose that individuals are more sensitive to losses than gains due to evolutionary pressures so that, given their relative position in the class structure, high-SES subjects are more driven to avoid downward social mobility than low-SES subjects to move upward. For high-SES individuals, a previous adverse event or trait, such as low academic potential or performance, does not necessarily impact their later educational and occupational attainment because they stick to high educational expectations to maintain their privileged status (Bernardi & Valdés, 2021). In contrast, low-SES students would be more sensitive to a previous adverse event (Holm et al., 2019), trait or contextual (i.e., macroeconomic) climate in their educational careers (Salazar & Radl, 2020), as relatively lower educational attainment would suffice to maintain or improve their status. Compared to disadvantaged parents, advantaged families have a large pool of economic, cultural and social resources (Lareau, 2015) to compensate for early adverse events through, for instance, private tutoring and schools, alternative educational pathways, or school involvement to influence teacher’s track recommendations.

Fig. 1 Panel B displays the compensatory advantage hypothesis applied to the association between genetic propensity for cognitive and noncognitive skills and educational outcomes and its heterogeneity by family SES. As can be seen, the slope is flatter for high-SES compared to low-SES children. According to this hypothesis, we expect to observe in our study that:

H2 *PGIs for cognitive and noncognitive skills are less predictive of educational outcomes for children with high-SES parents than low-SES parents.*

Finally, Fig. 1 Panel C represents the null hypothesis of no interaction G × E interaction that would reject both the compensatory advantage and Scarr-Rowe hypotheses, where the genetic propensity for cognitive and noncognitive skills is equally predictive for high- and low-SES students with parallel slopes.

## 3 The context of the Dutch educational system

The modern Dutch educational system is primarily public and, despite some minor changes, relies on the 1968 *Mammoth Act*. This legal framework established free-of-charge compulsory education until the age of 16—if a degree is obtained or until 18 if no degree yet, and early tracking into different school tracks from the primary-to-secondary transition at age 12 (grade 6) (Luijkx & de Heus, 2008).

Primary education is compulsory from age 5 until age 12, but almost all children are enrolled from age 4, with a total duration of 8 grades (2 kindergarten and 6 elementary education grades). In secondary education, the system comprises three^4^ main educational tracks in lower-secondary education from age 12 and grade 7 (Luijkx & de Heus, 2008), including (1) 4-year pre-vocational tracks (VMBO), further divided into four sub-tracks with different degrees of practical or theoretical focus; (2) a 5-year senior general education track (HAVO); (3) and a 6-year pre-university track (VWO).

The tracking process occurs at the end of primary school. It mainly depends on a combination of the student’s high-stakes standardised CITO test scores (*Dutch National Institute for Educational Measurement*), measuring arithmetic and language competencies, and the teacher’s recommendations based on a student’s GPA and behaviour. The CITO test is administered in most Dutch schools, with scores ranging from 501 to 550, with a grade above 535 generally granting a recommendation to the two upper secondary tracks (HAVO and VWO). The CITO scores and track recommendations are strongly associated with final track enrolment.

Students enrolled in the pre-vocational tracks in lower secondary education most often transit into a vocational school (MBO) in upper secondary education, lasting between 1 and 4 years, depending on the specialisation. Access to higher education is granted either through a degree from the HAVO track, which provides access to universities of applied sciences (HBO), the most widespread option, or the completion of the VWO track leading to college or research-oriented universities (WO). However, achieving a level-4 MBO degree gives access to a university of applied sciences. At the end of the second phase of HAVO (years 4–5) and VWO (years 4–6), to access higher education, students must pass a standardised central exam for most of their subjects in combination with school-administered and designed tests for each subject to get a certificate and assign the final graduation grade.

Due to these institutional arrangements, parents of low-performing children may have less room to influence or bypass track decisions (Blossfeld et al., 2016). Thus, the Dutch educational system of early tracking is a stringent test for the *compensatory advantage hypothesis*. However, there is still room for high-SES families with high educational expectations to deploy compensatory strategies if their children struggle at school or underperform to prevent them from downward mobility and enrol them into upper secondary tracks (Dronkers & Korthals, 2016). Among the possible mechanisms at play, high-SES parents might, for instance, help their kids with homework or pay for private tutoring to improve their performance in the CITO test, pressure teachers to get a higher recommendation (Timmermans et al., 2018), proactively search for alternative schools, or in case of a negative recommendation, push their children for upper-track mobility.

## 4 Data, samples and variables

Before accessing the data and analyses, this study’s hypotheses and research design were pre-registered on the *Open Science Framework repository*. Additional non-preregistered analyses indicated below should be considered exploratory. Further deviations from the preregistration are detailed in Section 2 of the Appendix. All the statistical code necessary for replication is available on a *GitHub repository*. We cannot publish the NTR data, as participants did not consent to sharing their data publicly.

### 4.1 Data

The unique data of the *Netherlands Twin Register*^5^ (NTR) (Ligthart et al., 2019) contains multiomics, phenotypic, and sociodemographic information about monozygotic (MZ) and dizygotic (DZ) twins (identical and opposite sex) and their family members, such as parents and siblings. The NTR’s collection of blood and saliva DNA samples to identify single nucleotide polymorphisms (SNPs) allows us to construct PGIs for cognitive and noncognitive skills building on genome-wide association studies (GWAS) with large discovery samples of genetically unrelated individuals (Lee et al., 2018).

The NTR comprises two large sub-groups: families with young and adolescent twins (the *Young Netherlands Twin Register*) and adult twins (the *Adult Netherlands Twin Register*). First, the *Young Netherlands Twin Register* (YNTR) recruited families with young and adolescent twins with a cross-sequential design based on the birth cohort (1986–2019) and age (1, 2, 3, 5, 6/7, 9/10, 12, 14, 16, 18) with surveys administered from 1988 to 2020. The YNTR draws cross-sectional and longitudinal information from parental reports of children aged 1 to 12, children’s self-reports from age 14 to 18, and twins’ full siblings’ self-reports from age 14. Second, the *Adult Netherlands Twin Register* (ANTR) comprises adult twins aged 18 or older born between 1909 and 2001 (mean = 1990, SD = 17) and their family members with 2- to 3-yearly surveys on health, lifestyle, and personality from 1991 to 2020 (Willemsen et al., 2013). Once they become adults, the YNTR twins and other individuals can join the ANTR, so a small subset (5 %) of the YNTR is followed up from age 1 to adulthood. Here, we use both these datasets. YNTR is used as an unbalanced panel (e.g., follow-ups and new entrants in each survey) to study early educational outcomes from age 7 to 18., such as school grades, test scores (CITO) and secondary school track. Furthermore, we use the ANTR to analyse adult educational attainment from age 25 (mean birth year = 1972, SD = 13). To make the birth year distribution more overlapping with the YNTR analytical samples (mean = 1995, SD = 7), we restrict the ANTR sample to individuals born from 1980 (mean birth year = 1987, SD = 3) in the main analysis, while in the Appendix (Section 7.3.3) we run additional analyses for those born before 1980.

As illustrated in Appendix’s Table A2, the YNTR covers 70 % (n = 68,771) of all Dutch twin pairs born between 1986 and 2019 (N = 98,877), based on official statistics (Statistics Netherlands, 2024c), while older adult cohorts born before 1980 are less well represented (Ligthart et al., 2019). Despite its ample population coverage, as with most twin registers, the NTR slightly overrepresents high-SES parents and high-achievers^6^ compared to the general Dutch population, especially when filtering out those cases with available genetic information. As shown in Appendix Table A2, about 41 % (36 %) of the fathers and 35 % (29 %) of the mothers have higher education in our genotyped analytical (full) samples (mean survey year = 2003). At the same time, in the general equivalent population (individuals aged 25–55 in 2003) to the YNTR birth cohorts, these figures reach 30 % for men and 26 % for women (Statistics Netherlands, 2024a). The average mother’s age at first birth in the NTR data (mean = 33) is also slightly higher than the total population in the equivalent period between 1986 and 2019, with a mean of 28.9 (Statistics Netherlands, 2024c). The YNTR students took the CITO standardised test in 2005, on average, with a mean score of 538.9 (SD = 9) versus a 536 average in the academic year 2005/2006, according to population data (Statistics Netherlands, 2024b). Finally, children with a migration background are underrepresented due to the need to control for population stratification in genetic analysis, excluding ethnic outliers. Unluckily, the NTR does not provide sampling weights to adjust for participation rates and population characteristics, which should be considered when interpreting findings.

### 4.2 Samples

Table A3 in the Appendix documents the selection procedure from the full to the analytical samples, including the sample sizes and share of missing values by variable. Most missing cases are due to genotype information only being available in about 25 % of the observations, resulting in a slightly positive selection of the analytical compared to the full samples, broadly representative of the corresponding population. The YNTR full samples range from 29,274 to 21,331 cases among the survey waves where the outcome variables are mainly reported (ages 7–12). At the same time, the ANTR dataset reaches 61,350 observations before variable selection.

We analyse all twins and their siblings with available information for the between-family analysis (see Sections 5.1. and 5.3. below). However, we exclude one MZ co-twin randomly^7^ in families with a pair of MZ-twins to avoid the lack of variation in the genetic characteristics (Mills et al., 2018), as the MZ-twins genetic correlation is ≈ 1. For the trio analysis, we follow the same criteria as the between-family analysis, except we limit the analysis to children with no missing information on parents’ genotypes. In the within-family analysis (see Section 5.2.), we keep a balanced sample of two family members by family to implement family fixed effects (FE) models by comparing (1) two DZ-twins in families where we observe the pair; (2) if one DZ co-twin is missing and there is only one full sibling observed within the family, we compare them and, if more than one sibling is observed, we pick a random sibling; (3) if two MZ-twins are observed in the same family, to avoid no within-family variation, we randomly selected one MZ-twin by family and assigned it a sibling if only one is observed, or a random one if more than one is available.

After applying the selection criteria, excluding non-European ancestry individuals (13.1 % of the sample with available PGIs) due to population stratification issues with PGIs, and deleting missing values in our independent variables of interest (PGIs, and parental education in all designs, and parental PGIs in the trio design), the analytical samples range from 426 to 3875 observations (see Table 1) depending on the research design (see Section 5) and outcome analysed (see Section 4.3.1). As the NTR follows a cross-sequential design that does not require participation across all surveys, information on one or more analysed variables might be missing. We use different analytical samples by research design and outcomes to maximise sample size and power.^8^ In addition, adult educational attainment (from age 25) is the only outcome from the ANTR cohort, with available individuals born on average in 1972, while for the remaining outcomes taken from the YNTR, participants were born on average between 1994 and 1996. Thus, to select a more homogenous sample in terms of its historical context, we restrict the primary analysis sample to those born from 1980 for adult educational attainment within the ANTR cohort. The distribution of the main variables of interest is consistent across subsamples by outcome and research design.

**Table 1:** Analytical sample by research designs and outcomes.

		Research design		
Age	Outcome	Between	Within	Trio
7	Mathematics grade	3728	2124	1861
7	Reading grade	3756	2130	1869
10	Mathematics grade	3829	2212	2022
10	Reading grade	3875	2236	2051
12	Test scores (CITO)	2690	1500	1254
12–18	Upper secondary track	3318	2004	1500
≥ 25	Educational attainment (≥ 1980)	1224	426	576

### 4.3 Variables

#### 4.3.1 Dependent variables

We study seven educational outcomes^9^ measured at different time points in the life course as dependent variables. First, we use school grades in mathematics and reading in primary education (at ages 7 and 10) measured on a 1-to-5 scale (1 = poor; 2 = weak; 3 = fair; 4 = good; 5 = very good) as reported by mothers.^10^ Second, we look at standardised national test scores (CITO) at age 12, a crucial outcome that influences teacher’s track recommendation and student’s track choice in lower-secondary education, ranging from 501 to 550 in its original scale, which we transformed into z-scores. Third, we take information about the type of secondary school attended using the last available information from age 12 to 18.^11^ We coded as 1 those children who attend university preparation education (VWO) or senior general secondary education (HAVO) and as 0 those who attended pre-vocational secondary education or upper-secondary vocational education (MBO). Finally, we look at adult educational attainment from age ≥ 25 by distinguishing between those who have a university (wo) or university of applied sciences (HBO) degree (1) and those who finished secondary or primary education (0).

#### 4.3.2 Independent and moderator variables

As the main independent variables, we use mean PGIs for cognitive and noncognitive skills. A PGI is a quantitative variable summarising the individual’s genetic propensity for certain traits or behaviours (Mills & Tropf, 2020).^12^ PGIs for cognitive and noncognitive skills are based on the GWAS effect sizes estimated by Demange et al. (2022) and the GWAS-by-subtraction method developed by Demange et al. (2021).^13^ This method leverages genomic structural equation modelling to create latent genetic constructs from existing summary statistics. A noncognitive construct was created by “subtracting” the genetic variance of cognitive skills from the genetic variance of educational attainment. The PGIs we built from these GWAS, therefore, reflect the genetic variance of cognitive performance (cognitive skills PGI) and the genetic variance of educational attainment independent of cognitive performance (noncognitive skills PGI), as tagged by common genetic variations (SNPs). PGIs were computed using Plink software version 1.9 based on weighted betas obtained using the LDpred v1.0.0 software, using infinitesimal prior, a linkage disequilibrium (LD) pruning window of 250 kb and 1000Genomes phase 3 CEU population as LD reference. In the primary analysis, we use the PGI for cognitive and noncognitive skills as a continuous variable transformed into z-scores. We also categorise them in terciles in the robustness checks (see Sections 7.2.2. and 7.2.3. in the Appendix) to account for potential nonlinearities.

As the main measure of a family’s SES, we use a dichotomous variable distinguishing between high- and low-medium-educated parents (1 = university; 0 = primary, secondary education, and higher vocational schooling), taking this information in the first year available (e.g., at age 3 for YNTR participants). Using a dominance approach, we look at the highest educational level among fathers and mothers. As a robustness check, we replicate the analyses using the parental highest occupation (1 = higher-grade professionals; 0 = rest) as an alternative measure of a family’s SES, and we find robust results (see Section 7.4 in the Appendix).

#### 4.3.3 Control variables

In all the designs, we control for ancestry’s first 10 genetic principal components (PCs) and the genotyping platform, a standard practice in the genomics literature (see Okbay et al., 2016). The trio analysis includes controls for the mother’s and father’s PGIs for cognitive and noncognitive skills, constructed following the same procedure used to operationalise the children’s PGIs (see Section 4.3.2. above). As a robustness check, we replicate all the main analyses controlling for gender and birth year to account for potential biases in the original GWASs on which the PGS weights are based. Still, the results align with the main models (see Tables A20–22 in Section 7.1. of the Appendix).

## 5 Methods

We conduct three different designs, between-, within-family and trio, to account for different sources of confounding and bias. In all three designs, we run linear probability models (LPM) for tracking and adult educational attainment, dummy outcomes, and ordinary least squares (OLS) regressions for school grades and CITO outcomes, with standard errors adjusted by family cluster to account for the non-independence of observations.

We follow two strategies to ensure our results are robust when estimating G × E interactions. First, we add interaction terms between PGIs, parental education and all control variables outlined above to properly account for potential confounding between covariates and the interaction term (Keller, 2014). Second, we apply formal corrections to the p-values of our estimations to account for multiple comparisons. In particular, we use Bonferroni and Romano-Wolf corrections to exclude the possibility that our results are false positives by chance from multiple hypothesis testing. According to Bonferroni’s conservative correction for 7 outcomes, interactions must have a p-value below the adjusted threshold of 0.007 (0.05/7 for each PGI) to be considered statistically significant. Romano-Wolf’s (2005) correction is less stringent following a bootstrap procedure (Clarke et al., 2020, Romano and Wolf, 2016). Section 6 in the Appendix details these multiple testing methods and presents the results adjusted from both approaches.

Moreover, as robustness checks, we replicate the analysis in all three designs using alternative model’s specifications (e.g., logistic regression of our dichotomous outcomes: school tracking and adult educational attainment), samples and control variables, and operationalising PGIs in terciles for all outcomes to account for potential nonlinearities in an attempt to prevent bias and false discovery in our findings (Domingue et al., 2022). As shown in the Appendix in Section 7, these robustness analyses back the article’s main findings.

### 5.1 Between-family analysis

We estimate Eq. (1a) where *i* is a child, and $Z_{i}$ is a vector of controls (10 genetic principal components and genotyping platform). Specifically, to assess the predictive power of children’s PGI for cognitive and noncognitive skills, in baseline Eq. (1a), we include the PGI for cognitive skills, PGI for noncognitive skills and family’s SES. We expect β1, β2 and β3 to be positive and statistically significant in Eq. (1a).

(1a) $$Y_{i}=\alpha +{\beta_{1}{\mathrm{PGI Cog}}_{i}+\beta_{2}{\mathrm{PGI NonCog}}_{i}+\beta }_{3}{\mathrm{SES}}_{i}+Z_{i}+\varepsilon_{i}$$

The below equations formalise the two-way interactions between a child’s PGI for cognitive (1b) or noncognitive skills (1c) and parental SES. According to the *Scarr-Rowe hypothesis* (H1), we expect β4 ((1b), (1c)) to be positive and significant: PGIs for cognitive or noncognitive skills are more predictive among high-SES children. Alternatively, according to the *compensatory advantage hypothesis* (H2), β4 is expected to be negative and statistically significant: PGIs for cognitive or noncognitive skills are less predictive for high-SES children’s educational outcomes than low-SES children.

(1b) $$Y_{i}=\alpha +{\beta_{1}{\mathrm{PGI Cog}}_{i}+\beta_{2}{\mathrm{PGI NonCog}}_{i}+\beta }_{3}{\mathrm{SES}}_{i}+Z_{i}+\beta_{4}{\mathrm{PGI Cog}}_{i}\times {\mathrm{SES}}_{i}+\beta_{5}{\mathrm{PGI Cog}}_{i}\times {\mathrm{PGI NonCog}}_{i}+\beta_{6}{\mathrm{PGI Cog}}_{i}\times Z_{i}+\beta_{7}{\mathrm{SES}}_{i}\times {\mathrm{PGI NonCog}}_{i}+\beta_{8}{\mathrm{SES}}_{i}\times Z_{i}+\varepsilon_{i}$$

(1c) $$Y_{i}=\alpha +{\beta_{1}{\mathrm{PGI Cog}}_{i}+\beta_{2}{\mathrm{PGI NonCog}}_{i}+\beta }_{3}{\mathrm{SES}}_{i}+Z_{i}+\beta_{4}{\mathrm{PGI NonCog}}_{i}\times {\mathrm{SES}}_{i}+\beta_{5}{\mathrm{PGI NonCog}}_{i}\times {\mathrm{PGI Cog}}_{i}+\beta_{6}{\mathrm{PGI NonCog}}_{i}\times Z_{i}+\beta_{7}{\mathrm{SES}}_{i}\times {\mathrm{PGI Cog}}_{i}+\beta_{8}{\mathrm{SES}}_{i}\times Z_{i}+\varepsilon_{i}$$

### 5.2 Within-family design

We implement an additional research design to account for potential bias due to rGE and exploit random variation in siblings’ genetic endowments. We apply the within-family design with twin and sibling FE. Using this design, we can assume that variation in siblings’ PGIs for cognitive and noncognitive skills is exogenous (direct genetic effects) (Demange et al., 2022) since each sibling randomly gets 50 % of their genetic makeup in the process of reproduction, being unconfounded by parental SES.

**Fig. 2:**
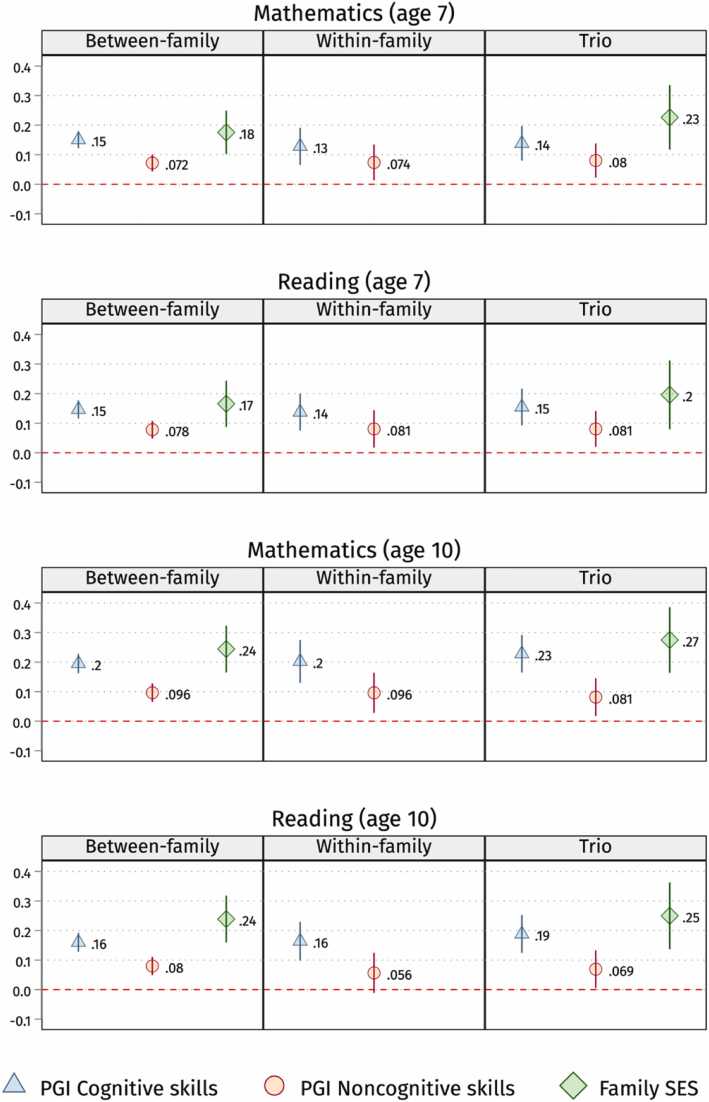
Children’s cognitive and noncognitive PGIs, and family SES (inestimable in the within-family analysis) coefficients on school grades in the three designs (between, within and trio). Note: 95 % confidence intervals. Standard errors clustered by families. We run OLS (for between-family and trio analysis) and family FE (for within-family analysis) models with controls. See sample sizes in Table 1. PGIs and CITO scores are z-standardised. See the Appendix for full output in Tables A4–A5, A7, and A9–A11. SES is fixed in the within-family models, so not shown in this figure. In black are the coefficients that are not statistically significant anymore after correcting for Bonferroni multiple testing (p-value < 0.007).

**Fig. 3:**
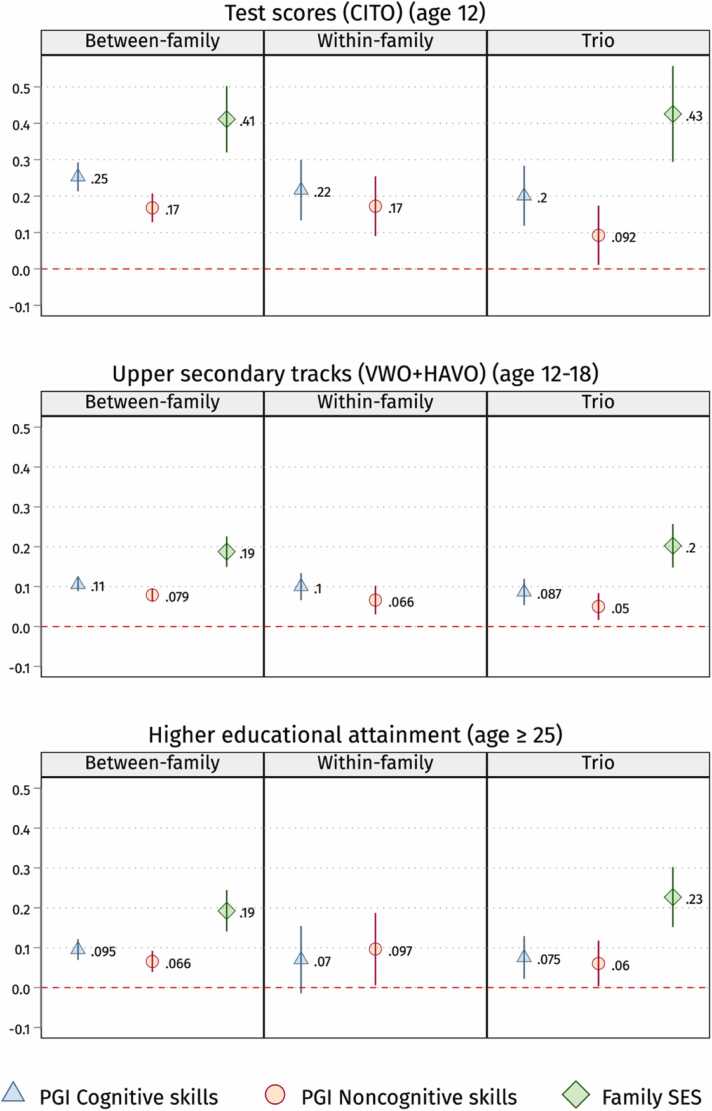
Children’s cognitive and noncognitive PGIs, and family SES (inestimable in the within-family analysis) coefficients on CITO test scores, upper secondary tracks attendance, and higher educational attainment in the three designs (between-family, within-family and trio analysis). Note: 95 % confidence intervals. Standard errors clustered by families. We run OLS or LPM (for between-family and trio analysis) and family FE (for within-family analysis) models with controls. See sample sizes in Table 1. PGIs and CITO scores are z-standardised. See the Appendix for full output in Tables A4–A5, A7, and A9–A11. SES is fixed in the within-family models, so it is not shown in this figure.

In Eq. (2a), i is a child within family *j*, $\delta_{j}$ represents the family-FE and $Z_{\mathrm{ij}}$ is a vector of controls including 10 principal genetic components and the genetic testing platform. We run family FE models, including children’s PGIs for cognitive and noncognitive skills and controls in the main model in Eq. (2a) above. Again, in line with previous between-family models, we expect β1 and β2 to be positive and statistically significant in Eq. (2a). However, effect sizes might be slightly smaller due to attenuation bias, partial control for gene-environment correlations and other family-constant factors. Since parental SES is the same for twins and siblings within the same family, its constitutive term can not be estimated by design and, hence, is absent in Figs. 2 and 3.

(2a) $$Y_{\mathrm{ij}}=\alpha +\beta_{1}{\mathrm{PGI Cog}}_{\mathrm{ij}}+\beta_{2}{\mathrm{PGI NonCog}}_{\mathrm{ij}}+Z_{\mathrm{ij}}+\delta_{j}+\varepsilon_{\mathrm{ij}}$$

**Fig. 4:**
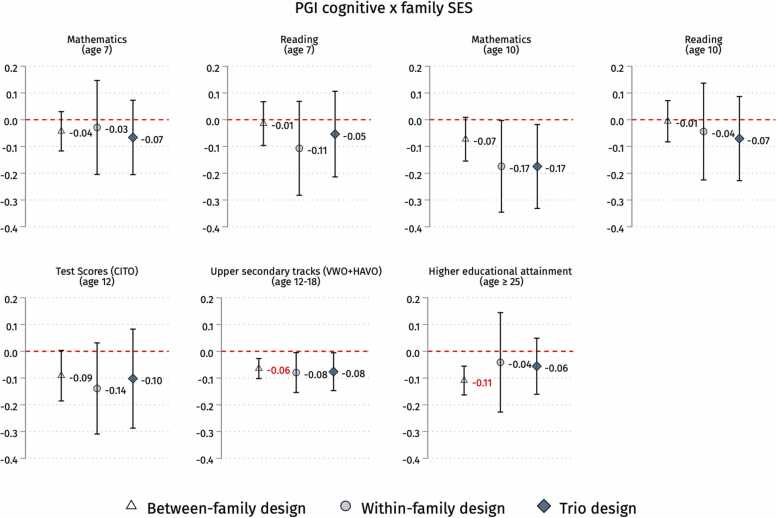
Interaction coefficients between PGI for cognitive skills and family’s SES on educational outcomes in the three designs (between-family, within-family and trio analysis). Note: 95 % confidence intervals. Standard errors clustered by families. We run OLS or LPM (for between and trio analysis) and family FE (for within-family analysis) models with controls. Following Keller (2014), gene-covariates (cognitive skills PGI) and environment-covariates (family’s SES) interactions are included. See sample sizes in Table 1. PGIs and CITO scores are z-standardised. See Tables A6, A8 and A12 in the Appendix for full output. In red are the statistically significant coefficients after correcting for Bonferroni multiple testing (see Tables A16 and A17 in the Appendix).

**Fig. 5:**
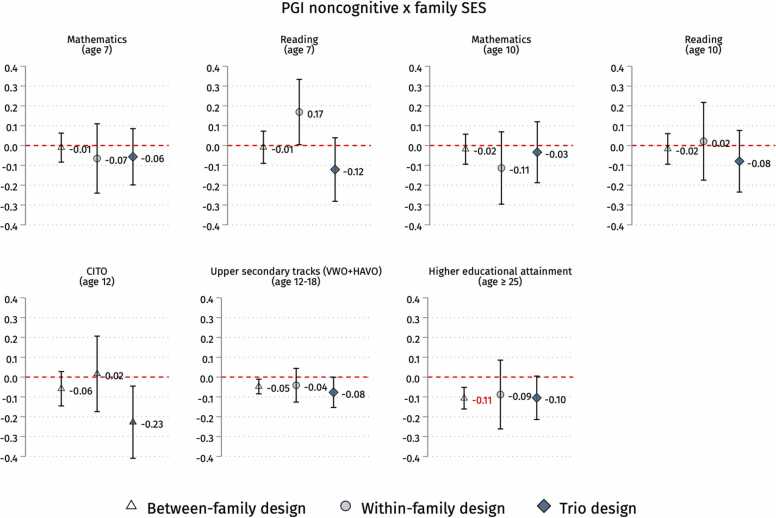
Interaction coefficients between PGI for noncognitive skills and family’s SES on educational outcomes in the three designs (between-family, within-family and trio analysis). Note: 95 % confidence intervals. Standard errors clustered by families. We run OLS or LPM (for between-family and trio analysis) and family FE (for within-family analysis) models with controls. Following Keller (2014), gene-covariates (noncognitive skills’ PGI) and environment-covariates (family’s SES) interactions are included. See sample sizes in Table 1. PGIs and CITO scores are z-standardised. See Tables A6, A8 and A12 in the Appendix for full output. In red are the statistically significant coefficients after correcting for Bonferroni multiple testing (see Tables A16 and A17 in the Appendix).

Finally, we run heterogeneous family FE models as in Eq. (2a) by parental SES in Eq. (2b) (low SES) and (2c) (High SES) (Giesselmann & Schmidt-Catran, 2020). We expect the coefficient β1 on cognitive skills PGI and β2 on noncognitive skills PGI to be smaller for low SES in Eq. (2b) than for high SES in Eq. (2c) under the *Scar-Rowe hypothesis* (H1) or larger under the *compensatory advantage hypothesis* (H2). In Fig. 4, Fig. 5, we display the coefficient difference for β1 and β2 between high and low-SES groups, which would be of positive sign according to H1 or negative under H2.

(2b) $$Y_{\mathrm{ij}}=\alpha +\beta_{1}{\mathrm{PGI Cog}}_{\mathrm{ij}}+\beta_{2}{\mathrm{PGI NonCog}}_{\mathrm{ij}}+Z_{\mathrm{ij}}+\delta_{j}+\varepsilon_{\mathrm{ij}}|{\mathrm{SES}}_{i}=\mathrm{Low}(0)$$

(2c) $$Y_{ij}=\alpha +\beta_{1}{\mathrm{PGI Cog}}_{\mathrm{ij}}+\beta_{2}{\mathrm{PGI NonCog}}_{\mathrm{ij}}+Z_{\mathrm{ij}}+\delta_{j}+\varepsilon_{\mathrm{ij}}|{\mathrm{SES}}_{i}=\mathrm{High}(1)$$

### 5.3 Trio design

We restrict the samples to children with available parental genetic information to perform the trio design. We estimate the models above in the between-family analysis and additionally control for parents’ and mothers’ PGIs for cognitive and noncognitive skills to control for rGE.

Firstly, we estimate the model expressed in Eq. (3a) where *i* is child, and $Z_{i}$ is a vector of controls (10 genetic principal components and genetic testing platform). Then, in Eq. (3b), we add the father’s and mother’s PGIs for cognitive and noncognitive skills to account for rGE. The coefficients β1 and β2 should remain positive and statistically significant in the presence of direct genetic effects of children’s PGI for cognitive and noncognitive skills on educational outcomes. Besides, the coefficient β3 on parental SES should remain positive and significant net of intergenerational genetic transmission.

(3a) $$Y_{i}=\alpha +{\beta_{1}{\mathrm{PGI Cog}}_{i}+\beta_{2}{\mathrm{PGI NonCog}}_{i}+\beta }_{3}{\mathrm{SES}}_{i}+Z_{i}+\varepsilon_{i}$$

(3b) $$Y_{i}=\alpha +{\beta_{1}{\mathrm{PGI Cog}}_{i}+\beta_{2}{\mathrm{PGI NonCog}}_{i}+\beta }_{3}{\mathrm{SES}}_{i}+\beta_{4}{\mathrm{PGI Cog}}_{i}{\mathrm{Father}+\beta }_{5}{\mathrm{PGI Cog}}_{i}\mathrm{Mother}+\beta_{6}{\mathrm{PGI NonCog}}_{i}\mathrm{Father}+\beta_{7}{\mathrm{PGI NonCog}}_{i}\mathrm{Mother}+Z_{i}+\varepsilon_{i}$$

Finally, in the trio design, we look at the two-way interactions between a child’s PGI for cognitive (Eq. (3c)) or noncognitive skills (Eq. (3d)) and parental SES. As for the between- and within-family designs described above, we expect the interaction between parental SES and children’s PGI in β8 to be positive and statistically significant under the Scarr-Rowe hypothesis (H1), and negative and statistically significant under the compensatory advantage hypothesis (H2).

(3c) $$Y_{i}=\alpha +{\beta_{1}{\mathrm{PGI Cog}}_{i}+\beta_{2}{\mathrm{PGI NonCog}}_{i}+\beta }_{3}{\mathrm{SES}}_{i}{+\beta }_{4}{\mathrm{PGI Cog}}_{i}{\mathrm{Father}+\beta }_{5}{\mathrm{PGI Cog}}_{i}\mathrm{Mother}+\beta_{6}{\mathrm{PGI NonCog}}_{i}\mathrm{Father}+\beta_{7}{\mathrm{PGI NonCog}}_{i}\mathrm{Mother}+Z_{i}+\beta_{8}{\mathrm{PGI Cog}}_{i}\times {\mathrm{SES}}_{i}+{\beta_{9}{\mathrm{PGI Cog}}_{i}\times {\mathrm{PGI NonCog}}_{i}+\beta }_{10}{\mathrm{PGI Cog}}_{i}\times {\mathrm{PGI Cog}}_{i}\mathrm{Father}+\beta_{11}{\mathrm{PGI Cog}}_{i}\times {\mathrm{PGI Cog}}_{i}\mathrm{Mother}+\beta_{12}{\mathrm{PGI Cog}}_{i}\times {\mathrm{PGI NonCog}}_{i}\mathrm{Father}+\beta_{13}{\mathrm{PGI Cog}}_{i}\times {\mathrm{PGI NonCog}}_{i}{\mathrm{Mother}+\beta }_{14}{\mathrm{PGI Cog}}_{i}\times Z_{i}+\beta_{15}{\mathrm{SES}}_{i}\times {\mathrm{PGI NonCog}}_{i}+\beta_{16}{\mathrm{SES}}_{i}\times {\mathrm{PGI Cog}}_{i}\mathrm{Father}+\beta_{17}{\mathrm{SES}}_{i}\times {\mathrm{PGI Cog}}_{i}\mathrm{Mother}+\beta_{18}{\mathrm{SES}}_{i}\times {\mathrm{PGI NonCog}}_{i}\mathrm{Father}+\beta_{19}{\mathrm{SES}}_{i}\times {\mathrm{PGI NonCog}}_{i}\mathrm{Mother}{+\beta }_{20}{\mathrm{SES}}_{i}\times Z_{i}+\varepsilon_{i}$$

(3d) $$Y_{i}=\alpha +{\beta_{1}{\mathrm{PGI Cog}}_{i}+\beta_{2}{\mathrm{PGI NonCog}}_{i}+\beta }_{3}{\mathrm{SES}}_{i}{+\beta }_{4}{\mathrm{PGI Cog}}_{i}{\mathrm{Father}+\beta }_{5}{\mathrm{PGI Cog}}_{i}\mathrm{Mother}+\beta_{6}{\mathrm{PGI NonCog}}_{i}\mathrm{Father}+\beta_{7}{\mathrm{PGI NonCog}}_{i}\mathrm{Mother}+Z_{i}+\beta_{8}{\mathrm{PGI NonCog}}_{i}\times {\mathrm{SES}}_{i}+{\beta_{9}{\mathrm{PGI NonCog}}_{i}\times {\mathrm{PGI Cog}}_{i}+\beta }_{10}{\mathrm{PGI NonCog}}_{i}\times {\mathrm{PGI Cog}}_{i}\mathrm{Father}+\beta_{11}{\mathrm{PGI NonCog}}_{i}\times {\mathrm{PGI Cog}}_{i}\mathrm{Mother}+\beta_{12}{\mathrm{PGI NonCog}}_{i}\times {\mathrm{PGI NonCog}}_{i}\mathrm{Father}+\beta_{13}{\mathrm{PGI NonCog}}_{i}\times {\mathrm{PGI NonCog}}_{i}{\mathrm{Mother}+\beta }_{14}{\mathrm{PGI NonCog}}_{i}\times Z_{i}+\beta_{15}{\mathrm{SES}}_{i}\times {\mathrm{PGI Cog}}_{i}+\beta_{16}{\mathrm{SES}}_{i}\times {\mathrm{PGI Cog}}_{i}\mathrm{Father}+\beta_{17}{\mathrm{SES}}_{i}\times {\mathrm{PGI Cog}}_{i}\mathrm{Mother}+\beta_{18}{\mathrm{SES}}_{i}\times {\mathrm{PGI NonCog}}_{i}\mathrm{Father}+\beta_{19}{\mathrm{SES}}_{i}\times {\mathrm{PGI NonCog}}_{i}\mathrm{Mother}{+\beta }_{20}{\mathrm{SES}}_{i}\times Z_{i}+\varepsilon_{i}$$

## 6 Results

### 6.1 Cognitive and noncognitive skills PGIs and educational outcomes

We first evaluate whether the PGIs for cognitive and noncognitive skills and family SES predict educational outcomes, as illustrated in Fig. 2, Fig. 3 by research designs (see Tables A4–A5, A7, A9–11 in the Appendix). The PGIs for cognitive and noncognitive skills and family SES positively predict educational outcomes regardless of the research design implemented, namely between-family, within-family, and trio designs.

Children with higher PGIs for cognitive skills have higher educational outcomes net of their family’s SES. The PGI for cognitive skills positively and significantly predict all outcomes except for adult educational attainment in the within-family design—with high uncertainty but similar effect size. Specifically, as shown in Figs. 2 and 3, a one-unit SD increase in the PGI for cognitive skills is associated with an increase of 0.13–0.23 points on a 1-to-5 scale (SD ∼ 0.9) for school grades, a 0.2–0.25 SD increase in CITO test scores, a 9–11 % higher chance of attending upper secondary tracks, or 7–10 % higher likelihood of attaining higher education in adulthood.

Similarly to PGI for cognitive, children with higher PGIs for noncognitive skills have higher educational outcomes net of their family’s SES. A one-unit SD increase in the PGI for noncognitive skills is associated with an increase of 0.06–0.10 points on a 1-to-5 scale for school grades, a 0.09–0.17 SD increase in CITO test scores, a 5–8 % higher chance of attending upper secondary tracks, or 6–10 % higher likelihood of attaining higher education in adulthood. One can note that the coefficients for the noncognitive skills PGI are, depending on the outcome, from 14 to 55 % smaller when compared to the cognitive skills PGI. However, they are still positive and statistically significant at 5 % across the different designs after adjusting for the family’s SES and all covariates, except for reading at age 10 in the within-family analysis—with high uncertainty but similar effect sizes.

Family SES is also positively associated with children’s educational outcomes. Controlling for children’s PGIs for cognitive and noncognitive skills—and parents’ PGIs in the trio design, individuals with highly educated parents get 0.2–0.3 points higher school grades, score 0.4 SD higher in the CITO exam, and have about 20 % higher likelihood to attend upper secondary tracks or university education than their least advantaged peers. One can notice that the PGIs and family SES estimates are remarkably similar across the three designs, going against the expectation that rGE upwardly biases between-family estimates considerably.

### 6.2 Heterogenous genetic associations on educational outcomes by family SES

We test whether the impact of PGIs for cognitive and noncognitive skills on educational outcomes varies according to the family SES. Fig. 4 shows the coefficients of the interactions between the PGIs for cognitive skills and the family’s SES. Focusing on school grades, we find a negative and statistically significant (p-value < 0.05) interaction only for mathematics at age 10 in the within-family and trio designs. Regarding CITO scores, the coefficients are substantial and have a negative sign in all three designs but are not statistically significant. We find a negative and statistically significant interaction (p-value < 0.05) for upper secondary tracks across all three designs, surviving Romano-Wolf multiple testing correction in the between and trio designs and Bonferroni correction in the between-family design (see Table A18 in the Appendix). Concerning educational attainment, there is a negative interaction statistically significant (p-value < 0.01), surviving Bonferroni correction, only in the between-family design, while in the within-family and trio designs, the estimates are non-significant and decrease by half but keep their negative sign. Tables A6, A8 and A12 in the Appendix show the interaction coefficients of these analyses for each model.

The pattern is less clear when looking at the interaction between PGI for noncognitive skills and the family SES, as shown in Fig. 5. If we focus on school grades, we observe no consistent and statistically significant interactions with any of these outcomes across research designs, except for a positive and significant interaction (p-value < 0.05) for reading at age 7 in the within-family design not surviving the Romano-Wolf correction. Regarding the remaining outcomes, the between-family design detects statistically significant negative interactions for upper secondary tracks (p-value < 0.05) and adult educational attainment (p-value < 0.01), while the trio design does the same for CITO test scores (p-value < 0.05) and tracking (p-value < 0.05), with a suggestively (p-value < 0.10) negative interaction for educational attainment of similar effect size as in the between-family model. Interestingly, non-statistically significant but negative interactions of similar size are found for tracking and educational attainment in the within-family design. Adjusting interaction p-values for multiple testing, Romano-Wolf correction confirms a negative interaction for CITO in the trio design, as well as tracking and educational attainment in the between-family design. The latter also survives Bonferroni correction (see Tables A19 in the Appendix). Tables A6, A8 and A12 in the Appendix show the interaction coefficients of these analyses for each model.

**Fig. 6:**
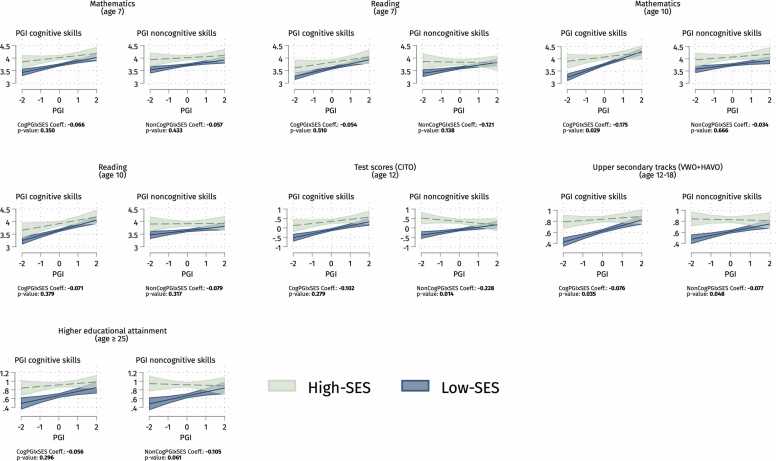
Predicted grades in mathematics and reading, CITO scores, and predicted probabilities of upper secondary tracks attendance and higher educational attainment by cognitive and noncognitive PGIs for children from low- and high-SES families (trio analysis). Note: 95 % confidence intervals. Standard errors clustered by families. We run OLS and LPM models with controls. Following Keller (2014), gene-covariates (PGIs) and environment-covariates (family’s SES) interactions are included. See sample sizes in Table 1. PGIs and CITO test scores are z-standardised.

For a clearer understanding of our findings, Fig. 6 plots the G × E interaction models by PGIs and outcomes for the trio design since this is the most reliable method (Breinholt & Conley, 2023). We focus on the tracking outcome since, as shown in Fig. 4, Fig. 5, its negative interaction between family SES and PGI for cognitive skills is the most consistent result by designs and multiple testing corrections. Looking at the probability of upper secondary attendance in Fig. 6, the slope of the PGI for cognitive skills is considerably flatter among high-SES children compared to low-SES peers, meaning that a low genetic propensity for cognitive skills is less consequential for advantaged children compared with disadvantaged pupils. While about 80 % of high-SES students with low PGI for cognitive skills (− 2 SD) attend the upper secondary tracks, only 40 % of low-SES with low PGI for cognitive skills do the same. Therefore, educational inequalities by family SES seem to be the largest among students at the bottom of the genetic distribution for cognitive skills, while this SES gap narrows progressively at the medium top.

Fig. 6 also shows a similar pattern for mathematics at age 10 and test scores (CITO). Looking at the interaction between family SES and the cognitive skills PGI on mathematics at age 10, we can see that the cognitive skills PGI is more predictive among low-SES than high-SES. Interestingly, the interaction between noncognitive skills PGI and family SES is negative and statistically significant also on test scores (CITO), with the PGI for noncognitive skills positively predicting the outcome for low-SES and the opposite for high-SES.

Overall, 93 % (39/42) of the estimated interactions between PGIs and SES are negative, contrasting with the Scarr-Rowe hypothesis (H1). Indeed, the Scarr-Rowe hypothesis lacks substantial empirical support, with only 3/42 positive interactions and only 1 out of 3 being statistically significant (p-value < 0.05) but not surviving multiple testing corrections. On the other hand, the overall negative direction in 39 out of 42 PGI × SES interactions, with 10 being statistically significant at 5 %, seems more in line with the compensatory advantage hypothesis (H2). When we account for multiple comparisons to prevent false positives due to chance, 7 detected negative interactions pass the Romano-Wolf correction threshold, but only 3 survive the more conservative Bonferroni correction (p-value < $\frac{0.05}{7\mathrm{outcomes by PGI}}$ = 0.007). We recognise the need for caution in interpreting these results as they are not fully robust to multiple testing, and the smaller samples of the within-family and trio designs and adult educational attainment could undermine the statistical power to identify false negatives reliably.

### 6.3 Robustness checks

We run several robustness checks to assess the credibility of our findings. First, using a linear model in studying interactions may lead to bias and false discovery in dichotomous or categorical outcomes (Domingue et al., 2022, Rohrer and Arslan, 2021, Mize, 2019). Thus, we (re)estimate our analysis using logistic models for dichotomous outcomes and nonparametric PGIs specified in terciles to successfully replicate the compensatory patterns found in the main analyses (see Figs. A4–A15 and Tables A23–A27 in the Appendix). These results confirm the negative interaction between family SES and PGI cognitive skills on the upper secondary track.

Second, we replicate the models using alternative definitions of our samples. On the one hand, we run the between-family and trio analyses without excluding one random MZ twin per family (see Tables A28–A33 in the Appendix). We find consistent results with the main samples and analyses. Again, the interactions between family SES and cognitive skills’ PGI replicate in the between-family and trio designs. On the other hand, regarding educational attainment, we run additional analyses with (1) the full ANTR sample without excluding individuals born before 1980 and (2) excluding those born after 1980 (see Tables A34–A39 in the Appendix). Splitting the educational attainment subsamples shows stable negative interaction coefficients in the between-family models but not in the within-family and trio designs, where its sign varies by birth cohort. Interestingly, focusing on the trio-design results and the subsample of cohorts born before 1980, the interaction coefficient becomes positive and statistically significant (p-value < 0.05) for the cognitive skills PGI in line with the Scarr-Rowe hypothesis. This latter pattern suggests that there might be heterogeneity in the G × E by birth cohorts and that this also could reflect changing selectivity in attaining a university degree in older cohorts (Ghirardi & Bernardi, 2023).

Finally, we replicate our analyses by building alternative definitions of parental and children’s educational attainment. We use the highest parental occupation drawing from the earliest available information, as with parental education (see Tables A40–42 in the Appendix). Results align with the main analysis concerning the G × E interactions for the upper secondary track, while the results for the G × E interaction on school grades for cognitive skills PGI in mathematics at age 10 and noncognitive skills PGI for CITO test scores are not statistically significant. Then, we repeat the analysis without dichotomising the children’s educational attainment outcome variable. We still distinguish between the sample used in the main analysis (born from 1980) and the other two split samples presented above (i.e., the overall sample and those born before 1980). In this robustness check, educational attainment consists of the following four categories: primary school only, lower vocational school and lower secondary school, intermediate vocational school and intermediate or higher secondary school, higher vocational school, and university. Findings align with the main results (see Tables A43–A45 in the Appendix).

## 7 Conclusion and discussion

This article examines the intergenerational transmission of educational inequality by testing whether genetic endowments for education matter differently by family SES. We investigate G × E interactions framed in behavioural genetics and social stratification theories to examine the competing Scarr-Rowe (H1) and compensatory advantage hypotheses (H2). While the former expects a positive interaction, arguing that high-SES families enhance full children’s genetic expression, the latter predicts a negative pattern where high-SES families compensate for their children’s low genetic endowments.

We provide three contributions to shed new light on previously mixed findings on these hypotheses. First, we look at G × E on educational phenotypes by untangling the genetic architecture of educational attainment—total years of education in adulthood—into cognitive and noncognitive skills, the main predictors of learning and educational performance. Second, due to data constraints, most studies implemented between- or within-family research designs that cannot fully account for gene-environment confounding. Few studies applied the trio design to enhance causal identification, which is ideal for combining parental genetic information with between-family models. Its application was limited to the US and Norway, countries with comprehensive educational systems. Hinging on Dutch panel family data, we triangulate findings from between-, within-family, and trio designs to bypass rGE in an early-tracked educational system. Third, while most previous research only looked at single outcomes with cross-sectional data, we analysed seven childhood-to-adulthood educational outcomes with different selectivity and implications for social demotion avoidance: grades and high-stakes test scores in primary education, school tracking in secondary education and adult attainment.

We report four main findings and discuss their implications for future research. First, the evidence presented in this study shows no empirical support for the *Scarr-Rowe hypothesis* (H1) since most G × E interactions estimated between cognitive and noncognitive skills PGIs and family SES were of negative sign (out of 42 G × E interactions, only 3 were positive with 2 non-significant and 1 significant not surviving multiple testing corrections). The weight of the evidence leans more into the competing *compensatory advantage hypothesis* (H2), with 39/42 negative G × E interaction coefficients that hold generally consistent across different research designs and PGIs. Still, we should be particularly cautious if we take our findings at face value. Out of 39 negative G × E interaction terms, only 7 are statistically significant under the conventional 5 % threshold and the Romano-Wolf multiple-testing adjustment, while barely 3 survived the most stringent Bonferroni correction (p-value < 0.007) to prevent false positives.

Second, our findings suggest that previously mixed findings on G × E interactions on educational attainment might relate to the type of outcomes investigated. We found evidence for G × E interactions among specific outcomes, only documenting a sound negative G × E interaction—robust across research designs and surviving multiple testing corrections—for the cognitive skills PGI and attendance to school tracks leading to college. In line with this finding, previous social stratification studies found that high-SES parents tend to compensate for their children’s low academic ability and performance (i.e., GPA, test scores, grade repetition) to keep progressing into academic pathways bound to college (Bernardi & Triventi, 2018).

Still, focusing on the trio as the most robust design accounting for parental genetics, findings suggest a negative G × E interaction for the noncognitive skills PGI when predicting high-stakes test scores (age 12) and upper-secondary tracking, and the cognitive skills PGI on math grades (age 10). These findings align with previous evidence that high-SES schools can compensate for a low PGI for educational attainment regarding mathematics persistence across secondary education in the US (Harden et al., 2020) or test scores in Norway (Cheesman et al., 2022, Cheesman et al., 2022). Examining single outcomes with snapshots might conceal that educational attainment results from successive academic achievements and transitions over the educational system. A life course approach based on longitudinal data allows for tracing educational careers and focusing on those educational outcomes that are especially critical for social demotion avoidance and future SES attainment (i.e., early track choice). This life-course approach might shed further light on G × E interactions and mechanisms in future studies.

Third, we expected inflation of our between-family estimates due to rGE, which would be visible by attenuation in the within-family and trio designs. However, the direction and magnitude of the estimated coefficients are broadly consistent across research designs. The main effects of PGIs for cognitive and noncognitive skills and family SES on educational outcomes suggest that parental education fully accounts for passive rGE mechanisms influencing children’s education (Selzam et al., 2019). Likewise, adjusting for family SES and its interactions with covariates might suffice to control for inflation of G × E interactions due to rGE. Exploring the replication of this result with alternative polygenic scores or various environmental factors could yield further insights.

Fourth, we found more robust negative G × E interactions for the genetic propensity for cognitive skills than noncognitive skills. This pattern might be related to the higher predictive power of the PGI for cognitive skills or due to the definition of noncognitive skills used in the GWAS (residual variance of adult educational attainment), which might make this PGI less reflective of noncognitive aspects of childhood education (Demange et al., 2021). This pattern calls for further research to study complementarity or substitution dynamics between cognitive and noncognitive genetic endowments and skill formation over the life course. In this direction, Malanchini et al. (2023) suggest that the contribution of the PGI for noncognitive skills to academic achievement is most prominent later in life.

This study has some limitations that should be considered for improvement in future research. First, interaction analyses are sensitive to the definition and distribution of outcome variables (Domingue et al., 2022). Using a linear model in studying G × E interactions with censored outcomes may lead to bias and false discovery when the outcome is dichotomous or categorical. Our sensitivity analyses estimating nonlinear specifications showed consistent results with the main linear analyses. Still, our study could be refined in future replication analyses to keep up with the continuous methodological advancements in sociogenomics in PGI prediction and interaction estimation, such as implementing variance PGIs (Johnson et al., 2022, Miao et al., 2022).

The second limitation is the small number of observations in some subsamples, especially for the within-family and trio designs and the adult educational attainment outcome. The larger the sample size, the higher the reliability in detecting G × E interactions (Domingue et al., 2020). We, indeed, explored this potential limitation in two ways. Firstly, we estimate the minimum incremental $R^{2}$ that would yield a statistically significant result using an F-test and assuming a statistical power of 0.8 in each subsample (see Tables A13–A15 and Figs. A1–A3 in the Appendix). Our analyses indicate that including an additional tested covariate, the interaction term, significantly changes the model’s explanatory power. Secondly, using Monte Carlo simulation, we conducted a post-hoc power analysis confirming that sufficient power was only achieved in the between-family analysis for specific outcomes, such as school tracking and educational attainment (see Tables A16–A17 in the Appendix). One should note that post-hoc power calculations (i.e., calculating statistical power using observed effect sizes) might not be fully informative (Gelman, 2019, Zhang et al., 2019). We argue that future research on G × E interactions should seriously address statistical power to better detect false negatives and minimise the risk of false positives using a larger sample size or meta-analysis (IntHout et al., 2016). Thus, we caution that our findings might be subject to replication when bigger samples are available in the Netherlands or other countries.

Third, we used different analytical samples and units of analysis according to the research design and the outcome examined. Thus, the differences in the results by outcome and over the between-, within-family, and trio designs might be due to successfully isolating different confounding sources, statistical power, as outlined above, or sample selection bias. However, as briefly discussed previously, this does not seem to be a concern since there are not considerable sociodemographic, genetic or educational differences between the various subsamples used in this study.

Fourth, the *Netherlands Twin Registry* study does not fully represent the Dutch population, raising concerns about positive selection and external validity as in most twin studies. Unfortunately, NTR data lacks sampling and longitudinal weights, which could help re-weight our analysis and increase our findings’ generalizability. Furthermore, as standard in sociogenomics studies relying on PGIs, our analysis is restricted to individuals with European ancestry available in current GWAS. Still, genetic variant associations might vary across populations with different ancestries (Martin et al., 2019). We hope future G × E studies will combine large-scale genotyped family data with longitudinal administrative information to improve power and population coverage and extend these analyses to be more inclusive of ethnic minorities.

While acknowledging these limitations to guide future research, this study indicates that the Scarr-Rowe hypothesis lacks empirical support. In contrast, its competing compensatory advantage hypothesis finds partial support, suggesting that advantaged families might offset the impact of low genetic propensity for cognitive and noncognitive skills and contribute to the intergenerational reproduction of educational inequality. At the same time, these findings, limited to parental education and occupation as proxies of the family SES environment, spark investigation of enriched learning environments, for instance, in schooling systems, and its policy potential to lift students with low genetic endowments for academic skills. Looking at mechanisms explaining our findings was beyond the scope of this study. Thus, we hope future studies will investigate the mechanisms underlying the interaction process between family SES and genetic propensity for education, such as parental educational investments and expectations, to shed light on the complex intertwining between DNA and social environments.

## CRediT authorship contribution statement

**Carlos J. Gil-Hernández:** Conceptualization, Data curation, Formal analysis, Investigation, Methodology, Project administration, Resources, Software, Supervision, Validation, Visualization, Writing – original draft, Writing – review & editing. **Gaia Ghirardi:** Conceptualization, Data curation, Formal analysis, Investigation, Methodology, Project administration, Resources, Software, Supervision, Validation, Visualization, Writing – original draft, Writing – review & editing. **Elsje van Bergen:** Conceptualization, Methodology, Supervision, Writing – review & editing, Resources. **Fabrizio Bernardi:** Conceptualization, Methodology, Project administration, Supervision, Writing – review & editing. **Perline Demange:** Conceptualization, Methodology, Resources, Supervision, Writing – original draft, Writing – review & editing.

## Declaration of Competing Interest

None.

## Footnotes

2 From now on, for the sake of simplicity, we generically use parental or family SES to refer to children’s social background even when in this article we measure it with the highest parental educational attainment.

3 This compensatory mechanism could work in absolute terms independently of relative within-family resource allocation patterns across siblings (i.e., parents investing relatively more in the sibling with higher PGI for education than in the sibling with lower PGI) (Engzell & Hällsten, 2022).

4 In early secondary schools (grade 7 and 8), many students are in bridge classes typically that include two out of three tracks. Most bridge classes take one or two years, so by grade 9, most students have been funnelled into a single school track. Two adjacent tracks are often grouped, for instance, VMBO-t/HAVO classes or HAVO/VWO classes.

5 Ethical approval for the NTR was provided by the Central Ethics Committee on Research Involving Human Subjects of the VU. University Medical Center, Amsterdam, and Institutional Review Board certified by the US Office of Human Research Protections (IRB number IRB-2991 under Federal-wide Assurance 3703; IRB/institute codes 94/105, 96/205, 99/068, 2003/182, 2010/ 359) and participants provided informed consent.

6 Regarding reading ability, the average of the NTR twins was 0.18 SD above the national average (van Bergen et al., 2018). NTR twins read equally well as their non-twin siblings. Thus, NTR families perform on average slightly higher, and twins are representative of non-twins. Besides, drawing from own analyses exploiting the Dutch PISA data from 2000 to 2018, overlapping with the NTR birth cohorts, by grade 9, 44 % of Dutch 15-year-olds’ pupils were enrolled in HAVO or VWO tracks, while 56 % were attending the VMBO tracks. In the YNTR data, 65 % of students attended the HAVO/VWO tracks, while 35 % the VMBO tracks. These figures imply that there is a slight positive selection in terms of ability in the NTR data.

7 In the Appendix, we display a robustness check analysis (Section 7.3) for the between-family and trio design analyses where we keep both MZ-twins and results hold.

8 To get an idea of the required sample size to study G × E interactions with enough statistical power, we reviewed the most recent studies examining the interaction between educational PGIs and parental SES (see Table A1 in the Appendix). Moreover, we conduct two types of power analysis (see Section 5 in the Appendix). First, we compute the minimum incremental $R^{2}$ for each subsample with a power of 80 % and a statistical significance of 5 %. This analysis suggests that, by including an additional tested covariate (i.e., the interaction) in each sample, we can confidently observe a significant change in the model’s explanatory power (see Tables A13–A15 and Figs. A1–A3 in the Appendix). Second, we conduct a post-hoc power analysis using Monte Carlo simulations (see Tables A16–A17 in the Appendix). These analyses suggest that power is often below standard levels of acceptability (power < 0.8). However, this is not the case for upper secondary track and educational attainment, at least in the between-family analysis where the sample sizes are higher. Thus, we suggest cautiously interpreting interaction effects when sample sizes and the beta coefficients are low.

9 In the pre-registration, we included teachers’ recommendation for the type of secondary school at age 12 as an additional outcome. However, the analytical sample sizes of this variable were very low, thus, we do not include it in the final analyses (see Section 2 in the Appendix).

10 Self-reported variables by parents might contain measurement. In case of systematic misreporting by family SES, we might observe a biased G × E interaction but, to our knowledge, there is no evidence on data quality issues in the NTR data.

11 In our sample, 90 % of students, on average, remain on the same track from age 14 to 18.

12 Variance PGIs is a promising strategy to more reliably estimate G × E interactions (Johnson et al., 2022, Miao et al., 2022). Unfortunately, we use mean PGIs since, at the moment, there are few available vQTL/vGWAS summary statistics and no well-powered educational attainment or cognitive skills studies to our knowledge. Additionally, it is unclear how Genomic SEM might be able to leverage vQTL summary statistics.

13 The summary statistics of the GWAS for cognitive and non-cognitive skills can be found at the following link: https://dataverse.nl/dataset.xhtml?persistentId=doi:10.34894/MMXYPL.
