# Supplementary material for: Interaction of family SES with children’s genetic propensity for cognitive and noncognitive skills: No evidence of the Scarr-Rowe hypothesis for educational outcomes
Source: Res Soc Stratif Mobil. 2024 Aug;92:100960. doi: 10.1016/j.rssm.2024.100960 (PMC11364161; doi:10.1016/j.rssm.2024.100960)
Supplement: Supplementary file 1 — Supplementary material [file mmc1.pdf]

# Appendix

## Table of contents

1. Literature review ... 3
2. Differences between pre-registered study and final version ... 7
3. Samples characteristics and descriptive statistics ... 7
4. Main analyses tables ... 10
4.1. Between-family analysis ... 10
4.2. Within-family analysis ... 11
4.3. Trio design ... 12
5. Power analysis ... 16
5.1. Power analysis for an $R^2$ test in a multiple linear regression ... 16
5.2. Post-hoc power analysis using Montecarlo simulation ... 19
5.2.1. Assumptions ... 19
5.2.2. Results ... 20
5.2.3. Replicability ... 20
6. Multiple testing ... 20
7. Robustness checks ... 22
7.1. Alternative control variables ... 22
7.1.1 Between-family design ... 22
7.1.2 Within-family design ... 22
7.1.3 Trio design ... 23
7.2. Alternative model specifications ... 24
7.2.1. Logistic regression models ... 24
7.2.1.1 Between-family design ... 24
7.2.1.2 Within-family design ... 25
7.2.1.3 Trio design ... 26
7.2.2. Non-linear PGIs models for continuous outcomes ... 27
7.2.2.1 Between-family design ... 27
7.2.2.2 Trio design ... 29
7.2.3. Logistic regression models and non-linearities in the PGI ... 32
7.2.3.1. Between-family design ... 32
7.2.3.2. Trio design ... 34
7.3. Alternative samples ... 36
7.3.1. Between-family with all MZ twins ... 36
7.3.2. Trio analysis with all MZ twins ... 37
7.3.3. Educational attainment: all participants and born before/after 1980 ... 38
7.3.3.1 Between-family design ... 38
7.3.3.2 Within-family design ... 39
7.3.3.3. Trio design ... 40

7.4. Alternative measure of SES: Parents' occupation ........ 41
7.4.1. Between-family design ........ 41
7.4.2. Within-family design ........ 41
7.4.3. Trio design ........ 42

7.5. Educational attainment as continuous outcome ........ 42
7.5.1 Between-family design ........ 42
7.5.2 Within-family design ........ 43
7.5.3 Trio design ........ 43

## 1. Literature review

Table A1: Literature review of studies that investigate the interaction between genetic propensity to educational attainment, cognitive and noncognitive skills and socio-economic background using molecular data.

| Study | Country | Data | Main design | SES measure | PGI version | Stronger in high-SES | Stronger in low-SES | No interaction |
|---|---|---|---|---|---|---|---|---|
| Conley et al., 2015 | US | Framingham Heart Study (FHS), Health and Retirement Study (HRS) | Between-family and within-family | Family's SES: mothers' education | PGI EA2 | | | X<br>For years of education |
| Trejo et al., 2018 | US | The Wisconsin Longitudinal Study (WLS) National Longitudinal Study of Adolescent to Adult Health (Add Health) | Between-family | School SES: % of students in school with mother graduated high school and Gini coefficient | PGI EA3 | X<br>For college completion | | |
| de Zeeuw et al., 2019 | NL | Netherlands Twin Register (NTR) | Between-family and within-family | Family's SES: parental job status, occupational and education level | PGI EA3 | | | X<br>For educational achievement (scholastic knowledge, including language and mathematical skills) |
| Harden et al., 2020 | US | National Longitudinal Study of Adolescent to Adult Health (Add Health) | Between-family | School SES: % of students in school with mother graduated high school | PGI EA3 | | X<br>For persistency in mathematics across the 4 | X<br>For mathematics tracking at grade 9th |

| | | | | | | | | |
|---|---|---|---|---|---|---|---|---|
| | | | | | | | years of high-school | |
| Papageorge and Thom, 2020 | US | The Health and Retirement Study (HRS) | Between-family | Family's SES: fathers' income, family well off, father's unemployment, moved asked for help | PGI EA3 | X<br>For college completion | X<br>For high-school completion | X<br>For labour market outcomes |
| Lin, 2020 | US | Health and Retirement Study (HRS) | Between-family | Family's SES: highest years of education attained by father or mother | PGI EA3 | | X<br>For educational attainment (in the following categories: No degree, GED/High school diploma, 2-year college degree/Some college, 4-year college degree, MA, PhD) | |
| von Stumm et al. 2020 | UK | Twins Early Development Study (TEDS) | Between-family | Family SES: mothers' and fathers' | PGI EA3 | | X<br>Grades: for English and mathematics | X<br>Change in grades: for English and mathematics |
| Uchikoshi and Conley, 2021 | US | National Longitudinal Study of Adolescent to Adult Health (Add Health) | Between-family | Family's SES: parental education, occupation, household income, receipt of public assistance | PGI EA3 | X<br>For tracking in mathematics in the 10th grade | | |
| Isungset et al., 2021 | NO | Norwegian Mother, Father and Child Cohort Study (MoBa) | Trio-design | Parents' education when parents are 30 years old | PGI EA3 | | | X<br>For children's academic test scores |

| | | | | | | | |
|---|---|---|---|---|---|---|---|
| | | | | | | | in Reading, English and Mathematics, taken in the 5th, 8th and 9th grades |
| Judd et al., 2021 | US | Adolescent Brain Cognitive Development (ABCD) study | Between-family and within-family | Family's SES: total household income, highest parental education, and neighborhood quality | PGI Cognitive skills (Lee et al., 2018) | | X<br><br>For cognitive skills (crystallised intelligence, fluid intelligence, and working memory) |
| Arold et al., 2022 | US | National Longitudinal Study of Adolescent to Adult Health (Add Health) | Between-family | School investment (teacher quality and teacher quantity) | PGI EA3 | X<br><br>Education attainment (years of education, high school, 2-year college degree, 4-year college degree, or completed a post-graduate degree) for the interaction with teacher quality | |
| Cheesman et al., 2022 | NO | Norwegian Mother, Father and Child Cohort Study (MoBa) | Between-family, within-family and trio desing | Schools and residential areas | PGI EA3 | X<br><br>Standardised national test results for maths and reading at grades 5, 8, and 9, and English | |

| | | | | | | | | |
|---|---|---|---|---|---|---|---|---|
| | | | | | | | at grades 5 and 8 for the interaction with school | |
| Ronda et al. 2022 | DK | Integrative Psychiatric Research (iPSYCH) study | Between-family and within-family | Family's SES: Parental human capital, family resources, family stability, and parental mental health | PGI EA3 | X<br>Years of education (only in the between-family), post-secondary education, Danish (only in the between-family), Mathematics | | |
| Malanchini et al., 2022 | UK | Twins Early Development Study (TEDS) | Twin analysis, Between-family and within-family | Family's SES: Index taking parents educational qualifications, employment, and mothers' age at first birth | PGI Cognitive and PGI Noncognitive skills (Demange et al., 2020) | | | X<br>Cognitive abilities (age 7, 9, 12, 16), Academic achievement (age 7, 9, 12, 16), Education specific noncognitive abilities (age 9, 12, 16), Self-regulation |
| Breinholt et al., 2023 | US | Future of Families and Child Wellbeing Study (FFCWS) | Between-family analysis | Family SES: Maternal education (0 = high school or less, 1 = more education than high school) and household income | PGI EA3 | | | X Cognitive skills (age 9) |

Note: PGI EA3: Polygenic index for educational attainment release 3 (Lee et al., 2018). PGI EA2: Polygenic index for educational attainment release 2 (Rietveld et al., 2013). Previous studies are ordered in chronological order.

## 2. Differences between the pre-registered study and the final version

Here we list and motivate a few differences between the pre-registration and the final version:

1. Regarding adult educational attainment, we restrict the analysis to those born from 1980 instead of taking the overall available sample while conducting robustness checks on the subsample born before 1980 (see below). This decision was motivated by having comparable samples of the historical period and educational system between the young and adult *Netherlands Twin Study* cohorts since the latter has a year of birth distribution ranging from 1909 to 2001.
2. We do not look at teachers' recommendations for the type of secondary school attended (at age 12) as an additional outcome due to its many missing observations. We thus decided to exclude it since we already included the actual academic track attended by the child.
3. We also added the genetic testing platform as a control variable in all models, as it can be a potential confounder.
4. In the pre-registration, we state the intention to perform a power analysis before data analyses to discard those underpowered outcomes/analytical samples and avoid the likelihood of observing false positives or not detecting true positives. Instead, we performed a post-hoc power analysis.
5. We estimated models accounting for *the prediction hypothesis* in the pre-registration (i.e., the impact of the PGI for cognitive and noncognitive skills on educational outcomes). However, for the sake of brevity in the final version of the paper, we decided to focus mainly on the Scarr-Rowe and compensatory advantaged hypotheses.
6. Regarding the theoretical expectations, although we discussed and speculated about the implications of the outcomes' timing for the main findings and how future research might address it, we did not specify or test any hypotheses about the timing of the outcomes. More specifically, in the pre-registration, we expect that:
    - The association between cognitive and noncognitive PGI would increase with age;
    - The GxE would be stronger for school grades than CITO scores;
    - The GxE would be stronger for tracking than CITO scores;
    - The GxE would be stronger for later outcomes than earlier.

## 3. Samples characteristics and descriptive statistics

The following are the main criteria used to select our analytical samples. We selected all children or adult participants in the NTR with the available:

1. Genotypic data;
2. Information on at least one of the following educational outcomes in all birth cohorts: school grades in numeracy and literacy, CITO at age 12, type of secondary school attended from age 12, and educational attainment;
3. Information on parental SES (i.e., parent's education).

Tables A2 and A3 below describe the overall and analytical samples, showing the share of missing values and representativeness of our subsamples compared to the overall NTR sample and population.

Table A2: Comparison between the population and the NTR (YNTR and ANTR samples) by full and analytical samples

| | Population | YNTR Samples | | | | ANTR Samples | | |
|---|---|---|---|---|---|---|---|---|
| Sample | | Full | Analytical[c] | | | Full | Analytical[f] | Analytical[g] |
| Age | | Age 1 | Age 7 | Age 10 | Age 12 | Age≥18 | Age≥25 | Age≥25 Birth≥1980 |
| Twins' Birth Year Mean (SD) | | 2000 (8) | 1995 (7) | 1996 (7) | 1994 (6) | 1990 (17) | 1972 (13) | 1987 (3) |
| Twins' Birth Year Range | 1986-2019 | 1986-2019 | 1986-2010 | 1986-2008 | 1986-2006 | 1909-2001 | 1909-1992 | 1980-1991 |
| Mother's Birth Year Mean (SD) | | 1969 (8) | 1964 (7) | 1964 (7) | 1963 (6) | 1963 (12) | 1949 (9) | 1957 (5) |
| Mother's Age at 1st Birth Mean | 28.9 | 32.1 | 33.1 | 33.2 | 33.3 | 32.6 | 32.6 | 33.7 |
| Number of Children Mean | 1.7 | 2.4 | 2.7 | 2.7 | 2.8 | 2.7 | 2.9 | 2.9 |
| Mother's Higher Education[h] Mean | 25.7%[a] | 28.6% | 35.0%[d] | 36.5% | 34.6% | 27.5% | 19.6% | 36.8% |
| Father's Higher Education[h] Mean | 29.7%[a] | 36.4% | 41.1%[d] | 42.2% | 41.1% | 36.1% | 31.4% | 47.8% |
| CITO Test Scores Mean (SD) | 536[b] | | | | 538.9 (8)[e] | | | |
| Total Number of Twins[i] | 98,877 | 68,771 | | | | | | |
| Total Sample Size (Twins + Siblings) | | 81,805 | 29,274 | 23,576 | 21,331 | 61,350 | 14,731 | 14,731 |
| Baseline Analytical Sample Size[j] | | | 3,874 | 3,920 | 3,318 | | | 1,187 |

Notes: Standard deviation between parentheses; [a] All women or men in 2003, aged 25-55 born 1948-1978 (Statistics Netherlands, 2024); [b] Population data from the academic year 2005/2006 (Statistics Netherlands, 2024); [c] Summary statistics from analytical samples for between-family models after listwise deletion of independent variables (Baseline Analytical Sample Size) or corresponding outcome (e.g., CITO); [d] Mean survey year = 2003; [e] Mean survey year = 2006; [f] ANTR subsample with adult educational attainment available (age ≥ 25); [g] ANTR subsample with adult educational attainment available (age ≥ 25) and children born from 1980; [h] Higher education = 31 Hbo-, wo-bachelor + 32 Hbo-, wo-master, doctor; [i] Born from 1986 to 2019 and excluding multiple twin set; population data from Statistics Netherlands (2024); [j] Baseline analytical sample size for the between-family design after listwise deletion of independent variables and before outcomes' listwise deletion (except CITO at age 12). Final analytical samples by research design and outcome in Table 1.

# Table A3: Missing data description for the YNTR and ANTR datasets

| | Missing | N | % | Missing | N | % | Missing | N | % | Missing | N | % | Missing | N | % |
|---|---|---|---|---|---|---|---|---|---|---|---|---|---|---|---|
| NTR Dataset | | | | | | | | YNTR | | | | | | ANTR | |
| Age | | 1 | | | 7 | | | 10 | | | 12 | | | ≥ 18 | |
| **Independent Variables (IVs)** | | | | | | | | | | | | | | | |
| Child Year of Birth | 22 | 81805 | 0.03 | 2 | 29274 | 0.01 | 0 | 23576 | 0 | 0 | 21331 | 0 | 62 | 61350 | 0.1 |
| Mother's Year of Birth* | 986 | 81805 | 1.21 | 646 | 29274 | 2.21 | 468 | 23576 | 1.99 | 547 | 21331 | 2.56 | 8210 | 61350 | 13.38 |
| Mother's Age at Birth* | 377 | 81805 | 0.46 | 60 | 29274 | 0.2 | 22 | 23576 | 0.09 | 30 | 21331 | 0.14 | 1502 | 61350 | 2.45 |
| Number of Children* | 0 | 81805 | 0 | 0 | 29274 | 0 | 0 | 23576 | 0 | 0 | 21331 | 0 | 0 | 61350 | 0 |
| Sex | 38 | 81805 | 0.05 | 2 | 29274 | 0.01 | 0 | 23576 | 0 | 0 | 21331 | 0 | 72 | 61350 | 0.12 |
| Zygosity* | 950 | 81805 | 1.16 | 30 | 29274 | 0.1 | 6 | 23576 | 0.03 | 22 | 21331 | 0.1 | 1528 | 61350 | 2.49 |
| Highest Parental Edu. | 29749 | 81805 | 36.37 | 3536 | 29274 | 12.08 | 847 | 23576 | 3.59 | 1755 | 21331 | 8.23 | 13044 | 61350 | 21.26 |
| Mother's Education* | 32886 | 81805 | 40.2 | 5436 | 29274 | 18.57 | 2375 | 23576 | 10.07 | 2974 | 21331 | 13.94 | 16336 | 61350 | 26.63 |
| Father's Education* | 41630 | 81805 | 50.89 | 8396 | 29274 | 28.68 | 4698 | 23576 | 19.93 | 5126 | 21331 | 24.03 | 19775 | 61350 | 32.23 |
| Family SES (Age 3) | 23336 | 81805 | 28.53 | 593 | 29274 | 2.03 | 36 | 23576 | 0.15 | 1328 | 21331 | 6.23 | 25975 | 61350 | 42.34 |
| Children's PGIs | 71595 | 81805 | 87.52 | 22770 | 29274 | 77.78 | 17299 | 23576 | 73.38 | 15914 | 21331 | 74.61 | 44627 | 61350 | 72.74 |
| Children's PGIs (EA) | 72960 | 81805 | 89.19 | 23620 | 29274 | 80.69 | 17985 | 23576 | 76.29 | 16597 | 21331 | 77.81 | 46885 | 61350 | 76.42 |
| Mother's PGIs | 73785 | 81805 | 90.2 | 24968 | 29274 | 85.29 | 19272 | 23576 | 81.74 | 17832 | 21331 | 83.6 | 49694 | 61350 | 81 |
| Father's PGIs | 75526 | 81805 | 92.32 | 25750 | 29274 | 87.96 | 20022 | 23576 | 84.93 | 18511 | 21331 | 86.78 | 52172 | 61350 | 85.04 |
| **Outcome Variables** | | | | | | | | | | | | | | | |
| Math - Age 7 | 55416 | 81805 | 67.74 | 1755 | 29274 | 6 | 7463 | 23576 | 31.66 | 6428 | 21331 | 30.13 | 43394 | 61350 | 70.73 |
| Reading - Age 7 | 55163 | 81805 | 67.43 | 1487 | 29274 | 5.08 | 7268 | 23576 | 30.83 | 6259 | 21331 | 29.34 | 43257 | 61350 | 70.51 |
| Math - Age 10 | 60115 | 81805 | 73.49 | 12971 | 29274 | 44.31 | 849 | 23576 | 3.6 | 6650 | 21331 | 31.18 | 45865 | 61350 | 74.76 |
| Reading - Age 10 | 59960 | 81805 | 73.3 | 12860 | 29274 | 43.93 | 678 | 23576 | 2.88 | 6551 | 21331 | 30.71 | 45785 | 61350 | 74.63 |
| CITO - Age 12 | 65525 | 81805 | 80.1 | 17746 | 29274 | 60.62 | 13034 | 23576 | 55.29 | 8449 | 21331 | 39.61 | 48056 | 61350 | 78.33 |
| Track - Age 12-18 | 62457 | 81805 | 76.35 | 16715 | 29274 | 57.1 | 11981 | 23576 | 50.82 | 7248 | 21331 | 33.98 | 45148 | 61350 | 73.59 |
| Adult Education - Age ≥ 25 | 78304 | 81805 | 95.72 | 27605 | 29274 | 94.3 | 21895 | 23576 | 92.87 | 19731 | 21331 | 92.5 | 46619 | 61350 | 75.99 |
| **Analytical Sample: IVs Listwise Deletion and Sample Selection** | | | | | | | | | | | | | | | |
| Listwise deletion IVs | | | | 23840 | 29274 | 81.44 | 18123 | 23576 | 76.87 | 16710 | 21331 | 78.34 | 48007 | 61350 | 78.25 |
| Excluding 1 Random MZ-twin + Multiple Twin Sets | | | | 1560 | 5434 | 28.71 | 1533 | 5453 | 28.11 | 794 | 4621 | 17.18 | 3501 | 13343 | 26.24 |
| **Analytical Sample by Research Design before Outcomes' Listwise Deletion (see Table 1 for analytical sample after outcomes' listwise deletion)** | | | | | | | | | | | | | | | |
| Between | | | | | 3874 | | | 3920 | | | 3318 | | | 9842 | |
| Within (Balanced Family FE) | | | | | 3299 | | | 3341 | | | 2790 | | | 7070 | |
| Trio (Missing Parental PGS) | | | | | 1984 | | | 2094 | | | 1618 | | | 4646 | |

N = All twins and siblings; *Variables not used in the analyses; EA = European Ancestry; Shaded squares in grey correspond to the survey wave when the outcome's is mainly reported. The final analytical samples by research design and outcome are in Table 1.

## 4. Main analyses tables

### 4.1. Between-family analysis

Table A4: OLS (mathematics, reading achievement and CITO) and LPM (academic tracking and educational attainment) regressions to test the association between children's cognitive and noncognitive PGI and educational outcomes without including family SES.

| | Mathematics (age 7) | Reading (age 7) | Mathematics (age 10) | Reading (age 10) | Test scores CITO (age 12) | Upper secondary track (age 12-18) | Educational Attainment (age ≥ 25) |
|---|---|---|---|---|---|---|---|
| Cognitive PGS | 0.163*** | 0.158*** | 0.214*** | 0.179*** | 0.282*** | 0.119*** | 0.110*** |
| | (0.015) | (0.015) | (0.016) | (0.016) | (0.020) | (0.009) | (0.013) |
| Noncognitive PGS | 0.0823*** | 0.0878*** | 0.111*** | 0.0944*** | 0.191*** | 0.0897*** | 0.0767*** |
| | (0.015) | (0.015) | (0.016) | (0.016) | (0.020) | (0.009) | (0.013) |
| Observations | 3728 | 3756 | 3829 | 3875 | 2690 | 3318 | 1224 |
| Adjusted R2 | 0.0474 | 0.0413 | 0.0564 | 0.0377 | 0.108 | 0.0962 | 0.0611 |

Note: Robust standard errors in parentheses. Two-tailed t-test: $^{+}$ $p < 0.10$, $^{*}$ $p < 0.05$, $^{**}$ $p < 0.01$, $^{***}$ $p < 0.000$ Controls included but not reported above: first 10 PCs and Platform.

Table A5: OLS (mathematics, reading and CITO) and LPM (academic tracking and educational attainment) regressions to test the association between children's cognitive and noncognitive PGI and educational outcomes including family SES.

| | Mathematics (age 7) | Reading (age 7) | Mathematics (age 10) | Reading (age 10) | Test scores CITO (age 12) | Upper secondary track (age 12-18) | Educational Attainment (age ≥ 25) |
|---|---|---|---|---|---|---|---|
| Cognitive PGS | 0.150*** | 0.146*** | 0.195*** | 0.160*** | 0.253*** | 0.105*** | 0.0953*** |
| | (0.015) | (0.016) | (0.017) | (0.016) | (0.020) | (0.009) | (0.013) |
| Noncognitive PGS | 0.0718*** | 0.0778*** | 0.0964*** | 0.0798*** | 0.168*** | 0.0791*** | 0.0655*** |
| | (0.015) | (0.015) | (0.016) | (0.016) | (0.020) | (0.009) | (0.013) |
| SES (ref: Low-SES) | 0.175*** | 0.166*** | 0.244*** | 0.239*** | 0.411*** | 0.188*** | 0.192*** |
| | (0.037) | (0.040) | (0.040) | (0.041) | (0.046) | (0.020) | (0.026) |
| Observations | 3728 | 3756 | 3829 | 3875 | 2690 | 3318 | 1224 |
| Adjusted R2 | 0.0536 | 0.0463 | 0.0664 | 0.0472 | 0.134 | 0.118 | 0.0865 |

Note: Robust standard errors in parentheses. Two-tailed t-test: $^{+}$ $p < 0.10$, $^{*}$ $p < 0.05$, $^{**}$ $p < 0.01$, $^{***}$ $p < 0.000$. Controls included but not reported above: first 10 PCs and Platform.

Table A6: OLS and LPM (academic tracking and educational attainment) regressions to test the interaction between children's cognitive and noncognitive PGI and family SES on educational outcomes.

| | Mathematics (age 7) | Reading (age 7) | Mathematics (age 10) | Reading (age 10) | Test scores CITO (age 12) | Upper secondary track (age 12-18) | Educational Attainment (age ≥ 25) |
|---|---|---|---|---|---|---|---|
| SES x PGS Cognitive | -0.0433 | -0.0145 | -0.0729+ | -0.00573 | -0.0911+ | -0.0645*** | -0.109*** |
| | (0.037) | (0.042) | (0.042) | (0.039) | (0.048) | (0.019) | (0.027) |
| Observations | 3728 | 3756 | 3829 | 3875 | 2690 | 3318 | 1224 |
| Adjusted R2 | 0.0554 | 0.0464 | 0.0659 | 0.0451 | 0.133 | 0.120 | 0.0914 |
| SES x PGS Noncognitive | -0.0107 | -0.00875 | -0.0185 | -0.0170 | -0.0588 | -0.0473* | -0.106*** |
| | (0.037) | (0.041) | (0.039) | (0.039) | (0.044) | (0.019) | (0.028) |
| Observations | 3728 | 3756 | 3829 | 3875 | 2690 | 3318 | 1224 |
| Adjusted R2 | 0.0554 | 0.0450 | 0.0656 | 0.0462 | 0.133 | 0.121 | 0.0960 |

Note: Robust standard errors in parentheses. Two-tailed t-test: $^{+}$ $p$ < 0.10, $^{*}$ $p$ < 0.05, $^{**}$ $p$ < 0.01, $^{***}$ $p$ < 0.000. Controls included but not reported above: first 10 PCs and Platform. And all controls in table A2. We also include covariates-environment (family's' SES) and covariates-gene (PGI) interaction (Keller, 2014).

## 4.2. Within-family analysis

Table A7: Family-fixed effect regressions to test the association between children's cognitive and noncognitive PGI and educational outcomes.

| | Mathematics (age 7) | Reading (age 7) | Mathematics (age 10) | Reading (age 10) | Test scores CITO (age 12) | Upper secondary track (age 12-18) | Educational Attainment (age ≥ 25) |
|---|---|---|---|---|---|---|---|
| Cognitive PGS | 0.128*** | 0.137*** | 0.202*** | 0.164*** | 0.216*** | 0.100*** | 0.0700 |
| | (0.032) | (0.032) | (0.037) | (0.033) | (0.042) | (0.017) | (0.043) |
| Noncognitive PGS | 0.0737* | 0.0806* | 0.0960** | 0.0564 | 0.172*** | 0.0663*** | 0.0966* |
| | (0.031) | (0.032) | (0.035) | (0.035) | (0.042) | (0.018) | (0.046) |
| Observations | 2124 | 2130 | 2212 | 2236 | 1500 | 2004 | 426 |
| Adjusted R2 | 0.0184 | 0.0154 | 0.0324 | 0.0177 | 0.0524 | 0.0323 | 0.0550 |

Note: Robust standard errors in parentheses. Two-tailed t-test: $^{+}$ $p$ < 0.10, $^{*}$ $p$ < 0.05, $^{**}$ $p$ < 0.01, $^{***}$ $p$ < 0.000. Controls included but not reported above: first 10 PCs and Platform.

Table A8: Family-fixed effect regressions to test the interaction between children's cognitive and noncognitive PGI and family SES on educational outcomes.

| | Mathematics (age 7) | Reading (age 7) | Mathematics (age 10) | Reading (age 10) | Test scores CITO (age 12) | Upper secondary track (age 12-18) | Educational Attainment (age ≥ 25) |
|---|---|---|---|---|---|---|---|
| SES x PGS Cognitive | -0.0288 | -0.107 | -0.174* | -0.0441 | -0.139 | -0.0795* | -0.0414 |
| | (0.089) | (0.089) | (0.087) | (0.092) | (0.087) | (0.038) | (0.094) |
| Observations | 2124 | 2130 | 2212 | 2236 | 1500 | 2004 | 426 |
| Adjusted R2 | 0.0457 | 0.0327 | 0.0605 | 0.0310 | 0.0653 | 0.0475 | 0.120 |
| SES x PGS Noncognitive | -0.0653 | 0.169* | -0.113 | 0.0212 | 0.0162 | -0.0412 | -0.0881 |
| | (0.089) | (0.084) | (0.093) | (0.100) | (0.097) | (0.043) | (0.088) |
| Observations | 2124 | 2130 | 2212 | 2236 | 1500 | 2004 | 426 |
| Adjusted R2 | 0.0378 | 0.0398 | 0.0509 | 0.0301 | 0.0571 | 0.0523 | 0.155 |

Note: Robust standard errors in parentheses. Two-tailed t-test: $^{+}$ $p$ < 0.10, $^{*}$ $p$ < 0.05, $^{**}$ $p$ < 0.01, $^{***}$ $p$ < 0.000. Controls included but not reported above: first 10 PCs and Platform. We also include covariates-environment (family's' SES) and covariates-gene (PGI) interaction (Keller, 2014).

## 4.3. Trio-design

Table A9: OLS (mathematics, reading and CITO) and LPM (academic tracking and educational attainment) regressions to test the association between children's cognitive and noncognitive PGI and educational outcomes using the sample of the trio-design, without controlling for family SES and parents cognitive and noncognitive PGI.

| | Mathematics (age 7) | Reading (age 7) | Mathematics (age 10) | Reading (age 10) | Test scores CITO (age 12) | Upper secondary track (age 12-18) | Educational Attainment (age ≥ 25) |
|---|---|---|---|---|---|---|---|
| Cognitive PGS | 0.173*** | 0.167*** | 0.199*** | 0.180*** | 0.253*** | 0.119*** | 0.107*** |
| | (0.022) | (0.023) | (0.023) | (0.023) | (0.030) | (0.012) | (0.020) |
| Noncognitive PGS | 0.0888*** | 0.0998*** | 0.118*** | 0.0980*** | 0.148*** | 0.0801*** | 0.0707*** |
| | (0.021) | (0.022) | (0.023) | (0.023) | (0.029) | (0.013) | (0.019) |
| Observations | 1861 | 1869 | 2022 | 2051 | 1254 | 1500 | 576 |
| Adjusted R2 | 0.0475 | 0.0431 | 0.0447 | 0.0370 | 0.0836 | 0.103 | 0.0433 |

Note: Robust standard errors in parentheses. Two-tailed t-test: $^{+}$ $p$ < 0.10, $^{*}$ $p$ < 0.05, $^{**}$ $p$ < 0.01, $^{***}$ $p$ < 0.000. Controls included but not reported above: first 10 PCs and Platform.

Table A10: OLS (mathematics, reading and CITO) and LPM (academic tracking and educational attainment) regressions to test the association between children's cognitive and noncognitive PGI and educational outcomes using the sample of the trio-design, controlling for family SES and not for parents cognitive and noncognitive PGI.

| | Mathematics (age 7) | Reading (age 7) | Mathematics (age 10) | Reading (age 10) | Test scores CITO (age 12) | Upper secondary track (age 12-18) | Educational Attainment (age ≥ 25) |
|---|---|---|---|---|---|---|---|
| Cognitive PGS | 0.153*** | 0.151*** | 0.174*** | 0.157*** | 0.214*** | 0.101*** | 0.0838*** |
| | (0.022) | (0.024) | (0.024) | (0.023) | (0.030) | (0.012) | (0.020) |
| Noncognitive PGS | 0.0738*** | 0.0870*** | 0.0996*** | 0.0814*** | 0.122*** | 0.0667*** | 0.0556** |
| | (0.022) | (0.022) | (0.023) | (0.023) | (0.029) | (0.013) | (0.019) |
| SES (ref: Low-SES) | 0.229*** | 0.195*** | 0.266*** | 0.244*** | 0.432*** | 0.211*** | 0.230*** |
| | (0.054) | (0.058) | (0.056) | (0.057) | (0.066) | (0.027) | (0.037) |
| Observations | 1861 | 1869 | 2022 | 2051 | 1254 | 1500 | 576 |
| Adjusted R2 | 0.0579 | 0.0497 | 0.0566 | 0.0470 | 0.113 | 0.132 | 0.0847 |

Note: Robust standard errors in parentheses. Two-tailed t-test: $^{+}$ $p < 0.10$, $^{*}$ $p < 0.05$, $^{**}$ $p < 0.01$, $^{***}$ $p < 0.000$. Controls included but not reported above: first 10 PCs and Platform.

Table A11: OLS (mathematics, reading and CITO) and LPM (academic tracking and educational attainment) regressions to test the association between children's cognitive and noncognitive PGI and educational outcomes using the sample of the trio-design, controlling for family SES and for parents cognitive and noncognitive PGI.

| | Mathematics (age 7) | Reading (age 7) | Mathematics (age 10) | Reading (age 10) | Test scores CITO (age 12) | Upper secondary track (age 12-18) | Educational Attainment (age ≥ 25) |
|---|---|---|---|---|---|---|---|
| Cognitive PGS | 0.138*** | 0.155*** | 0.228*** | 0.188*** | 0.201*** | 0.0866*** | 0.0755** |
| | (0.030) | (0.032) | (0.032) | (0.033) | (0.042) | (0.017) | (0.027) |
| Noncognitive PGS | 0.0801** | 0.0805** | 0.0814* | 0.0691* | 0.0924* | 0.0503** | 0.0605* |
| | (0.029) | (0.031) | (0.033) | (0.033) | (0.041) | (0.017) | (0.029) |
| SES (ref: Low-SES) | 0.226*** | 0.196*** | 0.275*** | 0.250*** | 0.426*** | 0.202*** | 0.227*** |
| | (0.056) | (0.059) | (0.057) | (0.058) | (0.067) | (0.028) | (0.038) |
| Cognitive PGI Mother | 0.0106 | -0.00715 | -0.0673* | -0.0420 | 0.0325 | 0.00192 | 0.00596 |
| | (0.027) | (0.029) | (0.029) | (0.030) | (0.037) | (0.016) | (0.024) |
| Cognitive PGI Father | 0.0187 | -0.00170 | -0.0406 | -0.0227 | -0.00501 | 0.0251 | 0.0131 |
| | (0.027) | (0.029) | (0.031) | (0.031) | (0.038) | (0.017) | (0.025) |
| Noncognitive PGI Father | -0.00797 | -0.00656 | 0.0220 | 0.000600 | 0.0275 | 0.0353* | 0.00509 |
| | (0.027) | (0.028) | (0.030) | (0.031) | (0.036) | (0.016) | (0.026) |
| Noncognitive PGI Mother | -0.00627 | 0.0186 | 0.0152 | 0.0246 | 0.0329 | -0.00680 | -0.0159 |
| | (0.026) | (0.028) | (0.030) | (0.029) | (0.038) | (0.016) | (0.025) |
| Observations | 1861 | 1869 | 2022 | 2051 | 1254 | 1500 | 576 |
| Adjusted R2 | 0.0563 | 0.0482 | 0.0589 | 0.0473 | 0.111 | 0.135 | 0.0797 |

Note: Robust standard errors in parentheses. Two-tailed t-test: $^{+}$ $p < 0.10$, $^{*}$ $p < 0.05$, $^{**}$ $p < 0.01$, $^{***}$ $p < 0.000$. Controls included but not reported above: first 10 PCs and Platform.

Table A12: OLS and LPM (academic tracking and educational attainment) regressions to test the interaction between children's PGI for cognitive and noncognitive skills and family SES on educational outcomes.

| | Mathematics (age 7) | Reading (age 7) | Mathematics (age 10) | Reading (age 10) | Test scores CITO (age 12) | Upper secondary track (age 12-18) | Educational Attainment (age ≥ 25) |
|---|---|---|---|---|---|---|---|
| SES x PGS Cognitive | -0.0662 | -0.0538 | -0.175* | -0.0705 | -0.102 | -0.0762* | -0.0558 |
| | (0.071) | (0.082) | (0.080) | (0.080) | (0.094) | (0.036) | (0.053) |
| Observations | 1861 | 1869 | 2022 | 2051 | 1254 | 1500 | 576 |
| Adjusted R2 | 0.0643 | 0.0521 | 0.0669 | 0.0456 | 0.112 | 0.131 | 0.0930 |
| SES x PGS Noncognitive | -0.0568 | -0.121 | -0.0339 | -0.0793 | -0.228* | -0.0770* | -0.105+ |
| | (0.072) | (0.082) | (0.078) | (0.079) | (0.093) | (0.039) | (0.056) |
| Observations | 1861 | 1869 | 2022 | 2051 | 1254 | 1500 | 576 |
| Adjusted R2 | 0.0634 | 0.0498 | 0.0604 | 0.0487 | 0.106 | 0.129 | 0.0677 |

Note: Robust standard errors in parentheses. Two-tailed t-test: $^{+}$ $p < 0.10$, $^{*}$ $p < 0.05$, $^{**}$ $p < 0.01$, $^{***}$ $p < 0.000$. Controls included but not reported above: first 10 PCs and Platform. We also include covariates-environment (family's' SES) and covariates-gene (PGI) interaction (Keller, 2014).

## 5. Power analysis

### 5.1. Power analysis for an R2 test in a multiple linear regression

We used the 'power rsquared' command in STATA to estimate the minimum incremental R2 that would yield a statistically significant result using an F-test. This command allows us to determine the smallest detectable increase in R2 given a predefined sample size and a specified power level of 0.8. For each outcome and design, we defined the R2 of the reduced model as 0.05 and the number of control variables as 11 before estimating the minimum incremental R2 required. The 'power rsquared' command then calculated the minimum detectable incremental R2, representing the difference between the R2 of the full model (R2_F) and that of the reduced model (R2_R).

In the between-family analysis, the minimum detectable value for the R2 difference ranges from 0.002 to 0.006, varying by sample. To achieve a statistical power of 80% and a significance level of 5%, our sample sizes enable us to detect incremental R2 associations of 0.20% in the mathematics and reading samples at ages 7 and 10, 0.30% in the CITO sample, 0.20% in tracking sample, and 0.60% in the educational attainment sample (see Table A13 and Figure A1). We conclude that by including an additional tested covariate (i.e., the interaction) in each sample, assuming a power of 80%, it is possible to confidently observe a significant change in the model's explanatory power.

Table A13: Estimated R-squared for multiple linear regression in the between-family analysis

| Outcome | Alpha | Power | N | R2_R | R2_F | Difference of R2 between full and reduced models | Number of tested covariates | Number of control covariates |
|---|---|---|---|---|---|---|---|---|
| Reading age 7 | .05 | .8 | 3,728 | .05 | 0.052 | 0.0020 | 1 | 11 |
| Mathematics age 7 | .05 | .8 | 3,756 | .05 | 0.052 | 0.0020 | 1 | 11 |
| Reading age 10 | .05 | .8 | 3,829 | .05 | 0.052 | 0.0019 | 1 | 11 |
| Mathematics age 10 | .05 | .8 | 3,875 | .05 | 0.052 | 0.0019 | 1 | 11 |
| Test score (CITO) | .05 | .8 | 2,690 | .05 | 0.053 | 0.0027 | 1 | 11 |
| Upper secondary track | .05 | .8 | 3,318 | .05 | 0.052 | 0.0022 | 1 | 11 |
| Educational attainment | .05 | .8 | 1,224 | .05 | 0.056 | 0.0060 | 1 | 11 |

Note: R2_F: R2 of the full model, R2_R: R2 of the reduced model.

Figure A1: Estimated R-squared for multiple linear regression in the between-family analysis

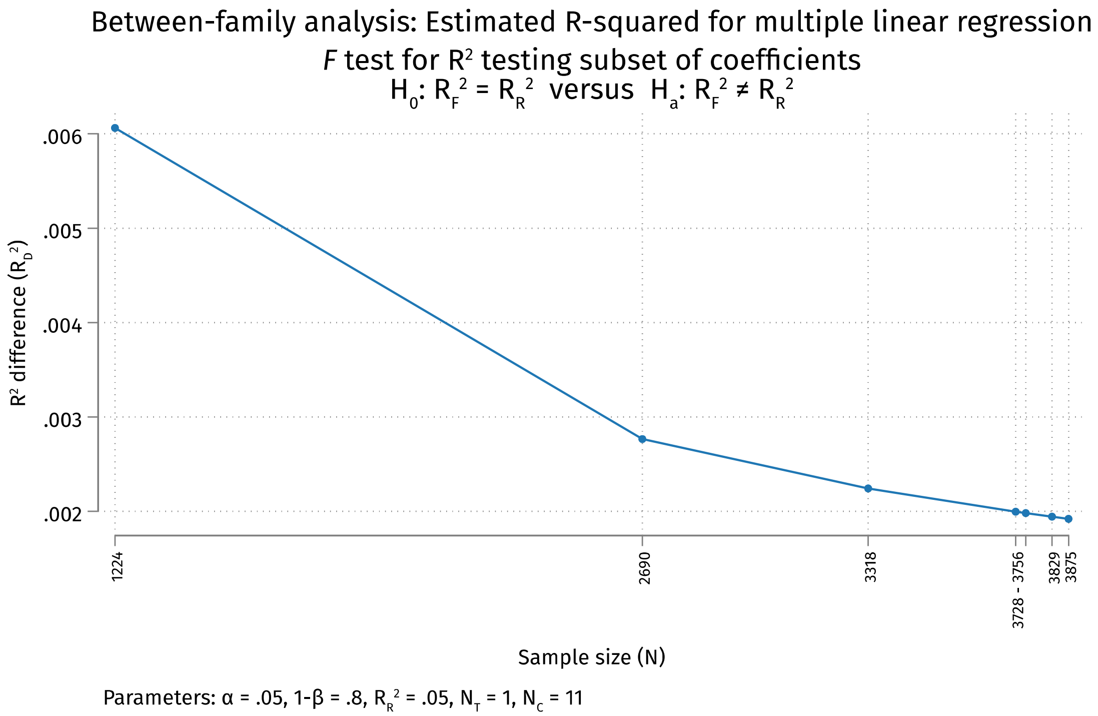


In the within-family analysis, the minimum detectable value for the R2 difference ranges from 0.003 to 0.013. Therefore, if we require a statistical power of 80% and a significance level of 5%, our sample sizes will allow us to detect associations with an incremental R2 of 0.35% in the mathematics and reading sample at age 7, 0.33% in the mathematics and reading sample at age 10, 0.50% in the CITO sample, 0.37% in secondary tracking sample, and 1.17% in the educational attainment sample (see Table A14 and Figure A2). Again, the minimum detectable R2 is small – except for educational attainment – which suggests that including the interaction coefficient in the model allows capturing really small changes in the explanatory power of the model.

Table A14: Estimated R-squared for multiple linear regression in the within-family analysis

| Outcome | Alpha | Power | N | R2_R | R2_F | Difference of R2 between full and reduced models | Number of tested covariates | Number of control covariates |
|---|---|---|---|---|---|---|---|---|
| Reading age 7 | .05 | .8 | 2,124 | .05 | 0.053 | 0.0035 | 1 | 11 |
| Mathematics age 7 | .05 | .8 | 2,130 | .05 | 0.053 | 0.0035 | 1 | 11 |
| Reading age 10 | .05 | .8 | 2,212 | .05 | 0.053 | 0.0033 | 1 | 11 |
| Mathematics age 10 | .05 | .8 | 2,236 | .05 | 0.053 | 0.0033 | 1 | 11 |
| Test score (CITO) | .05 | .8 | 1,500 | .05 | 0.055 | 0.0049 | 1 | 11 |
| Upper secondary track | .05 | .8 | 2,004 | .05 | 0.054 | 0.0037 | 1 | 11 |
| Educational attainment | .05 | .8 | 426 | .05 | 0.067 | 0.0172 | 1 | 11 |

Note: R2_F: R2 of the full model, R2_R: R2 of the reduced model.

Figure A2: Estimated R-squared for multiple linear regression in the within-family analysis

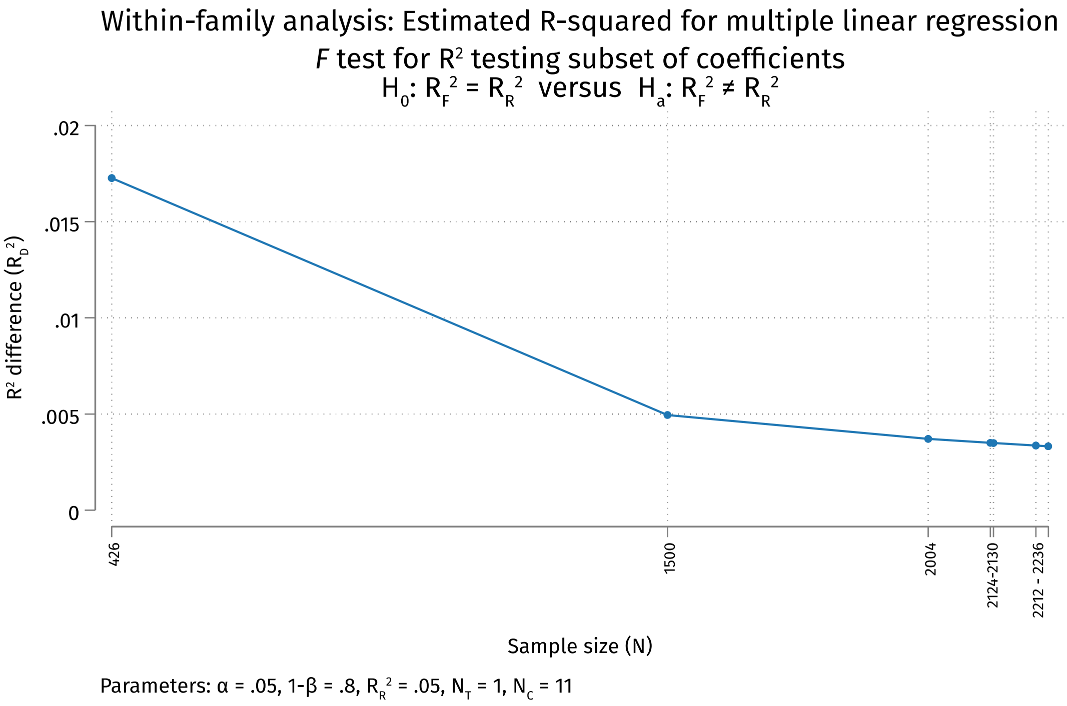


Finally, in the trio analysis, the minimum detectable value for the R2 difference ranges from 0.004 to 0.013 (see Table A15 and Figure A3). This means that if we require a statistical power of 80% and a significance level of 5%, our sample sizes will allow us to detect associations with an incremental R2 of 0.40% in the mathematics and reading sample at age 7, 0.36% in the mathematics and reading sample at age 10, 0.60% in the CITO sample, 0.50% in the tracking sample and 1.3% in the educational attainment sample. In line with the within-family analysis, the minimum detectable R2 is generally small – except for educational attainment. Thus, including the interaction coefficient in the model enables the detection of even minor variations in the model's explanatory power.

Table A15: Estimated R-squared for multiple linear regression in the trio analysis

| Outcome | Alpha | Power | N | R2_R | R2_F | Difference of R2 between full and reduced models | Number of tested covariates | Number of control covariates |
|---|---|---|---|---|---|---|---|---|
| Reading age 7 | .05 | .8 | 1,861 | .05 | 0.054 | .0039 | 1 | 11 |
| Mathematics age 7 | .05 | .8 | 1,869 | .05 | 0.054 | .0039 | 1 | 11 |
| Reading age 10 | .05 | .8 | 2,022 | .05 | 0.054 | .0036 | 1 | 11 |
| Mathematics age 10 | .05 | .8 | 2,051 | .05 | 0.054 | .0036 | 1 | 11 |
| Test score (CITO) | .05 | .8 | 1,254 | .05 | 0.056 | .0059 | 1 | 11 |
| Upper secondary track | .05 | .8 | 1,500 | .05 | 0.055 | .0049 | 1 | 11 |
| Educational attainment | .05 | .8 | 576 | .05 | 0.063 | .0128 | 1 | 11 |

Note: R2_F: R2 of the full model, R2_R: R2 of the reduced model.

Figure A3: Estimated R-squared for multiple linear regression in the trio analysis

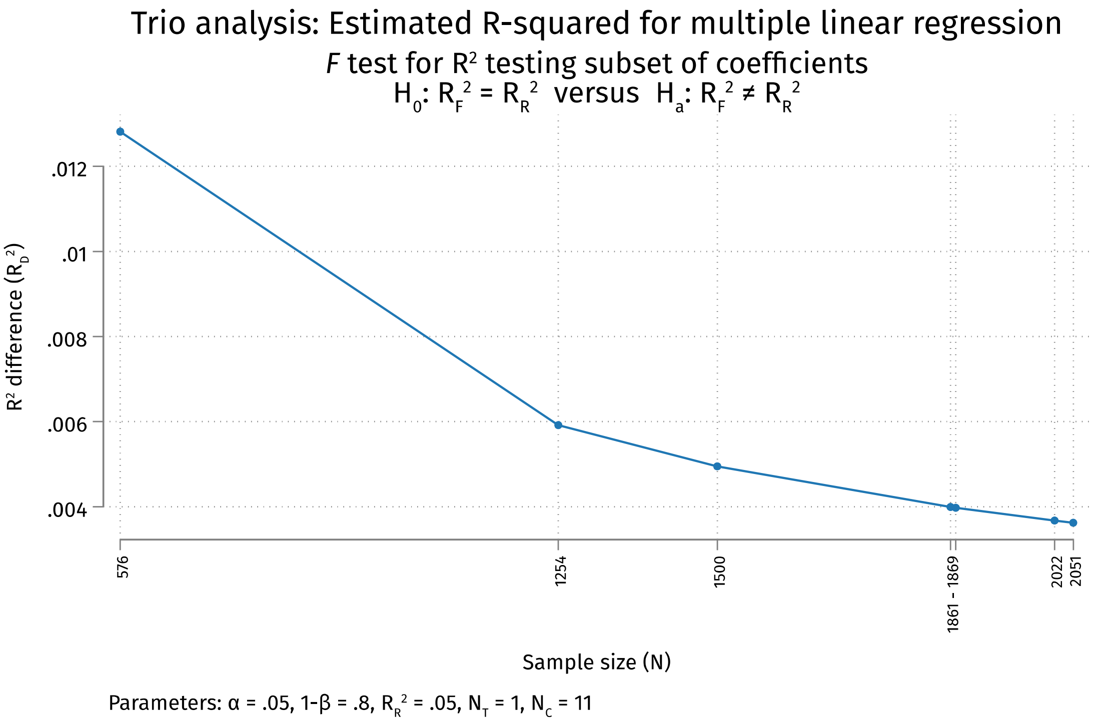


## 5.2. Post-hoc power analysis using Monte Carlo simulation

We also conduct a post-hoc power analysis using Monte Carlo simulations. Power estimates are derived from 1,000 Monte Carlo simulations with alpha set to 0.05 for each outcome in each design (between, within and trio). Statistical power is the probability of detecting a significant result given that the alternative (here G×E) hypothesis is true. We implement the following procedure:

1. We randomly generate outcome variables using our original model. Precisely,
    a. We use all parameter values from our original model, i.e. based on the assumption that the alternative hypothesis is true (all betas are different from zero).
    b. Under the assumption that the errors are normally distributed, we include the error term as a random draw for each observation.
2. We run our regression model on these randomly generated outcomes and obtain the p-value for the GxE coefficient.
3. We save the p-value of the test and obtain the test result ("reject" or "fail to reject"). We reject the null hypothesis when the p-value is below our significance level alpha (0.05).
4. We repeat the above steps 1000 times.
5. Finally, we look at the proportion of times that the null hypothesis is rejected (out of 1000). This proportion is our estimate of power.

### 5.2.1. Assumptions

1. Our model is the true model
2. Errors are normally distributed

### 5.2.2. Results

The power analysis indicates a potential issue with low statistical power in our study, particularly affecting certain outcomes such as grades, the within-family design, and analyses using the noncognitive PGI. However, in the between-family analysis, the power analysis confirms sufficient statistical power for the outcomes where statistically significant results were observed (see Tables A16 and A17). These outcomes, which exhibited higher statistical power, are tracking and educational attainment. Moreover, the statistical power is also higher for CITO and mathematics at age 10 than for other outcomes. This highlights the robustness of our findings in detecting those statistically significant interactions.

Table A16: Statistical power using the PGI for cognitive skills

| | Mathematics age 7 | Reading age 7 | Mathematics age 10 | Reading age 10 | Testo score (CITO) | Upper secondary track | Educational attainment |
|---|---|---|---|---|---|---|---|
| Between-family | 21% | 7% | 46% | 6% | 42% | 85% | 87% |
| Within-family | 6% | 13% | 11% | 7% | 17% | 17% | 14% |
| Trio | 9% | 13% | 56% | 11% | 17% | 39% | 19% |

Table A17: Statistical power using the PGI for noncognitive skills

| | Mathematics age 7 | Reading age 7 | Mathematics age 10 | Reading age 10 | Testo score (CITO) | Upper secondary track | Educational attainment |
|---|---|---|---|---|---|---|---|
| Between-family | 6% | 6% | 9% | 8% | 23% | 63% | 80% |
| Within-family | 6% | 25% | 8% | 5% | 6% | 12% | 12% |
| Trio | 6% | 33% | 7% | 19% | 57% | 43% | 54% |

### 5.2.3. Replicability

We created a STATA program (see ados: powersimuB and powersimuWI) and then ran the program to conduct the power analysis using Monte Carlo simulation (see dofiles: 9_power_analysis_PGICOG and 9_power_analysis_PGINCOG in the replication package). See also https://www.stata.com/support/faqs/statistics/power-by-simulation/

## 6. Multiple testing

Since the large number of outcomes included in this paper (seven in total), we have applied formal corrections to the p-values of our results to account for multiple comparisons. We used two procedures. First, we apply Bonferroni correction. For each PGI, we performed the same analysis on 7 outcomes, so the adjusted p-value threshold for significance is $0.05/7 = 0.007$. Then, we implement the bootstrap procedure for multiple testing corrections introduced by Romano and Wolf (2005), further detailed in Romano and Wolf (2016) and Clarke et al. (2020). Specifically, the Romano-Wolf correction controls the family-wise error rate (FWER), which is the probability of incorrectly rejecting at least one true null hypothesis in a family of hypotheses under investigation. This method yields adjusted p-values robust against inflated Type I error rates and accommodates the dependence structure among the test statistics.

Tables A18-A19 present the original p-values and Romano-Wolf corrected p-values for each test. They also indicate whether the outcome p-values are under the Bonferroni threshold for the interaction between family SES and the PGI for cognitive skills (Table A18) or noncognitive skills (Table A19). Overall, 7 out of 10 statistically significant negative interactions survive the Romano-Wolf correction (4 for the cognitive PGI and 3 for the noncognitive PGI), while the only significant positive interaction in the within-family analysis does not. Since Bonferroni is a more conservative approach, we only find robust evidence for a negative GxE interaction in the between-family design for tracking (cognitive skills' PGI) and educational attainment (cognitive and noncognitive skills' PGIs)

Table A18: Corrections for multiple hypothesis testing by outcome and design for the interaction between family SES and PGI for cognitive skills

| | *Between-family analysis* | | | *Within-family analysis* | | | *Trio analysis* | | |
|---|---|---|---|---|---|---|---|---|---|
| **Outcome** | **Original p-value** | **Romano-Wolf p-value** | **Under the Bonferroni p-value threshold** | **Original p-value** | **Romano-Wolf p-value** | **Under the Bonferroni p-value threshold** | **Original p-value** | **Romano-Wolf p-value** | **Under the Bonferroni p-value threshold** |
| Mathematics (age 7) | 0.247 | 0.263 | No | 0.747 | 0.962 | No | 0.350 | 0.504 | No |
| Reading (age 7) | 0.729 | 0.848 | No | 0.232 | 0.697 | No | 0.510 | 0.504 | No |
| Mathematics (age 10) | 0.081 | 0.069 | No | 0.046 | 0.298 | No | 0.029 | 0.029 | No |
| Reading (age 10) | 0.884 | 0.852 | No | 0.633 | 0.962 | No | 0.379 | 0.504 | No |
| Test score (CITO) | 0.059 | 0.050 | No | 0.109 | 0.478 | No | 0.279 | 0.504 | No |
| Upper secondary track | 0.001 | 0.001 | Yes | 0.036 | 0.278 | No | 0.035 | 0.031 | No |
| Educational attainment | 0.000 | 0.001 | Yes | 0.661 | 0.962 | No | 0.264 | 0.504 | No |

Note: we used the STATA command "rwolf2" to compute the Romano-Wolf corrected p-values resulting from 1,000 bootstrap replications.

Table A19: Corrections for multiple hypothesis testing by outcome and design for the interaction between family SES and PGI for noncognitive skills

| | *Between-family analysis* | | | *Within-family analysis* | | | *Trio analysis* | | |
|---|---|---|---|---|---|---|---|---|---|
| **Outcome** | **Original p-value** | **Romano-Wolf p-value** | **Under the Bonferroni p-value threshold** | **Original p-value** | **Romano-Wolf p-value** | **Under the Bonferroni p-value threshold** | **Original p-value** | **Romano-Wolf p-value** | **Under the Bonferroni p-value threshold** |
| Mathematics (age 7) | 0.775 | 0.911 | No | 0.464 | 0.877 | No | 0.433 | 0.482 | No |
| Reading (age 7) | 0.833 | 0.911 | No | 0.045 | 0.342 | No | 0.138 | 0.166 | No |
| Mathematics (age 10) | 0.632 | 0.911 | No | 0.223 | 0.803 | No | 0.666 | 0.558 | No |
| Reading (age 10) | 0.664 | 0.911 | No | 0.832 | 0.964 | No | 0.317 | 0.419 | No |
| Test score (CITO) | 0.184 | 0.254 | No | 0.867 | 0.964 | No | 0.014 | 0.012 | No |
| Upper secondary track | 0.012 | 0.030 | No | 0.342 | 0.877 | No | 0.048 | 0.067 | No |
| Educational attainment | 0.000 | 0.001 | Yes | 0.319 | 0.877 | No | 0.068 | 0.067 | No |

Note: we used the STATA command "rwolf2" to compute the Romano-Wolf corrected p-values resulting from 1,000 bootstrap replications.

## 7. Robustness checks

## 7.1. Alternative control variables

### 7.1.1 Between-family design

Table A20: OLS and LPM (academic tracking and educational attainment) regressions to test the interaction between children's cognitive and noncognitive PGI and family SES on educational outcomes controlling also for gender and birth year.

| | Mathematics (age 7) | Reading (age 7) | Mathematics (age 10) | Reading (age 10) | Test scores CITO (age 12) | Upper secondary track (age 12-18) | Educational Attainment (age ≥ 25) |
|---|---|---|---|---|---|---|---|
| SES x PGI Cognitive | -0.0417 | -0.00967 | -0.0721$^{+}$ | 0.00969 | -0.0878$^{+}$ | -0.0643$^{***}$ | -0.112$^{***}$ |
| | (0.037) | (0.041) | (0.041) | (0.039) | (0.048) | (0.019) | (0.028) |
| Observations | 3728 | 3756 | 3829 | 3875 | 2690 | 3318 | 1224 |
| Adjusted R2 | 0.0604 | 0.0535 | 0.0831 | 0.0554 | 0.134 | 0.119 | 0.0994 |
| SES x PGI Noncognitive | -0.0111 | -0.00798 | -0.0266 | -0.0150 | -0.0569 | -0.0485$^{*}$ | -0.0980$^{***}$ |
| | (0.038) | (0.041) | (0.038) | (0.040) | (0.044) | (0.019) | (0.029) |
| Observations | 3728 | 3756 | 3829 | 3875 | 2690 | 3318 | 1224 |
| Adjusted R2 | 0.0600 | 0.0516 | 0.0819 | 0.0562 | 0.134 | 0.121 | 0.103 |

Note: Robust standard errors in parentheses. Two-tailed t-test: $^{+}$ $p < 0.10$, $^{*}$ $p < 0.05$, $^{**}$ $p < 0.01$, $^{***}$ $p < 0.000$. Controls included but not reported above: first 10 PCs, Platform, gender and birth year. We also include covariates-environment (family's' SES) and covariates-gene (PGI) interaction (Keller, 2014).

### 7.1.2 Within-family design

Table A21: Family-fixed effect regressions to test the interaction between children's cognitive and noncognitive PGI and family SES on educational outcomes controlling also for gender and birth year.

| | Mathematics (age 7) | Reading (age 7) | Mathematics (age 10) | Reading (age 10) | Test scores CITO (age 12) | Upper secondary track (age 12-18) | Educational Attainment (age ≥ 25) |
|---|---|---|---|---|---|---|---|
| | (0.032) | (0.035) | (0.037) | (0.037) | (0.048) | (0.021) | (0.055) |
| SES x PGS Cognitive | -0.0113 | -0.102 | -0.161$^{+}$ | -0.0510 | -0.135 | -0.0804$^{*}$ | -0.0588 |
| | (0.088) | (0.089) | (0.086) | (0.091) | (0.087) | (0.037) | (0.100) |
| Observations | 2124 | 2130 | 2212 | 2236 | 1500 | 2004 | 426 |
| Adjusted R2 | 0.0683 | 0.0435 | 0.108 | 0.0455 | 0.0678 | 0.0465 | 0.130 |
| SES x PGS Noncognitive | -0.0389 | 0.167$^{+}$ | -0.112 | 0.0197 | 0.0134 | -0.0406 | -0.0700 |
| | (0.089) | (0.085) | (0.092) | (0.099) | (0.098) | (0.043) | (0.086) |
| Observations | 2124 | 2130 | 2212 | 2236 | 1500 | 2004 | 426 |
| Adjusted R2 | 0.0592 | 0.0466 | 0.0964 | 0.0421 | 0.0610 | 0.0517 | 0.159 |

Note: Robust standard errors in parentheses. Two-tailed t-test: $^{+}$ $p < 0.10$, $^{*}$ $p < 0.05$, $^{**}$ $p < 0.01$, $^{***}$ $p < 0.000$. Controls included but not reported above: first 10 PCs, Platform, gender and birth year. We also include covariates-environment (family's' SES) and covariates-gene (PGI) interaction (Keller, 2014).

### 7.1.3 Trio design

Table A22: OLS and LPM (academic tracking and educational attainment) regressions to test the interaction between children's PGI for cognitive and noncognitive skills and family SES on educational outcomes controlling also for gender and birth year.

| | Mathematics (age 7) | Reading (age 7) | Mathematics (age 10) | Reading (age 10) | Test scores CITO (age 12) | Upper secondary track (age 12-18) | Educational Attainment (age ≥ 25) |
|---|---|---|---|---|---|---|---|
| SES x PGS Cognitive | -0.0418 | -0.0542 | -0.163* | -0.0580 | -0.0949 | -0.0765* | -0.0558 |
| | (0.070) | (0.082) | (0.078) | (0.081) | (0.095) | (0.036) | (0.053) |
| Observations | 1861 | 1869 | 2022 | 2051 | 1254 | 1500 | 576 |
| Adjusted R2 | 0.0724 | 0.0591 | 0.0890 | 0.0534 | 0.113 | 0.129 | 0.0930 |
| SES x PGS Noncognitive | -0.0309 | -0.119 | -0.0413 | -0.0794 | -0.225* | -0.0764* | -0.102+ |
| | (0.073) | (0.082) | (0.079) | (0.080) | (0.094) | (0.038) | (0.056) |
| Observations | 1861 | 1869 | 2022 | 2051 | 1254 | 1500 | 576 |
| Adjusted R2 | 0.0711 | 0.0559 | 0.0828 | 0.0567 | 0.107 | 0.127 | 0.0677 |

Note: Robust standard errors in parentheses. Two-tailed t-test: $^{+}$ $p < 0.10$, $^{*}$ $p < 0.05$, $^{**}$ $p < 0.01$, $^{***}$ $p < 0.000$. Controls included but not reported above: first 10 PCs, Platform, gender and birth year. We also include covariates-environment (family's' SES) and covariates-gene (PGI) interaction (Keller, 2014).

## 7.2. Alternative model specifications

## 7.2.1. Logistic regression models

### 7.2.1.1 Between-family design

Figure A4: Logistic regression models for dichotomous outcomes variables in the *between-family samples* (without controlling for parents' PGI) for the interaction between family's SES and PGI for cognitive skills (average marginal effect, at 95 percent confidence interval)

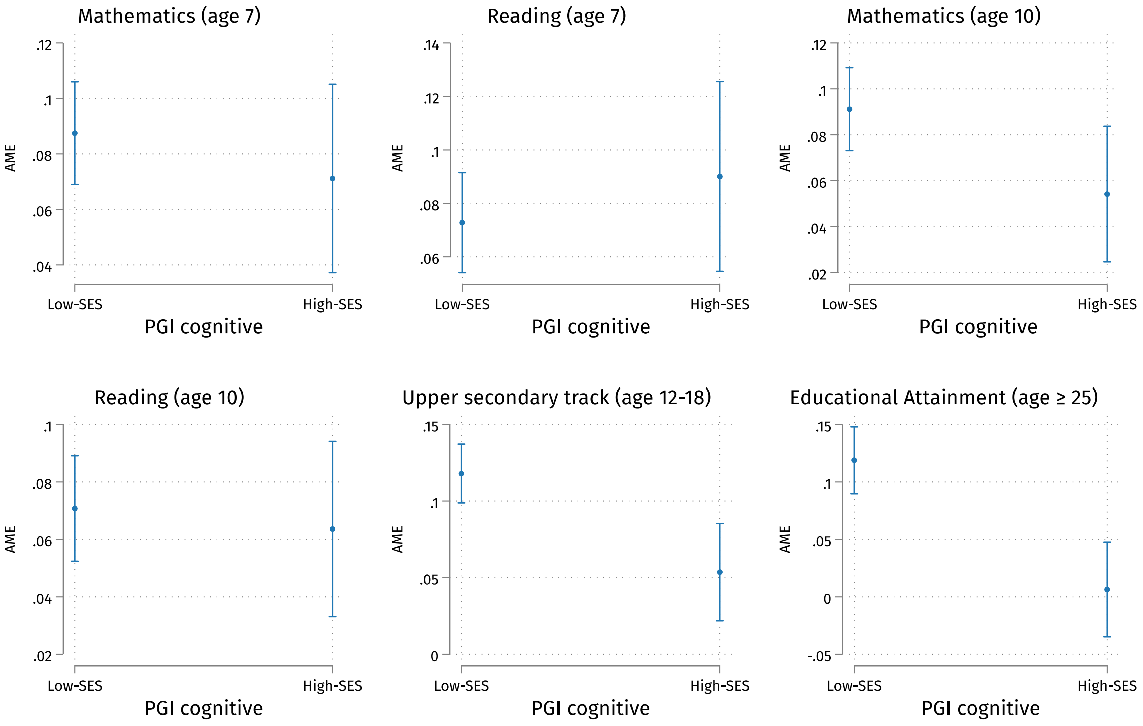


Note: Average marginal effect (AME). Controls are included. We also include covariates-environment (family SES) and covariates-gene (PGI) interaction (Keller, 2014). For this robustness check, school grades are dichotomized.

Figure A5: Logistic regression models for dichotomous outcomes variables in the *between-family samples* (without controlling for parents' PGI) for the interaction between family's SES and PGI for noncognitive skills (average marginal effect, at 95 percent confidence interval)

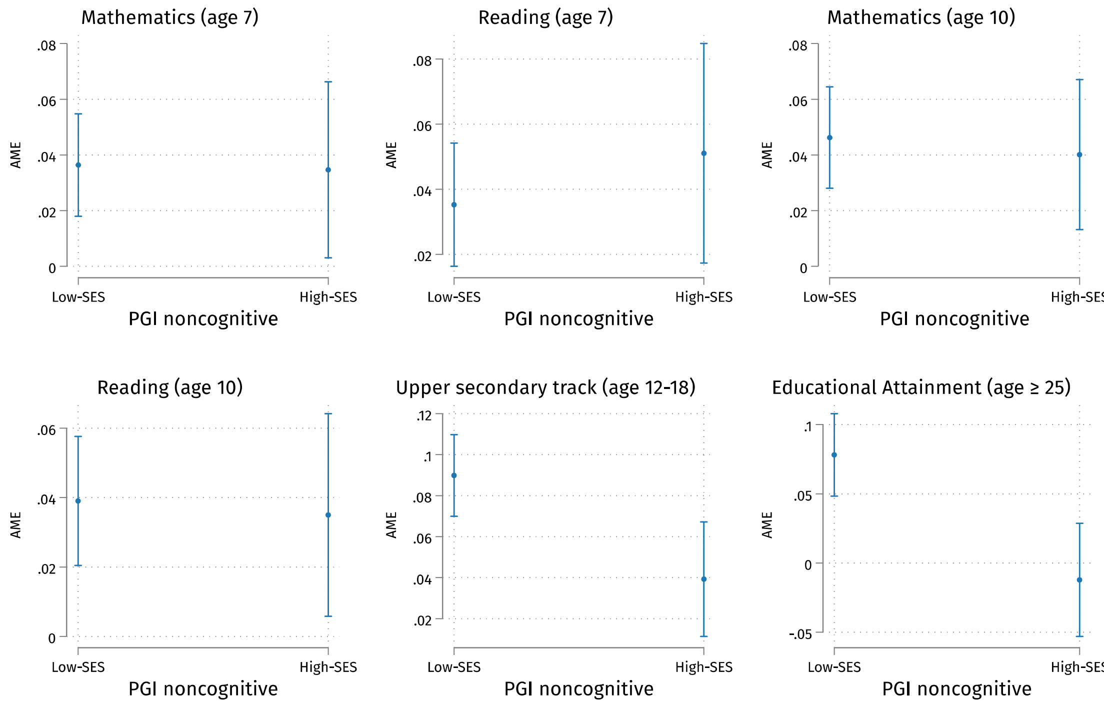


Note: Average marginal effect (AME). Controls are included. We also include covariates-environment (family SES) and covariates-gene (PGI) interaction (Keller, 2014). For this robustness check, school grades are dichotomized.

### 7.2.1.2 Within-family design

Table A23: Logistic regression models for dichotomous outcomes variables in the *within-samples* for the interaction between family SES and PGI for cognitive and noncognitive skills at 95 percent confidence interval

| | Mathematics (age 7) | Reading (age 7) | Mathematics (age 10) | Reading (age 10) | Upper secondary track (age 12-18) | Educational Attainment (age ≥ 25) |
|---|---|---|---|---|---|---|
| High-SES x Cognitive PGI | -0.303 | -0.473 | -0.550$^{+}$ | -0.278 | -0.427 | 1.914 |
| | (0.328) | (0.315) | (0.322) | (0.316) | (0.471) | (1.928) |
| High-SES x NonCognitive PGI | -0.217 | 0.740$^{*}$ | -0.418 | -0.0284 | 0.0665 | -0.941 |
| | (0.352) | (0.374) | (0.299) | (0.287) | (0.430) | (0.791) |
| Observations | 552 | 600 | 608 | 662 | 554 | 136 |

Note: Standard errors in parentheses. $^{+}$ $p < 0.10$, $^{*}$ $p < 0.05$, $^{**}$ $p < 0.01$, $^{***}$ $p < 0.001$. Controls are included. We also include covariates-environment (family SES) and covariates-gene (PGI) interaction (Keller, 2014). For this robustness check, school grades are dichotomized. Scale: log odds.

### 7.2.1.3 Trio design

Figure A6: Logistic regression models for dichotomous outcomes variables in the *trio samples* (controlling for parents' PGI) for the interaction between family SES and PGI for cognitive skills (average marginal effect, at 95 percent confidence interval)

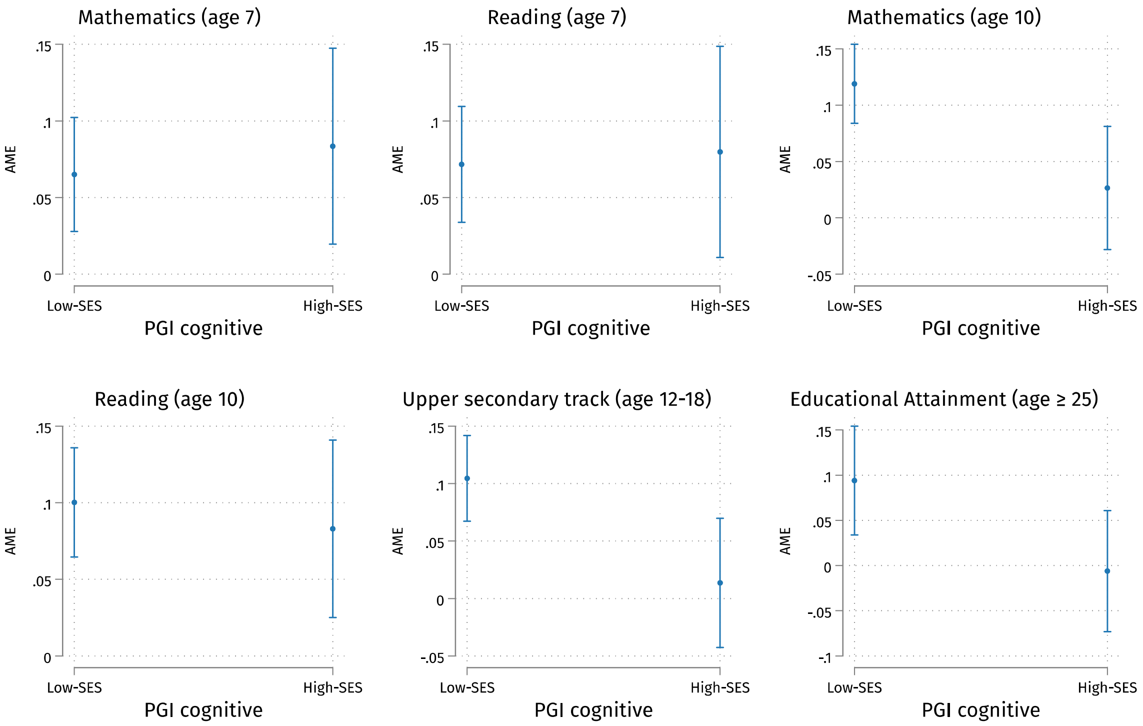


Note: Average marginal effect (AME). Controls are included. We also include covariates-environment (family SES) and covariates-gene (PGI) interaction (Keller, 2014). For this robustness check, school grades are dichotomised.

Figure A7: Logistic regression models for dichotomous outcomes variables in the *trio samples* (controlling for parents' PGI) for the interaction between family's SES and PGI for noncognitive skills (average marginal effect, at 95 percent confidence interval)

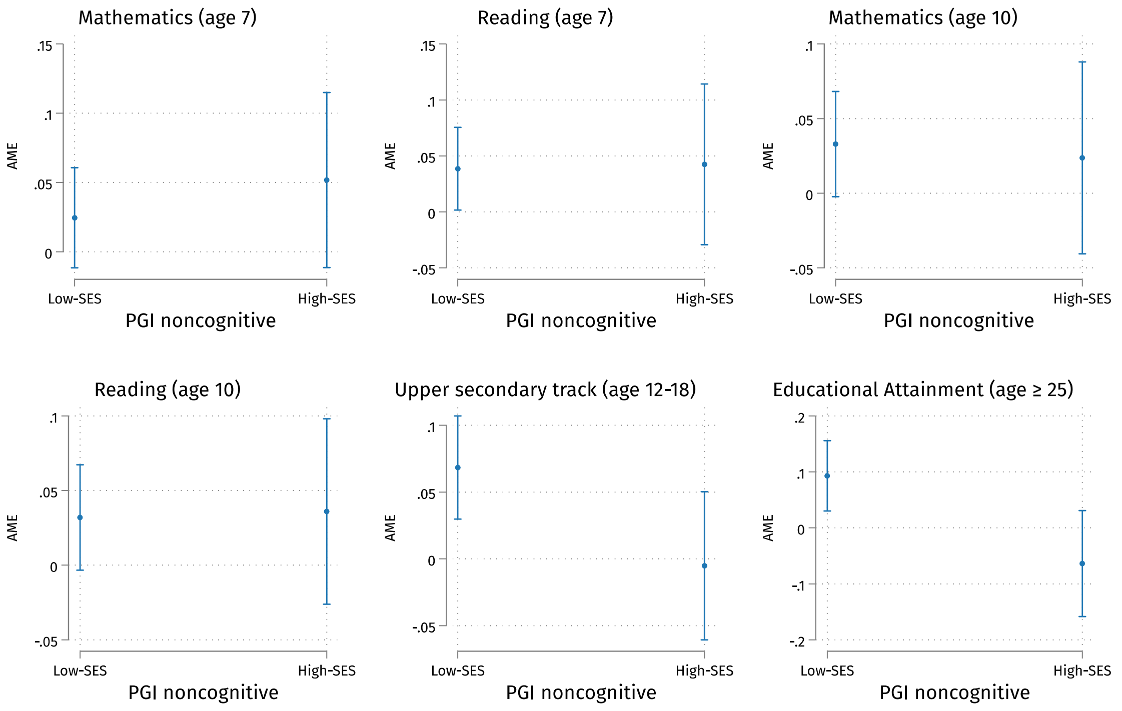


Note: Average marginal effect (AME). Controls are included. We also include covariates-environment (family SES) and covariates-gene (PGI) interaction (Keller, 2014). For this robustness check, school grades are dichotomised.

### 7.2.2. Non-linear PGIs models for continuous outcomes

### 7.2.2.1 Between-family design

Table A24: OLS regression models with PGI in terciles in the *between-family samples* (without controlling for parents' PGI) for the interaction between family's SES and PGI for cognitive skills on our not dichotomous variables (i.e., CITO and school grades).

| | Mathematics (age 7) | Reading (age 7) | Mathematics (age 10) | Reading (age 10) | Test scores CITO (age 12) |
|---|---|---|---|---|---|
| SES x PGI Cognitive (2nd tercile) | 0.00616 | 0.000604 | -0.164 | -0.152 | 0.00456 |
| | (0.096) | (0.100) | (0.103) | (0.097) | (0.122) |
| SES x PGI Cognitive (3rd tercile) | -0.0170 | 0.0479 | -0.126 | 0.0194 | -0.203 |
| | (0.094) | (0.102) | (0.104) | (0.100) | (0.127) |
| Observations | 3728 | 3756 | 3829 | 3875 | 2690 |
| Adjusted R2 | 0.0458 | 0.0385 | 0.0594 | 0.0419 | 0.115 |

Note: Robust standard errors in parentheses. Two-tailed t-test: $^{+}\ p < 0.10$, $^{*}\ p < 0.05$, $^{**}\ p < 0.01$, $^{***}\ p < 0.000$. Controls included but not reported above: first 10 PCs and Platform. We also include covariates-environment (family SES) and covariates-gene (PGI) interaction (Keller, 2014).

Figure A8: OLS regression models with PGI in terciles in the *between-family samples* (without controlling for parents' PGI) for the interaction between family's SES and PGI for cognitive skills on our not dichotomous variables (i.e., CITO and school grades).

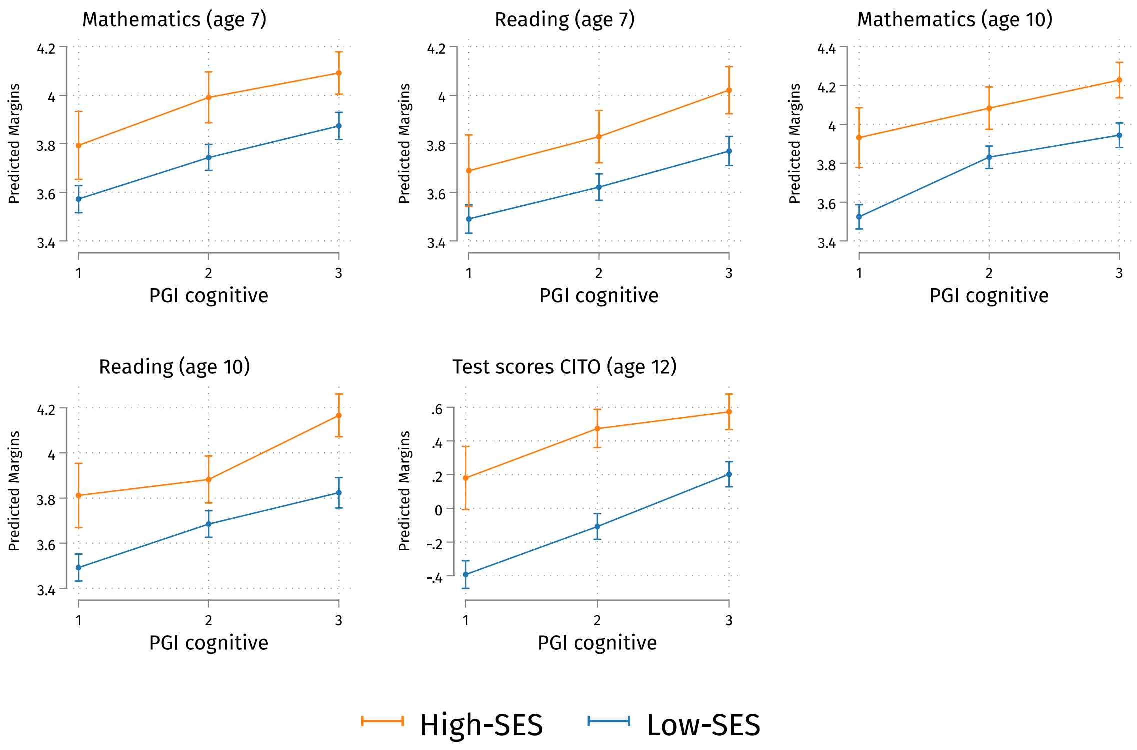


Controls are included. We also include covariates-environment (family SES) and covariates-gene (PGI) interaction (Keller, 2014).

Table A25: OLS regression models with PGI in terciles in the *between-family samples* (without controlling for parents' PGI) for the interaction between family's SES and PGI for noncognitive skills on our not dichotomous variables (i.e., CITO and school grades).

| | Mathematics (age 7) | Reading (age 7) | Mathematics (age 10) | Reading (age 10) | Test scores CITO (age 12) |
|---|---|---|---|---|---|
| SES x PGI Non-Cognitive (2nd tercile) | 0.0306 | 0.0854 | -0.0459 | 0.0433 | -0.139 |
| | (0.096) | (0.105) | (0.093) | (0.099) | (0.115) |
| SES x PGI Non-Cognitive (3rd tercile) | -0.0320 | -0.0271 | -0.0798 | -0.0691 | -0.0886 |
| | (0.093) | (0.100) | (0.095) | (0.095) | (0.105) |
| Observations | 3728 | 3756 | 3829 | 3875 | 2690 |
| Adjusted R2 | 0.0404 | 0.0361 | 0.0569 | 0.0416 | 0.113 |

Note: Robust standard errors in parentheses. Two-tailed t-test: $^{+}$ $p < 0.10$, $^{*}$ $p < 0.05$, $^{**}$ $p < 0.01$, $^{***}$ $p < 0.000$. Controls included but not reported above: first 10 PCs and Platform. We also include covariates-environment (family SES) and covariates-gene (PGI) interaction (Keller, 2014).

Figure A9: OLS regression models with PGI in terciles in the *between-family samples* (without controlling for parents' PGI) for the interaction between family's SES and PGI for noncognitive skills on our not dichotomous variables (i.e., CITO and school grades).

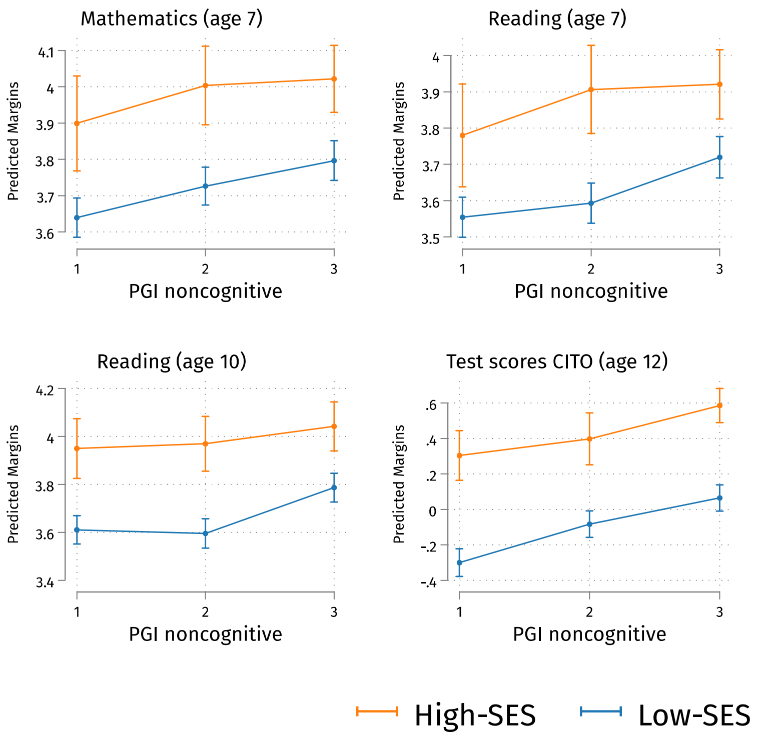


Controls are included. We also include covariates-environment (family SES) and covariates-gene (PGI) interaction (Keller, 2014).

### 7.2.2.2 Trio design

Table A26: OLS regression models with PGI in terciles in the *trio samples* for the interaction between family's SES and PGI for cognitive skills on our not dichotomous variables (i.e., CITO and school grades).

| | Mathematics (age 7) | Reading (age 7) | Mathematics (age 10) | Reading (age 10) | Test scores CITO (age 12) |
|---|---|---|---|---|---|
| SES x PGI Cognitive (2nd tercile) | -0.0177 | -0.0556 | -0.260$^{+}$ | -0.160 | -0.0735 |
| | (0.137) | (0.148) | (0.141) | (0.139) | (0.197) |
| SES x PGI Cognitive (3rd tercile) | -0.0805 | -0.109 | -0.258 | -0.0522 | -0.249 |
| | (0.162) | (0.173) | (0.173) | (0.169) | (0.230) |
| Observations | 1861 | 1869 | 2022 | 2051 | 1254 |
| Adjusted R2 | 0.0572 | 0.0496 | 0.0601 | 0.0450 | 0.106 |

Note: Robust standard errors in parentheses. Two-tailed t-test: $^{+}$ $p < 0.10$, $^{*}$ $p < 0.05$, $^{**}$ $p < 0.01$, $^{***}$ $p < 0.000$. Controls included but not reported above: first 10 PCs and Platform. We also include covariates-environment (family SES) and covariates-gene (PGI) interaction (Keller, 2014).

Figure A10: OLS regression models with PGI in terciles in the *trio samples* for the interaction between family's SES and PGI for cognitive skills on our not dichotomous variables (i.e., CITO and school grades).

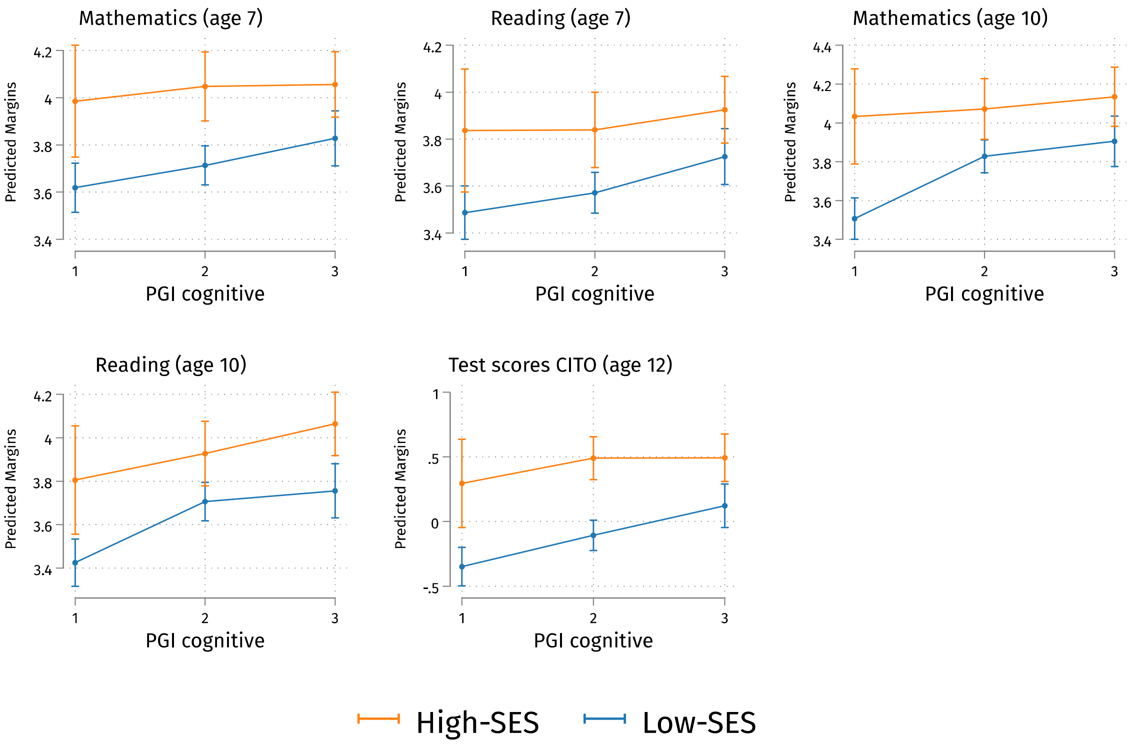


Controls are included. We also include covariates-environment (family's' SES) and covariates-gene (PGI) interaction (Keller, 2014).

Table A27: OLS regression models with PGI in terciles in the *trio samples* for the interaction between family's SES and PGI for non-cognitive skills on our not dichotomous variables (i.e., CITO and school grades).

| | Mathematics (age 7) | Reading (age 7) | Mathematics (age 10) | Reading (age 10) | Test scores CITO (age 12) |
|---|---|---|---|---|---|
| SES x PGI Non-Cognitive (2nd tercile) | -0.102 | -0.214 | -0.0522 | -0.104 | -0.272 |
| | (0.146) | (0.154) | (0.137) | (0.146) | (0.177) |
| SES x PGI Non-Cognitive (3rd tercile) | -0.194 | -0.414* | -0.169 | -0.256 | -0.376* |
| | (0.163) | (0.172) | (0.169) | (0.170) | (0.189) |
| Observations | 1861 | 1869 | 2022 | 2051 | 1254 |
| Adjusted R2 | 0.0577 | 0.0406 | 0.0518 | 0.0481 | 0.101 |

Note: Robust standard errors in parentheses. Two-tailed t-test: + $p < 0.10$, * $p < 0.05$, ** $p < 0.01$, *** $p < 0.000$. Controls included but not reported above: first 10 PCs and Platform. We also include covariates-environment (family SES) and covariates-gene (PGI) interaction (Keller, 2014).

Figure A11: OLS regression models with PGI in terciles in the *trio samples* for the interaction between family's SES and PGI for non-cognitive skills on our not dichotomous variables (i.e., CITO and school grades).

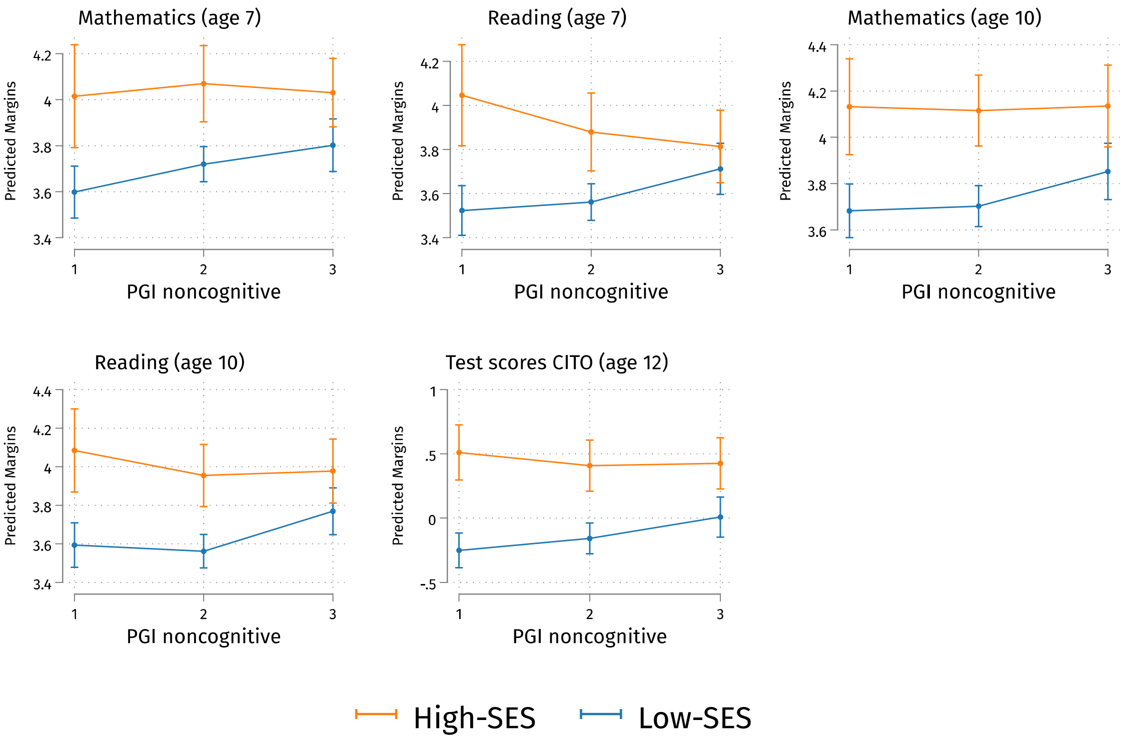


Controls are included. We also include covariates-environment (family SES) and covariates-gene (PGI) interaction (Keller, 2014).

### 7.2.3. Logistic regression models and non-linearities in the PGI

#### 7.2.3.1. Between-family design

Figure A12: Logistic regression models for dichotomous outcomes variables and with the PGI in terciles in the *between family samples* (without controlling for parents' PGI) for the interaction between family's SES and PGI for cognitive skills (average marginal effect, at 95 percent confidence interval)

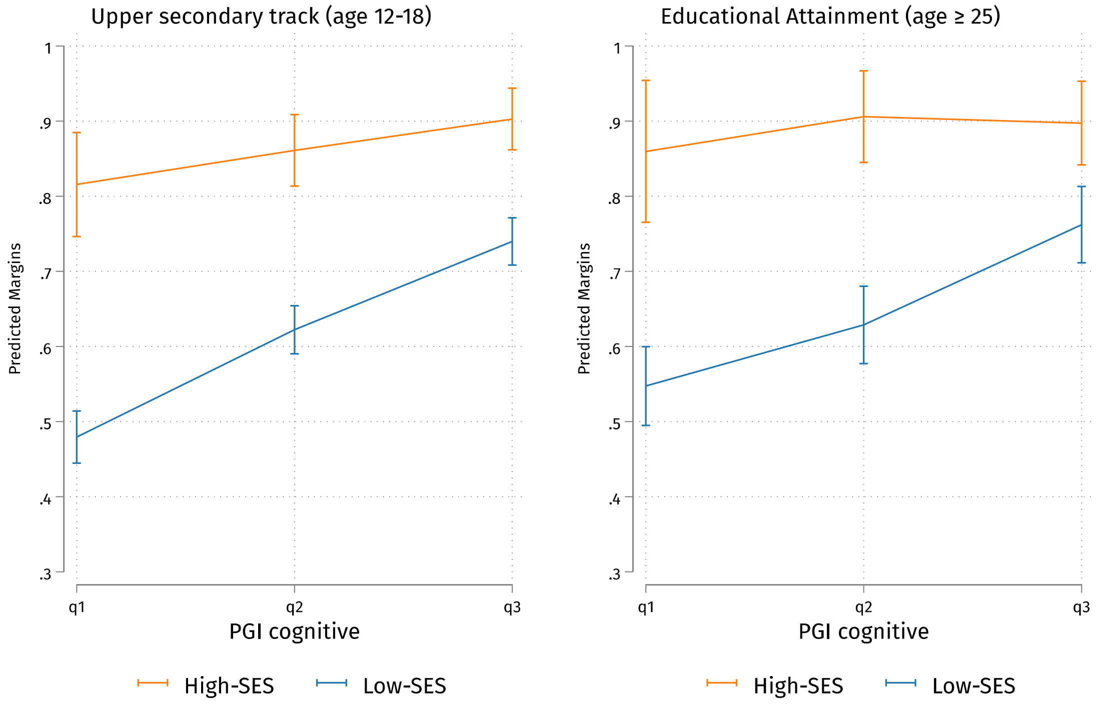


Controls are included. We also include covariates-environment (family SES) and covariates-gene (PGI) interaction (Keller, 2014).

Figure A13: Logistic regression models for dichotomous outcomes variables and with the PGI in terciles in the *between family samples* (without controlling for parents' PGI) for the interaction between family's SES and PGI for non-cognitive skills (average marginal effect, at 95 percent confidence interval)

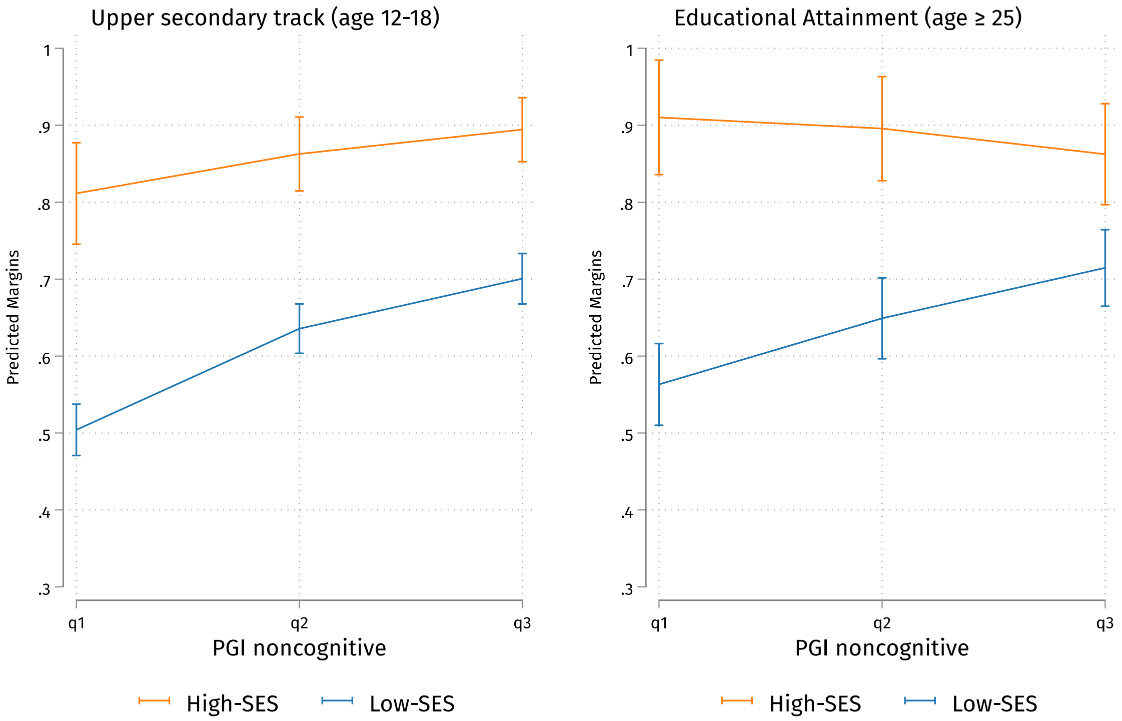


Controls are included. We also include covariates-environment (family SES) and covariates-gene (PGI) interaction (Keller, 2014).

#### 7.2.3.2. Trio design

Figure A14: Logistic regression models for dichotomous outcomes variables (i.e., academic tracking and educational attainment) and with the PGI in terciles in the *trio samples* (controlling for parents' PGI) for the interaction between family's SES and PGI for cognitive skills (average marginal effect, at 95 percent confidence interval)

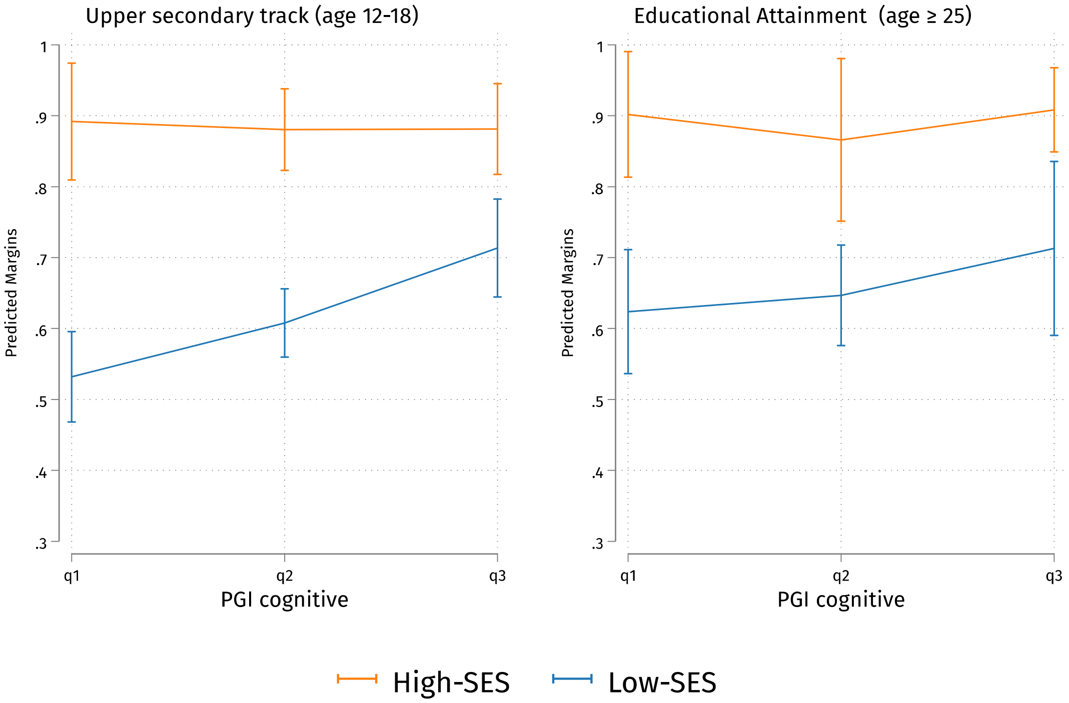


Controls are included. We also include covariates-environment (family SES) and covariates-gene (PGI) interaction (Keller, 2014).

Figure A15: Logistic regression models for dichotomous outcomes variables (i.e., academic tracking and educational attainment) and with the PGI in terciles in the *trio samples* (controlling for parents' PGI) for the interaction between family's SES and PGI for non-cognitive skills (average marginal effect, at 95 percent confidence interval)

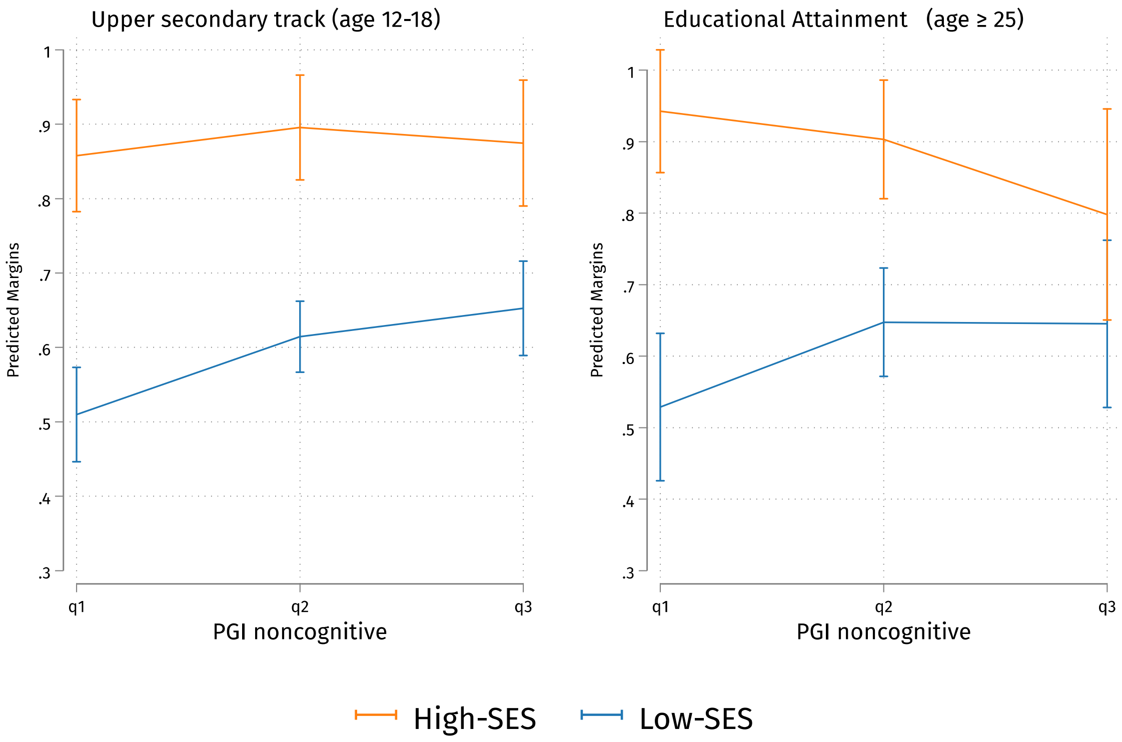


Controls are included. We also include covariates-environment (family SES) and covariates-gene (PGI) interaction (Keller, 2014).

### 7.3. Alternative samples

### 7.3.1. Between-family with all MZ twins

Table A28: OLS (mathematics, reading and CITO) and LPM (academic tracking and educational attainment) regressions to test the association between children's cognitive and non-cognitive PGI and educational outcomes without including family SES.

| | Mathematics (age 7) | Reading (age 7) | Mathematics (age 10) | Reading (age 10) | Test scores CITO (age 12) | Upper secondary track (age 12-18) | Educational Attainment (age ≥ 25) |
|---|---|---|---|---|---|---|---|
| Cognitive PGS | 0.156*** | 0.155*** | 0.211*** | 0.179*** | 0.268*** | 0.115*** | 0.105*** |
| | (0.014) | (0.015) | (0.016) | (0.016) | (0.020) | (0.008) | (0.012) |
| Non-Cognitive PGI | 0.0758*** | 0.0805*** | 0.0976*** | 0.0964*** | 0.190*** | 0.0891*** | 0.0843*** |
| | (0.014) | (0.015) | (0.016) | (0.016) | (0.020) | (0.009) | (0.012) |
| Observations | 5088 | 5134 | 5240 | 5303 | 3611 | 4377 | 1648 |
| Adjusted R2 | 0.0518 | 0.0434 | 0.0708 | 0.0457 | 0.104 | 0.0900 | 0.0790 |

Note: Robust standard errors in parentheses. Two-tailed t-test: + $p < 0.10$, * $p < 0.05$, ** $p < 0.01$, *** $p < 0.000$. Controls included but not reported above: first 10 PCs and Platform.

Table A29: OLS (mathematics, reading and CITO) and LPM (academic tracking and educational attainment) regressions to test the association between children's cognitive and non-cognitive PGI and educational outcomes including family SES.

| | Mathematics (age 7) | Reading (age 7) | Mathematics (age 10) | Reading (age 10) | Test scores CITO (age 12) | Upper secondary track (age 12-18) | Educational Attainment (age ≥ 25) |
|---|---|---|---|---|---|---|---|
| Cognitive PGS | 0.144*** | 0.142*** | 0.192*** | 0.159*** | 0.241*** | 0.102*** | 0.0939*** |
| | (0.014) | (0.016) | (0.016) | (0.016) | (0.020) | (0.008) | (0.012) |
| Non-Cognitive PGI | 0.0643*** | 0.0684*** | 0.0804*** | 0.0786*** | 0.164*** | 0.0775*** | 0.0753*** |
| | (0.014) | (0.015) | (0.016) | (0.016) | (0.020) | (0.009) | (0.012) |
| SES (ref: Low-SES) | 0.179*** | 0.187*** | 0.256*** | 0.263*** | 0.426*** | 0.195*** | 0.167*** |
| | (0.036) | (0.039) | (0.039) | (0.040) | (0.045) | (0.018) | (0.027) |
| Observations | 5088 | 5134 | 5240 | 5303 | 3611 | 4377 | 1648 |
| Adjusted R2 | 0.0583 | 0.0498 | 0.0815 | 0.0572 | 0.131 | 0.114 | 0.0974 |

Note: Robust standard errors in parentheses. Two-tailed t-test: + $p < 0.10$, * $p < 0.05$, ** $p < 0.01$, *** $p < 0.000$. Controls included but not reported above: first 10 PCs and Platform.

Table A30: OLS (mathematics, reading and CITO) and LPM (academic tracking and educational attainment) regressions to test the interaction between children's cognitive and non-cognitive PGI and family SES

| | Mathematics (age 7) | Reading (age 7) | Mathematics (age 10) | Reading (age 10) | Test scores CITO (age 12) | Upper secondary track (age 12-18) | Educational Attainment (age ≥ 25) |
|---|---|---|---|---|---|---|---|
| SES x PGI Cognitive | -0.0285 | 0.0226 | -0.0437 | 0.0404 | -0.0452 | -0.0545** | -0.0954*** |
| | (0.036) | (0.040) | (0.041) | (0.041) | (0.047) | (0.018) | (0.028) |
| Observations | 5088 | 5134 | 5240 | 5303 | 3611 | 4377 | 1648 |
| Adjusted R2 | 0.0603 | 0.0532 | 0.0818 | 0.0584 | 0.133 | 0.115 | 0.101 |

| | | | | | | | |
|---|---|---|---|---|---|---|---|
| SES x PGI | | | | | | | |
| Non-Cognitive | -0.00819 | 0.00576 | -0.0201 | 0.00121 | -0.0726+ | -0.0463* | -0.0814** |
| | (0.037) | (0.041) | (0.038) | (0.039) | (0.044) | (0.019) | (0.028) |
| Observations | 5088 | 5134 | 5240 | 5303 | 3611 | 4377 | 1648 |
| Adjusted R2 | 0.0612 | 0.0515 | 0.0830 | 0.0591 | 0.133 | 0.118 | 0.104 |

Note: Robust standard errors in parentheses. Two-tailed t-test: + $p < 0.10$, * $p < 0.05$, ** $p < 0.01$, *** $p < 0.000$.
Controls included but not reported above: first 10 PCs and Platform.

### 7.3.2. Trio analysis with all MZ twins

Table A31: OLS (mathematics, reading and CITO) and LPM (academic tracking and educational attainment) regressions to test the association between children's cognitive and non-cognitive PGI and educational outcomes using the sample of the trio-design, controlling for family SES but without parents cognitive and non-cognitive PGI.

| | Mathematics (age 7) | Reading (age 7) | Mathematics (age 10) | Reading (age 10) | Test scores CITO (age 12) | Upper secondary track (age 12-18) | Educational Attainment (age ≥ 25) |
|---|---|---|---|---|---|---|---|
| Cognitive PGI | 0.144*** | 0.148*** | 0.170*** | 0.151*** | 0.201*** | 0.0961*** | 0.0697*** |
| | (0.021) | (0.023) | (0.023) | (0.023) | (0.028) | (0.012) | (0.019) |
| NonCognitive PGI | 0.0685** | 0.0770*** | 0.0830*** | 0.0813*** | 0.119*** | 0.0662*** | 0.0617*** |
| | (0.021) | (0.022) | (0.023) | (0.022) | (0.028) | (0.013) | (0.018) |
| SES (ref: Low-SES) | 0.251*** | 0.222*** | 0.303*** | 0.272*** | 0.480*** | 0.221*** | 0.222*** |
| | (0.052) | (0.055) | (0.054) | (0.054) | (0.062) | (0.026) | (0.035) |
| Observations | 2570 | 2586 | 2798 | 2835 | 1683 | 1984 | 776 |
| Adjusted R2 | 0.0576 | 0.0526 | 0.0577 | 0.0479 | 0.112 | 0.127 | 0.0840 |

Note: Robust standard errors in parentheses. Two-tailed t-test: + $p < 0.10$, * $p < 0.05$, ** $p < 0.01$, *** $p < 0.000$.
Controls included but not reported above: first 10 PCs and Platform.

Table A32: OLS (mathematics, reading and CITO) and LPM (academic tracking and educational attainment) regressions to test the association between children's cognitive and non-cognitive PGI and educational outcomes using the sample of the trio-design, controlling for family SES and parents cognitive and non-cognitive PGI.

| | Mathematics (age 7) | Reading (age 7) | Mathematics (age 10) | Reading (age 10) | Test scores CITO (age 12) | Upper secondary track (age 12-18) | Educational Attainment (age ≥ 25) |
|---|---|---|---|---|---|---|---|
| Cognitive PGI | 0.136*** | 0.158*** | 0.218*** | 0.188*** | 0.178*** | 0.0799*** | 0.0642* |
| | (0.030) | (0.031) | (0.033) | (0.032) | (0.041) | (0.017) | (0.026) |
| NonCognitive PGI | 0.0667* | 0.0555+ | 0.0464 | 0.0565+ | 0.0737+ | 0.0383* | 0.0616* |
| | (0.030) | (0.032) | (0.034) | (0.034) | (0.041) | (0.017) | (0.025) |
| SES (ref: Low-SES) | 0.248*** | 0.221*** | 0.309*** | 0.279*** | 0.464*** | 0.211*** | 0.221*** |
| | (0.053) | (0.056) | (0.054) | (0.054) | (0.063) | (0.026) | (0.036) |
| Cognitive PGI Mother | 0.0135 | -0.00462 | -0.0518+ | -0.0373 | 0.0413 | 0.00416 | 0.0128 |
| | (0.027) | (0.028) | (0.028) | (0.029) | (0.037) | (0.016) | (0.022) |
| Cognitive PGI Father | 0.00317 | -0.0152 | -0.0444 | -0.0380 | 0.00393 | 0.0252 | 0.000370 |
| | (0.027) | (0.028) | (0.031) | (0.029) | (0.036) | (0.016) | (0.023) |
| NonCognitive PGI Father | 0.00584 | 0.00857 | 0.0398 | 0.00775 | 0.0473 | 0.0422** | 0.00475 |

| | | | | | | | |
|---|---|---|---|---|---|---|---|
| | (0.026) | (0.027) | (0.030) | (0.030) | (0.035) | (0.015) | (0.023) |
| NonCognitive PGI Mother | -0.00211 | 0.0338 | 0.0333 | 0.0422 | 0.0405 | 0.00977 | -0.00331 |
| | (0.026) | (0.028) | (0.029) | (0.029) | (0.037) | (0.016) | (0.024) |
| Observations | 2570 | 2586 | 2798 | 2835 | 1683 | 1984 | 776 |
| Adjusted R2 | 0.0564 | 0.0522 | 0.0609 | 0.0498 | 0.112 | 0.131 | 0.0800 |

Note: Robust standard errors in parentheses. Two-tailed t-test: $^{+}$ $p < 0.10$, $^{*}$ $p < 0.05$, $^{**}$ $p < 0.01$, $^{***}$ $p < 0.000$. Controls included but not reported above: first 10 PCs and Platform.

Table A33: OLS (mathematics, reading and CITO) and LPM (academic tracking and educational attainment) regressions to test the interaction between children's cognitive and non-cognitive PGI and family's SES using the sample of the trio-design, controlling for family SES and parents cognitive and non-cognitive PGI.

| | Mathematics (age 7) | Reading (age 7) | Mathematics (age 10) | Reading (age 10) | Test scores CITO (age 12) | Upper secondary track (age 12-18) | Educational Attainment (age ≥ 25) |
|---|---|---|---|---|---|---|---|
| High-SES x Cognitive PGI | -0.0548 | -0.00163 | -0.153$^{*}$ | -0.0376 | -0.0350 | -0.0628$^{+}$ | -0.0235 |
| | (0.070) | (0.079) | (0.077) | (0.079) | (0.089) | (0.034) | (0.049) |
| High-SES x NonCognitive PGI | -0.0515 | -0.102 | -0.0281 | -0.0187 | -0.189$^{*}$ | -0.0706$^{+}$ | -0.123$^{*}$ |
| | (0.071) | (0.081) | (0.076) | (0.077) | (0.089) | (0.036) | (0.052) |
| Adjusted R2 | 0.0668 | 0.0599 | 0.0692 | 0.0486 | 0.112 | 0.132 | 0.0921 |

Note: Robust standard errors in parentheses. Two-tailed t-test: $^{+}$ $p < 0.10$, $^{*}$ $p < 0.05$, $^{**}$ $p < 0.01$, $^{***}$ $p < 0.000$. Controls included but not reported above: first 10 PCs and Platform.

### 7.3.3. Educational attainment: all participants, those born before 1980, those born after 1980

In this section, we first repeat the analysis for educational attainment using different samples. Specifically, we compare the main results (those born after 1980) with the results obtained looking also at those born before 1980 and then only to those born before 1980.

#### 7.3.3.1 Between-family design

Table A34: LPM regressions to test the association between children's cognitive and non-cognitive PGI and educational attainment controlling for family SES in the *between-family design* in the three different samples

| | Educational Attainment (age ≥ 25) Overall sample | Educational Attainment (age ≥ 25) Prior to 1980 | Educational Attainment (age ≥ 25) After 1980 |
|---|---|---|---|
| SES (ref: Low-SES) | 0.318*** | 0.353*** | 0.192*** |
| | (0.018) | (0.023) | (0.026) |
| Cognitive PGS | 0.0890*** | 0.0860*** | 0.0957*** |
| | (0.008) | (0.009) | (0.013) |
| Non-Cognitive PGS | 0.0879*** | 0.0939*** | 0.0686*** |
| | (0.008) | (0.009) | (0.014) |

| | | | |
|---|---|---|---|
| Observations | 4541 | 3317 | 1224 |

Note: Robust standard errors in parentheses. Two-tailed t-test: $^{+}p < 0.10$, $^{*}p < 0.05$, $^{**}p < 0.01$, $^{***}p < 0.000$. Controls included but not reported above: first 10 PCs and Platform.

Table A35: LPM regressions to test the interaction between children's cognitive and non-cognitive PGI and family SES in the *between-family design* in the three different samples

| | Educational Attainment (age ≥ 25) Overall sample | Educational Attainment (age ≥ 25) Prior to 1980 | Educational Attainment (age ≥ 25) After 1980 |
|---|---|---|---|
| SES x PGS Cognitive | -0.0424* | -0.0137 | -0.110*** |
| | (0.017) | (0.021) | (0.028) |
| SES x PGS Non-Cognitive | -0.0773*** | -0.0461+ | -0.111*** |
| | (0.019) | (0.024) | (0.029) |
| Observations | 4541 | 3317 | 1224 |

Note: Robust standard errors in parentheses. Two-tailed t-test: $^{+}p < 0.10$, $^{*}p < 0.05$, $^{**}p < 0.01$, $^{***}p < 0.000$. Controls included but not reported above: first 10 PCs and Platform. We also include covariates-environment (family SES) and covariates-gene (PGI) interaction (Keller, 2014).

## 7.3.3.2 Within-family design

Table A36: Family-fixed effect regressions to test the association between children's cognitive and non-cognitive PGI on educational attainment in the *within-family design* in the three different samples

| | Educational Attainment (age ≥ 25) Overall sample | Educational Attainment (age ≥ 25) Prior to 1980 | Educational Attainment (age ≥ 25) After 1980 |
|---|---|---|---|
| Cognitive PGS | 0.0714*** | 0.0732*** | 0.0700 |
| | (0.018) | (0.021) | (0.043) |
| Non-Cognitive PGS | 0.0582** | 0.0551** | 0.0966* |
| | (0.019) | (0.021) | (0.046) |
| Observations | 2030 | 1566 | 426 |

Note: Robust standard errors in parentheses. Two-tailed t-test: $^{+}p < 0.10$, $^{*}p < 0.05$, $^{**}p < 0.01$, $^{***}p < 0.000$. Controls included but not reported above: first 10 PCs and Platform.

Table A37: Family-fixed effect regressions to test the interaction between children's cognitive and non-cognitive PGI and family SES in the *within-family design* in the three different samples

| | Educational Attainment (age ≥ 25) Overall sample | Educational Attainment (age ≥ 25) Prior to 1980 | Educational Attainment (age ≥ 25) After 1980 |
|---|---|---|---|
| SES x PGS Cognitive | 0.0127 | 0.0898 | -0.0414 |
| | (0.041) | (0.059) | (0.094) |
| SES x PGS Non-Cognitive | 0.00811 | 0.157* | -0.0881 |
| | (0.050) | (0.068) | (0.088) |
| Observations | 2030 | 1566 | 426 |

Note: Robust standard errors in parentheses. Two-tailed t-test: $^{+}p < 0.10$, $^{*}p < 0.05$, $^{**}p < 0.01$, $^{***}p < 0.000$. Controls included but not reported above: first 10 PCs and Platform. We also include covariates-environment (family SES) and covariates-gene (PGI) interaction (Keller, 2014).

### 7.3.3.3. Trio design

Table A38: LPM regressions to test the interaction between children's cognitive and non-cognitive PGI and family SES in the *trio design* in the three different samples

| | Educational Attainment (age ≥ 25) Overall sample | Educational Attainment (age ≥ 25) Prior to 1980 | Educational Attainment (age ≥ 25) After 1980 |
|---|---|---|---|
| Cognitive PGS | 0.0399* | 0.0267 | 0.0780** |
| | (0.016) | (0.020) | (0.028) |
| Non-Cognitive PGS | 0.0190 | 0.00649 | 0.0649* |
| | (0.017) | (0.020) | (0.031) |
| SES (ref: Low-SES) | 0.283*** | 0.297*** | 0.227*** |
| | (0.025) | (0.033) | (0.038) |
| Cognitive PGI Mother | 0.0452** | 0.0553** | 0.00620 |
| | (0.015) | (0.018) | (0.025) |
| Cognitive PGI Father | 0.0388* | 0.0538** | 0.0130 |
| | (0.016) | (0.020) | (0.025) |
| Noncognitive PGI Father | 0.0682*** | 0.0887*** | 0.00523 |
| | (0.015) | (0.018) | (0.026) |
| Noncognitive PGI Mother | 0.0439** | 0.0643*** | -0.0160 |
| | (0.015) | (0.018) | (0.025) |
| Observations | 1783 | 1207 | 576 |

Note: Robust standard errors in parentheses. Two-tailed t-test: $^{+}$ $p$ < 0.10, $^{*}$ $p$ < 0.05, $^{**}$ $p$ < 0.01, $^{***}$ $p$ < 0.000. Controls included but not reported above: first 10 PCs and Platform.

Table A39: LPM regressions to test the interaction between children's cognitive and non-cognitive PGI and family SES in the *trio design* in the three different samples

| | Educational Attainment (age ≥ 25) Overall sample | Educational Attainment (age ≥ 25) Prior to 1980 | Educational Attainment (age ≥ 25) After 1980 |
|---|---|---|---|
| SES x PGS Cognitive | 0.0301 | 0.0856+ | -0.0617 |
| | (0.036) | (0.051) | (0.055) |
| SES x PGS Non-Cognitive | 0.00305 | 0.0836 | -0.130+ |
| | (0.045) | (0.066) | (0.066) |
| Observations | 1783 | 1207 | 576 |

Note: Robust standard errors in parentheses. Two-tailed t-test: $^{+}$ $p$ < 0.10, $^{*}$ $p$ < 0.05, $^{**}$ $p$ < 0.01, $^{***}$ $p$ < 0.000. Controls included but not reported above: first 10 PCs and Platform. We also include covariates-environment (family SES) and covariates-gene (PGI) interaction (Keller, 2014).

## 7.4. Alternative measure of SES: Parents' occupation

### 7.4.1. Between-family design

Table A40: OLS and LPM (academic tracking and educational attainment) regressions to test the interaction between children's cognitive and non-cognitive PGI and family SES on educational outcomes in the *between-family analysis*.

| | Mathematics (age 7) | Reading (age 7) | Mathematics (age 10) | Reading (age 10) | Test scores CITO (age 12) | Upper secondary track (age 12-18) | Educational Attainment (age ≥ 25) |
|---|---|---|---|---|---|---|---|
| SES x PGI Cognitive | -0.0341 | -0.0120 | -0.0815+ | -0.0404 | -0.0815 | -0.0662*** | -0.146** |
| | (0.043) | (0.049) | (0.047) | (0.046) | (0.051) | (0.020) | (0.045) |
| SES x PGI Non-Cognitive | 0.0284 | 0.0400 | -0.0448 | -0.0114 | -0.0502 | -0.0519** | -0.119** |
| | (0.043) | (0.047) | (0.043) | (0.045) | (0.044) | (0.018) | (0.038) |
| Observations | 3728 | 3756 | 3829 | 3875 | 2647 | 3225 | 900 |
| Adjusted R2 | 0.0505 | 0.0441 | 0.0615 | 0.0449 | 0.130 | 0.120 | 0.0701 |

Note: Robust standard errors in parentheses. Two-tailed t-test: $^{+}p < 0.10$, $^{*}p < 0.05$, $^{**}p < 0.01$, $^{***}p < 0.000$. Controls included but not reported above: first 10 PCs and Platform. We also include covariates-environment (family SES) and covariates-gene (PGI) interaction (Keller, 2014).

### 7.4.2. Within-family design

Table A41: Family-fixed effect regressions to test the interaction between children's cognitive and non-cognitive PGI and family SES on educational outcomes in the *within-family analysis*.

| | Mathematics (age 7) | Reading (age 7) | Mathematics (age 10) | Reading (age 10) | Test scores CITO (age 12) | Upper secondary track (age 12-18) | Educational Attainment (age ≥ 25) |
|---|---|---|---|---|---|---|---|
| SES x PGI Cognitive | 0.0273 | 0.0539 | -0.197* | 0.0519 | -0.131 | -0.0895* | -0.0283 |
| | (0.094) | (0.082) | (0.094) | (0.096) | (0.081) | (0.035) | (0.139) |
| SES x PGI Non-Cognitive | -0.0153 | 0.136 | -0.195* | -0.0630 | -0.000932 | -0.0131 | 0.0423 |
| | (0.109) | (0.086) | (0.099) | (0.095) | (0.101) | (0.038) | (0.107) |
| Observations | 2124 | 2130 | 2212 | 2236 | 1494 | 1976 | 342 |
| Adjusted R2 | 0.0435 | 0.0278 | 0.0532 | 0.0248 | 0.0681 | 0.0374 | 0.172 |

Note: Robust standard errors in parentheses. Two-tailed t-test: $^{+}p < 0.10$, $^{*}p < 0.05$, $^{**}p < 0.01$, $^{***}p < 0.000$. Controls included but not reported above: first 10 PCs and Platform. We also include covariates-environment (family SES) and covariates-gene (PGI) interaction (Keller, 2014).

### 7.4.3. Trio design

Table A42: OLS and LPM (academic tracking and educational attainment) regressions to test the interaction between children's cognitive PGI and family SES on educational outcomes in the *trio analysis*.

| | Mathematics (age 7) | Reading (age 7) | Mathematics (age 10) | Reading (age 10) | Test scores CITO (age 12) | Upper secondary track (age 12-18) | Educational Attainment (age ≥ 25) |
|---|---|---|---|---|---|---|---|
| SES x PGI Cognitive | -0.0756 | 0.0184 | -0.0388 | -0.0186 | -0.120 | -0.0965* | -0.168* |
| | (0.075) | (0.081) | (0.084) | (0.085) | (0.105) | (0.039) | (0.085) |
| SES x PGI Non-Cognitive | -0.00675 | 0.00894 | -0.0271 | 0.00522 | -0.0917 | -0.0193 | -0.208** |
| | (0.084) | (0.087) | (0.081) | (0.077) | (0.096) | (0.034) | (0.072) |
| Observations | 1861 | 1869 | 2022 | 2051 | 1248 | 1475 | 452 |
| Adjusted R2 | 0.0501 | 0.0462 | 0.0585 | 0.0458 | 0.100 | 0.125 | 0.0715 |

Note: Robust standard errors in parentheses. Two-tailed t-test: $^{+}$ $p$ < 0.10, $^{*}$ $p$ < 0.05, $^{**}$ $p$ < 0.01, $^{***}$ $p$ < 0.000. Controls included but not reported above: first 10 PCs and Platform. We also include covariates-environment (family SES) and covariates-gene (PGI) interaction (Keller, 2014).

## 7.5. Educational attainment as continuous outcome

We repeat the analysis without dichotomising educational attainment by using it in four categories as originally provided by NTR (1: primary school only, lower vocational school and lower secondary school, intermediate vocational school and intermediate or higher secondary school, higher vocational school and university).

### 7.5.1 Between-family design

Table A43: LPM regression models to test the interaction between children's cognitive and non-cognitive PGI and family SES in the *between design* in the three different samples and using educational attainment not dichotomized

| | Educational Attainment (age ≥ 25) Overall sample | Educational Attainment (age ≥ 25) Prior to 1980 | Educational Attainment (age ≥ 25) After 1980 |
|---|---|---|---|
| SES x PGI Cognitive | -0.0645** | -0.0290 | -0.153*** |
| | (0.025) | (0.032) | (0.035) |
| SES x PGI Non-Cognitive | -0.122*** | -0.0857** | -0.158*** |
| | (0.024) | (0.031) | (0.035) |
| Observations | 4541 | 3317 | 1224 |
| Adjusted R2 | 0.103 | 0.0988 | 0.0936 |

Note: Robust standard errors in parentheses. Two-tailed t-test: $^{+}$ $p$ < 0.10, $^{*}$ $p$ < 0.05, $^{**}$ $p$ < 0.01, $^{***}$ $p$ < 0.000. Controls included but not reported above: first 10 PCs and Platform. We also include covariates-environment (family SES) and covariates-gene (PGI) interaction (Keller, 2014).

### 7.5.2 Within-family design

Table A44: Family-fixed effect regressions to test the interaction between children's non-cognitive PGI and family SES in the *within-family design* in the three different samples and using educational attainment not dichotomized

| | Educational Attainment (age ≥ 25) Overall sample | Educational Attainment (age ≥ 25) Prior to 1980 | Educational Attainment (age ≥ 25) After 1980 |
|---|---|---|---|
| SES x PGI Cognitive | 0.0127 | 0.0135 | -0.0414 |
| | (0.041) | (0.059) | (0.094) |
| SES x PGI Non-Cognitive | 0.00811 | 0.111+ | -0.0881 |
| | (0.050) | (0.067) | (0.088) |
| Observations | 2030 | 1566 | 426 |
| Adjusted R2 | 0.0154 | 0.0157 | 0.120 |

Note: Robust standard errors in parentheses. Two-tailed t-test: $^{+}$ $p < 0.10$, $^{*}$ $p < 0.05$, $^{**}$ $p < 0.01$, $^{***}$ $p < 0.000$. Controls included but not reported above: first 10 PCs and Platform. We also include covariates-environment (family SES) and covariates-gene (PGI) interaction (Keller, 2014).

### 7.5.3 Trio design

Table A45: LPM regression models to test the interaction between children's cognitive and non-cognitive PGI and family SES in the *trio design* in the three different samples and using educational attainment not dichotomized

| | Educational Attainment (age ≥ 25) Overall sample | Educational Attainment (age ≥ 25) Prior to 1980 | Educational Attainment (age ≥ 25) After 1980 |
|---|---|---|---|
| SES x PGI Cognitive | 0.0301 | 0.0856+ | -0.0617 |
| | (0.036) | (0.051) | (0.055) |
| SES x PGI Non-Cognitive | 0.00305 | 0.0836 | -0.130+ |
| | (0.045) | (0.066) | (0.066) |
| Observations | 1783 | 1207 | 576 |
| Adjusted R2 | 0.133 | 0.143 | 0.0911 |

Note: Robust standard errors in parentheses. Two-tailed t-test: $^{+}$ $p < 0.10$, $^{*}$ $p < 0.05$, $^{**}$ $p < 0.01$, $^{***}$ $p < 0.000$. Controls included but not reported above: first 10 PCs and Platform. We also include covariates-environment (family SES) and covariates-gene (PGI) interaction (Keller, 2014).
